# Single-cell multiomics of neuronal activation reveals context-dependent genetic control of brain disorders

**DOI:** 10.1101/2025.02.17.638682

**Published:** 2025-02-17

**Authors:** Lifan Liang, Siwei Zhang, Zicheng Wang, Hanwen Zhang, Chuxuan Li, Alexandra C. Duhe, Xiaotong Sun, Xiaoyuan Zhong, Alena Kozlova, Brendan Jamison, Whitney Wood, Zhiping P. Pang, Alan R. Sanders, Xin He, Jubao Duan

**Affiliations:** 1 Department of Human Genetics, The University of Chicago, Chicago, IL 60637, USA.; 2 Center for Psychiatric Genetics, Endeavor Health Research Institute, Evanston, IL 60201, USA.; 3 Graduate Group in Genomics and Computational Biology, Perelman School of Medicine, University of Pennsylvania, Philadelphia, PA 19104, USA; 4 Department of Neuroscience and Cell Biology, Child Health Institute of New Jersey, Rutgers Robert Wood Johnson Medical School, New Brunswick, NJ 08901, USA.; 5 Department of Psychiatry and Behavioral Neuroscience, The University of Chicago, Chicago, IL 60637, USA.

## Abstract

Despite hundreds of genetic risk loci identified for neuropsychiatric disorders (NPD), most causal variants/genes remain unknown. A major hurdle is that disease risk variants may act in specific biological contexts, e.g., during neuronal activation, which is difficult to study *in vivo* at the population level. Here, we conducted a single-cell multiomics study of neuronal activation (stimulation) in human iPSC-induced excitatory and inhibitory neurons from 100 donors, and uncovered abundant neuronal stimulation-specific causal variants/genes for NPD. We surveyed NPD-relevant transcriptomic and epigenomic landscape of neuronal activation and identified thousands of genetic variants associated with activity-dependent gene expression (i.e., eQTL) and chromatin accessibility (i.e., caQTL). These caQTL explained considerably larger proportions of NPD heritability than the eQTL. Integrating the multiomic data with GWAS further revealed NPD risk variants/genes whose effects were only detected upon stimulation. Interestingly, multiple lines of evidence support a role of activity-dependent cholesterol metabolism in NPD. Our work highlights the power of cell stimulation to reveal context-dependent “hidden” genetic effects.

## Main Text

Genome-wide association studies (GWAS) of neurodevelopmental and psychiatric disorders (NPD) have identified hundreds of risk loci, with over 280 for schizophrenia (SCZ)(1–11). These genetic findings provide unprecedented opportunities for studying disease mechanisms. However, despite extensive functional genomics study in postmortem brains (e.g., PsychENCODE)(12–15) and in human induced pluripotent stem cell (iPSC)-induced neurons from large cohorts(16, 17), most causal variants/genes for NPD remain unknown. A major hurdle for identifying causal variants/genes of complex disorders is that most GWAS risk variants are in noncoding regions that lack functional interpretation. More importantly, noncoding regulatory variants often act in specific biological contexts(18–21), e.g., disease variants may only show detectable function in response to stimuli, as found for some other disorders(18, 22). It is thus important to understand NPD genetic risk in specific biological contexts such as neuronal activation.

Neuronal activity regulates neurodevelopment and synaptic plasticity(23), processes important in the development of NPD. Neuronal activation in response to neurotransmitters leads to Ca^2+^ influx that activates early response genes (ERGs; e.g., *Fos*) and other activating protein complex 1 (AP-1) family transcription factors (TFs), which further drive the activation of late response genes (LRGs; e.g., *Bdnf*)(23, 24). In mouse, neural activity induces epigenomic alterations of open chromatin regions (OCRs), accompanied by expression changes of thousands of LRG genes within 4–6 hrs(25–29). *In vitro*, stimuli such as membrane-depolarizing levels of potassium chloride (KCl)(23, 25, 26, 30, 31) cause neuronal activation that mimics the *in vivo* effects of social experiences, stress, or drugs of abuse(23). With KCI stimulation in human iPSC-induced neurons (iNs), recent studies show extensive activity-dependent transcriptomic and epigenomic alterations(32, 33). However, due to the lack of single-neuron assay modality and the limited sample size (n=2–4) in previous studies(32, 33), genetic regulation of cell type- and individual-specific responses to neuronal stimulation as well as its functional relevance to NPD remain elusive.

**Fig. 1.**
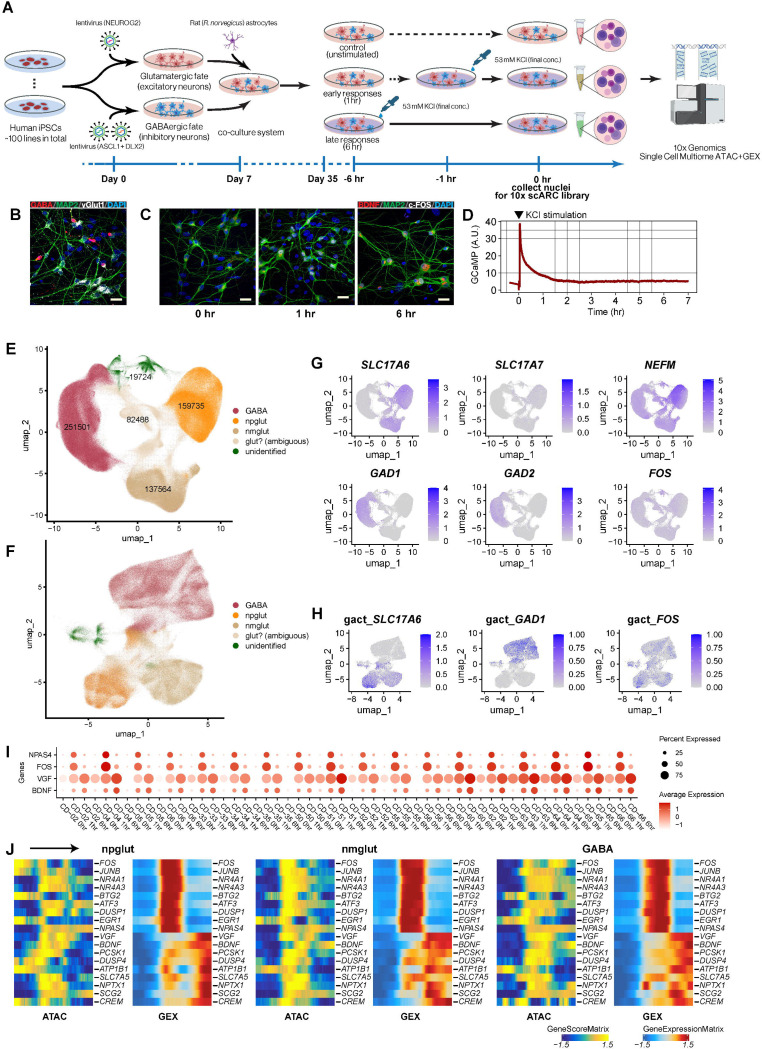
Single-nucleus multiomic assay of cell co-cultures that model neuronal activation. **(A)** Schematic of the experimental design. Co-cultures of excitatory and inhibitory neurons with rat astrocytes were stimulated by KCI to mimic neuronal activation. **(B)** IF staining of the co-cultures shows glutamatergic excitatory neurons (vGlut1+) and GABAergic inhibitory neurons (GABA+). MAP2+, neurons; DAPI, nuclei. **(C)** IF staining of early (1 hr) response gene *c-FOS* and late (6 hrs) response gene *BDNF*. DAPI, nuclei. Scale bar=25 μm. **(D)** Ca^2+^ influx spike (i.e., fluorescence intensity of GCaMP) upon KCl stimulation. **(E)** UMAP projection and cell identities for snRNA-seq data of 100 lines. **(F)** UMAP projection for snATAC-seq data (cell identify label was transferred from snRNA-seq). **(G)** and **(H)** Feature plots of gene expression (snRNA-seq) and gene activity score (snATAC-seq) for cell-type-specific genes (*SLC17A6* and *SLC17A7* for iGlut, *GAD1* and *GAD2* for i*GABA*, *NEFM* for two subclusters of iGlut) and *FOS*. **(I)** Reproducible expression dynamics of early (*NPAS4, FOS*) and late (*VGF, BDNF*) response genes from 0, 1, to 6 hrs across cell lines (shown are 18 lines). (J) Pseudo-time trajectories of gene expression and chromatin accessibility (GeneScore) of selected early and later response genes in each cell type.

Here, we carried out single-nucleus multiomics profiling (RNA sequencing and assay for transposase-accessible chromatin with sequencing; snRNA-seq/ATAC-seq) of neuron activity-dependent transcriptomic and chromatin accessibility changes in co-cultured human excitatory and inhibitory iNs from 100 donors (Fig. 1A). We obtained a comprehensive neuron subtype-specific landscape of activity-dependent transcriptomic and chromatin accessibility changes. The multi-modality assay of single neurons allows us to identify gene regulatory networks (GRN) controlling neuronal activation, shedding light on the role of neuron activity-dependent TFs important for NPD. This large iPSC cohort enables us to map thousands of cell-type-specific and activity-dependent quantitative trait loci for gene expression (eQTL) and chromatin accessibility (caQTL). These data helped us to infer putative NPD risk variants and genes that manifest functional effects only upon neuronal stimulation.

## Results

### Multiomic profiling of neuronal activation in human excitatory and inhibitory neurons

To profile neuron activity-dependent transcriptomics/epigenomics, we modeled neuronal activation by KCl stimulation in a large cohort of co-cultures of human iPSC-derived glutamatergic neurons (iGlut, excitatory)(34) and GABAergic neurons (iGABA, inhibitory)(35, 36) (Fig. 1A), a design that can better recapitulate the *in vivo* neuronal context than previous studies(32, 33) that cultured excitatory and inhibitory neurons in isolation. Day-35 iGlut and iGABA neurons, co-cultured with rat astrocytes, were stimulated by KCl (53 mM) for 1 or 6 hrs(28, 29, 31, 37) to capture changes of ERGs and LRGs, respectively, followed by 10x Genomics single-nucleus Multiomics profiling (snRNA-seq/ATAC-seq) (Fig. 1A). We first verified the iGlut or iGABA neuronal identity and purity as well as the expected expression dynamics of *FOS* (an ERG) and *BDNF* (an LRG) by immunostaining of neural co-cultures upon KCI stimulation (Fig. 1B-C). We also examined the robust Ca^2+^ spikes via GCaMP signals immediately after KCI stimulation in GCaMP-infected neurons (Fig. 1D), confirming an effective neuronal activation in our neural co-culture model.

We successfully differentiated 100 iPSC lines into iGlut and iGABA and obtained multiomics data from neural co-cultures at 0, 1, and 6 hrs after KCl stimulation (Table S1). We analyzed 1,053,422 nuclei (Tables S1-2), of which 651,012 passed stringent snRNA/ATAC-seq quality control (QC) (Fig. S1A-I, Fig. S2A-D, Fig. S3A-E, Fig. S4A-C, Table S2-3, Supplementary text). We defined three major subtypes of neurons: GABA (n=251,501), NEFM+ Glut (npglut; with stronger NEFM expression) (n=159,735) and NEFM- Glut (nmglut; with weaker NEFM expression) (n=137,564) (Fig. 1E-H, Fig. S4D-F, Table S1), showing reproducible cell type clustering patterns across samples (Fig. S3A-E). Compared to single-cell transcriptomic profiles of human brain excitatory and inhibitory neurons(38), our iGlut and iGABA are mostly similar to neurons of early brain developmental stages (from 2^nd^ trimester to 2 years old; predominately from 2^nd^ trimester) (Fig. S5A-F). We also found comparable proportions of subtypes of neurons between time points (Fig. S6A).

We first examined the changes of expression and chromatin accessibility of some known ERGs and LRGs. As expected, ERGs (e.g., *FOS*) expression rapidly increased from 0 to 1 hr and diminished at 6 hrs of KCI stimulation, while LRGs (e.g., *BDNF*) expression often peaked at 6 hrs (Fig. 1I, Fig. S6B-C). However, their chromatin accessibility did not seem to follow expression dynamics, with most ERGs exhibiting a prolonged chromatin openness till 6 hrs while most LRGs showing robust chromatin openness at 1 hr before their expression peaked at 6 hrs (Fig. S6B-C). The observed discordance between gene expression and chromatin accessibility at discrete time points was further confirmed by a continuous pseudo time trajectory analysis of single-cell gene chromatin accessibility score and gene expression (Fig. S6D). ERGs and LRGs exhibited expected expression dynamics along the pseudo time trajectory while their gene score matrix often showed a discordant pattern (Fig. 1J). These results validated our co-culture cellular model for studying neuronal activity-dependent gene expression and highlighted the complexity of activity-dependent chromatin regulation.

We next surveyed transcriptomic and epigenomic landscapes of cell-type-specific neuronal activation and their relevance to NPD. Differentially expressed gene (DEG) analysis of snRNA-seq showed that 65–82% genes were either up- or downregulated in any cell type/time point (1 or 6 hrs), with the well-known ERGs (e.g., *FOS*, *FOSB*) exhibiting the largest FC (Fig. S7, Table S4, Supplementary text). Interestingly, only the upregulated genes showed significant enrichment for synaptic genes (Fig. S8A), rare risk genes of SCZ (39) and ASD (40) (Fig. S8B-E, Table S5), and for common GWAS risk of NPD or traits with strongest enrichment for SCZ (Fig. S9A). For the epigenomic landscape, analyzing snATAC-seq (Fig. 1F, Fig. S1H) identified 150K to 300K OCR peaks across cell types/timepoints (Fig. S9B), of which 19–22% of peaks were stimulation-specific (Fig. S9C, Supplementary text). About 26–34% and 40–51% of the OCR peaks showed differential accessibility (DA) at 1 hr and 6 hrs, respectively (Fig. S10A-C; Table S6). For the ERG *FOS*, we identified a stimulation-specific DA peak ~4.2 kb downstream of its TSS that may drive its early response through chromatin accessibility to CEBP binding (Fig. S10C-D). For *BDNF*, a LRG and also a SCZ risk gene(41), we identified DA peaks that showed peak-gene linkage and might regulate cell type-specific activity-dependent *BDNF* expression (Fig. S9D, Fig. S11A, Table S6, Supplementary text), and further confirmed the regulatory role of a stimulation-specific peak in iGlut by CRISPR/Cas9 editing (Fig. S9E-F, Fig. S11B-C). Finally, analysis of DA peak enrichment for SNP heritability of NPD (Fig. S10E-F) showed that only the DA peaks at 1 or 6 hrs of stimulation but not those static peaks (i.e., chromatin accessibility unaltered) showed GWAS enrichments (Fig. S10E-F). Taken together, our results highlight the widespread effects of neuronal stimulation on cell-type-specific transcriptomes and chromatin accessibility as well as their relevance to NPD.

### Integrative multiomic analyses reveal complex patterns of transcriptomic and epigenomic regulation of neuronal activation

**Fig. 2.**
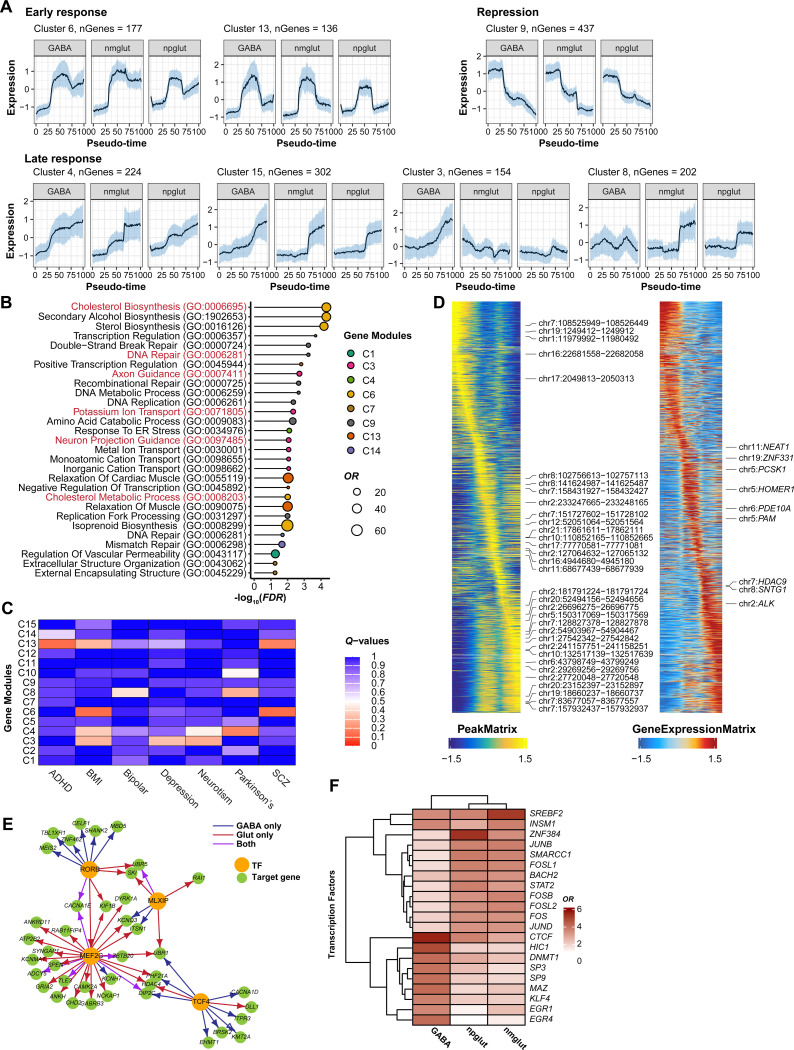
Complex transcriptomic and epigenomics regulation of neuronal activation and activity-dependent gene regulatory network (GRN). **(A)** Pseudo-time trajectories of gene expression of some selected gene modules (clusters) during neuronal activation. Black line, average normalized expression of all genes in a cluster; blue shading, 50% quantile. **(B)** GO term (biological processes) enrichment for gene clusters. Terms with FDR < 0.05 are shown. **(C)** Enrichment of GWAS genes of seven complex traits in 15 gene clusters. Shown are q-values from MAGMA gene set test. **(D)** Pseudo-time OCR peak activity (left) and gene expression (right) for OCR peak-gene pairs in npglut. The most variable features are labeled. **(E)** ASD-related GRN that consists of 4 selected TFs known to be ASD risk genes and their 42 ASD risk gene targets. Arrow colors, cell type in which the associations were identified. **(F)** The 21 TFs with targets enriched for ASD risk genes across cell types. OR, odds ratios.

To systematically characterize neuron activity-dependent transcriptional dynamics, we performed clustering analysis to group 5,221 highly variable DEGs (FC>2) with similar expression patterns across cell types and time points into “modules”. We applied expression trajectory analysis on each cell type and obtained pseudotime of each cell, capturing the extent of cellular activation. We divided pseudotime into 100 bins, and clustered genes based on 300 expression measurements (100 bins, 3 cell types). This analysis revealed 15 clusters (Fig. S12A. Table S7). While early response clusters (e.g., C6, C13) and some later response clusters (e.g., C4, C15) tended to show similar expression dynamics in all three cell types, late response genes had more variable patterns across cell types as previously reported(23): C3 was GABA-specific and C8 was Glut-specific (Fig. 2A).

We next examined the biological function of these clusters and their relevance to neuropsychiatric traits (Fig. 2B, Table S8). Notably, the early response C6 has the most enriched GO terms (biological process) among all clusters. The terms were related to cholesterol biosynthesis and metabolism, processes important for neuronal function by serving as key component of cell membranes (42–44). A late response cluster, C3, was enriched for processes related to axon guidance and ion transport. Interestingly, the “repressive” clusters 9 and 14 were enriched for genes related to DNA repair, a process that is coupled to NPAS4-activated synaptic activity(45). Testing GWAS enrichments for NPD and neurodegenerative diseases further revealed significant or suggestive enrichments in several clusters (Fig. 2C). The early response clusters C6 and C13 showed enrichment of GWAS signals of SCZ and attention-deficit/hyperactivity disorder (ADHD). The late response clusters, C3 and C8, were enriched for GWAS signals for MDD, Parkinson’s disease (PD), neuroticism score (NS), and BMI. These results highlighted the biological relevance of these clusters to disease genetics, and the importance of cholesterol metabolism to neuronal activation.

To characterize the chromatin accessibility dynamics during neuronal activation, we first linked genes with the OCRs that likely regulate their expression. By correlating single-cell chromatin accessibility with gene expression, we were able to obtain 9,503 OCR-gene pairs in 4,227 genes in GABA cells, 8,102 OCR-gene pairs in 3,930 genes in nmglut cells, and 7,882 OCR-gene pairs in 3,552 genes in npglut cells (Fig. 2D, Fig. S12B, Table S9). To confirm these OCR-gene pairs, we performed a pan-promoter capture Micro-C in neuron co-cultures at 0, 1, and 6 hrs of KCI stimulation (Fig. S12C, Table S10) to identify promoter-interacting OCRs. We then carried out activity-by-contact (ABC) (46) analysis of the Micro-C choromatin contacts (Table S11) and our snATAC-seq data, and identified 370–377K enhancer-gene pairs in each cell type (Table S12). We found that 43–48% of the “co-activation”-based OCR-gene pairs (FDR < 0.05) overlapped with ABC enhancer-gene pairs, representing a 2.3–2.8-fold enrichment (vs. non-overlapped; Fisher’s exact test *P* < 2.2 × 10^−16^) (Fig. S12D).

We next compared the changes of gene expression and chromatin accessibility in linked OCRs for expression clusters. For most clusters, we observed concordant epigenome-transcriptome changes (Fig. S12A). However, several clusters showed notable differences. In the early response cluster C6, while gene expression dropped at 6 hrs, nearby chromatin regions remained largely open, suggesting a form of “epigenetic memory” (Fig. S12A)(23). In the late response clusters C8 and C15, while gene expression changes peaked at 6 hrs (for C8, only in Glut cells), chromatin activation occurred at 1 hr, suggesting a form of “transcriptional delay” or “chromatin priming” (Fig. S12A). These epigenome-transcriptome “discordances” expand our observation earlier using a small set of known ERGs and LRGs (Fig. 1J), adding to the growing picture of the complexity of epigenome regulation, e.g., epigenetic priming during cellular differentiation and responses(47, 48).

To mechanistically understand the complex pattern of epigenome regulation of neuron activation, we leveraged our multiomic data to identify putative TF regulators of early and late response. These regulators were defined based on their motif enrichment patterns and differential expression during neuronal activation (See Supplementary text, Table S13). We identified 145 candidate TF regulators of early response, half of which are shared by all cell types (Fig. S13A). While the expression of the shared TFs often elevated transiently at 1 hr, their motifs remained enriched at 6 hrs (e.g., FOS, JUNB, and NPAS4) (Fig. S13B, D), suggesting that the epigenomic changes established by these early response TFs were maintained at a later stage. Late response TF regulators showed a very different pattern. Of the 64 candidate late response TFs, only 6 (e.g., MEF2C) were shared across cell types (Fig. S13B-D). Interestingly, some GABA-specific TFs with highest motif enrichment at 6 hrs, including ID3, DLX5, and TCF4 (a master regulator in SCZ(49)), showed high expression and strong motif enrichment even before stimulation (Fig. S13B,D), implying that the transcriptome and epigenome of GABA cells at the resting state were primed for a distinct late response.

Altogether, these results highlight a complex expression and chromatin dynamics during neuronal activation. Notably, while early responses tend to have a shared regulatory program across cell types, TF regulators of late responses varied in Glut and in GABA cells.

### Gene regulatory network inference of neuronal activation sheds light on ASD genetics

Leveraging our sn-Multiomics data, we reconstructed gene regulatory networks (GRNs) that modulate neural transcriptional response (Methods)(50). Briefly, for each gene showing differential expression in at least one condition, we defined candidate TF regulators, based on the presence of their motifs in the OCRs linked to that gene (Fig. 2D). Among these candidate TFs, we then correlated the motif activity of these TFs with the target genes’ expression across pseudotime, one cell type at a time. Our GRN inference resulted in 198 TFs, each having 100 or more targets in at least one cell type; the top TFs included well-known early response TFs, such as FOS and JUNB (Table S14).

These GRNs provide a framework to understand the functions of TFs in neuronal response and disease relevance. We illustrated the use of GRN in studying genetic regulation of ASD that has many known risk genes (n=185), including 8 TFs(51). We focused on the four TFs that likely played a role in neuronal response (Table S13): MEF2C, an early and late response TF in all cell types; MLXIP, an early response TF in all cell types; RORB, an early response TF in GABA and late response TF in npglut; TCF4, a late response TF in GABA and also a possible master regulator in SCZ(49). These four TFs regulate 298 to 1,231 genes across 3 cell types (Table S15). GO enrichment analysis of their target genes revealed some biological processes relevant to ASD, such as synaptic transmission and neuron migration (Table S16). In the case of RORB, the enriched GO processes include “lipid droplet (LD) formation” and “peptide metabolic process” (Table S16). These results suggest convergent as well as distinct processes regulated by these TFs.

To further explore the functional relevance of these TFs, we created an “ASD subnetwork” consisting of the 4 TFs and 42 ASD risk genes that were targets of at least one of the TFs (Fig. 2E). This network highlighted extensive cross-regulation of ASD risk genes by the four TFs in a cell-type-specific manner. MEF2C, for example, regulated 26 ASD genes, some of which were co-regulated by three other TFs. For instance, UBR1, a ubiquitination gene, was regulated by three TFs (MEF2C, TCF4, MLXIP) (Fig. 2E). Indeed, the shared target genes (not limiting to known ASD risk genes) of the 4 TFs were enriched for biological processes that included ubiquitin conjugating enzyme activity (Table S17), highlighting the importance of ubiquitin function in ASD.

Lastly, to infer additional TFs important for ASD but not as known ASD genes themselves, we tested each TF’s targets for the enrichment of ASD risk genes. This analysis identified 142 TFs (FDR < 0.05) across three cell types (Table S18). We highlight here the results of top 10 TFs per cell type (21 distinct TFs) (Fig. 2F). This list included several important early response TFs such as FOSL1/2, JUNB, and BACH2. The TFs showing highest enrichments for ASD genes included SREBF2, CTCF, and ZNF384 (Table S18). Notably, SREBF2 (Sterol regulatory element-binding protein 2) is important for regulating lipid and cholesterol synthesis(52, 53). Together with the enrichment of GO term “LD formation” among targets of RORB, an ASD risk gene (Fig. 2E), these results supported a possible link between lipid/cholesterol-related processes and ASD.

Altogether, these results provide mechanistic insight on neuron activity-dependent regulation of ASD risk genes, highlighting the role of some TFs as key regulators governing ASD gene networks.

### Expression QTL mapping reveals stimulation-dependent effects of genetic variants on expression

**Fig. 3.**
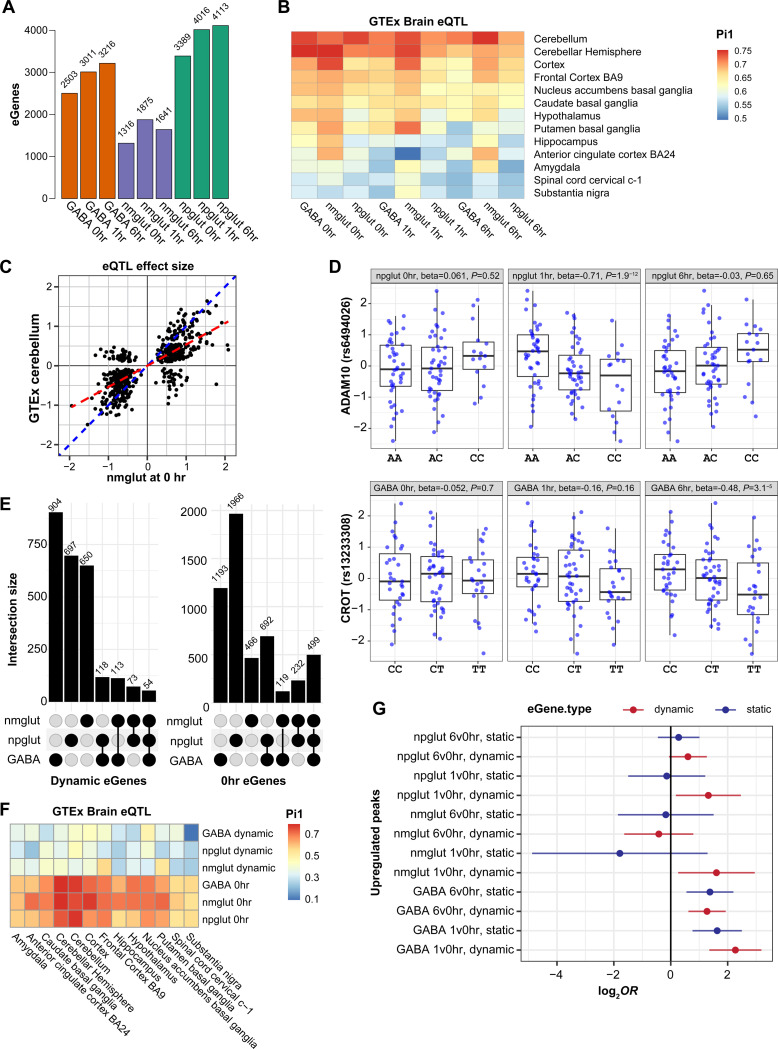
eQTL mapping identified neuronal stimulation-dependent effects of genetic variants on expression. **(A)** The number of eGenes of each context (cell type × time point). **(B)** Proportion of neuronal activity eQTL significant in GTEx brain tissues. **(C)** Effect size concordance of eQTL between 0-hr nmglut and GTEx cerebellum. Each dot, an eQTL. Red line, fitted line with intercept=0; blue line, slope=1. **(D)** Examples of dynamic eQTL. eQTL of ADAM10 (top panel) is only significant in npglut at 1 hr. eQTL of CROT (bottom panel) is only significant in GABA at 6 hrs. **(E)** The number of dynamic eGenes (left) and those in unstimulated neurons (0 hr) (right) across cell types. **(F)** Proportion of shared neuron activity eQTL in GTEx eQTL by Pi1 analysis. Top 3, eGenes from dynamic test; bottom 3, eGenes from neurons at 0 hr. **(G)** Enrichment of upregulated OCR peaks in dynamic eQTL and static eQTL.

Genetic variations associated with neuron activity-dependent expression are unknown. With a relatively large cohort of iPSC lines, we mapped eQTL for each of the 9 “contexts” (3 cell types, 3 time points). We identified 1,316–4,113 genes with at least one eQTL (eGenes) across contexts (Fig. 3A, Table S19). The numbers of eGenes from stimulated conditions were generally larger than those from 0 hour (Fig. 3A). Indeed, large fractions of eQTL were mapped only in stimulated states (Fig. S14A-C), highlighting the advantage of using stimulation to reveal genetic effects that would otherwise be missed(54).

We compared our eQTLs to GTEx brain eQTL(55). Pi1 analysis showed stronger sharing (>0.65) with GTEx data for cerebellum, cerebellar hemisphere, and cortex than other brain regions, with strongest sharing found with 0 hr eGenes (Fig. 3B). This suggested neuronal stimulation allows detection of eGenes that may be missed in postmortem brain eQTL mapping. Overall, 19–61% of our eGenes were shared with GTEx brain eGenes (Fig. S14D). Among the shared eGenes, we observed a strong correlation of the effect sizes between the two datasets (Fig. 3C). In contrast, the effect sizes of our eQTL showed an expected lower correlation with GTEx whole blood eQTL (Fig. S14E). The proportion of GTEx brain eQTL sharing effect directions with our eQTL was substantially higher than that of GTEx blood eQTL (Fig. S14F). These results thus supported the validity of our eQTL.

To determine the “dynamic eQTL” showing different effect sizes upon neuronal stimulation, we focused on the 9,880 eGenes from our eQTL analysis combining all 9 conditions (Methods). To assess the difference of eQTL effect size between 0 hr and 1 or 6 hrs, we performed interaction testing, one cell type at a time. To increase the power of analysis, we adjusted non-genetic differences across individual cell lines using linear mixed models (LMM, see Methods). Our interaction test revealed 1189, 942, and 890 dynamic eGenes in GABA, npglut, and nmglut cells, respectively (Fig. 3D, Table S20). For comparison, we considered “static eQTL” as those eQTL at 0 hr.

Comparing eQTL across cell types, we noted that dynamic eQTL tended to be more cell-type-specific than static eQTL (Fig. 3E): 2% dynamic vs. 10% static eQTL were shared across all three cell types. We next compared the extent of overlap of our neuronal eQTL with GTEx brain eQTL. We found that while static eQTL showed considerable overlap with GTEx eQTL, much smaller proportions of dynamic eQTL were shared with GTEx (Fig. 3F). These results suggested that neuronal stimulation revealed novel eQTLs missed by brain eQTL mapping. We hypothesize that dynamic eQTL were driven by activity-dependent epigenomic changes. To test this, we assessed the enrichment of upregulated peaks between time points in the dynamic eQTL. We found that dynamic eQTL showed stronger enrichment in DA peaks than static eQTL in the corresponding cell types (Fig. 3G).

Altogether, our study revealed stimulation-dependent eQTL, which were more likely cell-type-specific, less shared with brain eQTL, and enriched with OCRs involved in neuronal responses.

### Joint analysis of eQTL and GWAS identified stimulation-specific NPD risk genes

To examine whether stimulation-dependent eQTL can help map GWAS risk genes of NPD, we used our recently developed method causal-TWAS (cTWAS)(56), a generalization of the Transcriptome-wide Association Study (TWAS) but with better control of false discoveries. cTWAS allows joint analysis of GWAS with eQTL data from multiple contexts to identify “causal expression traits”, meaning gene expression in a particular context with an effect on the phenotype. We note that for a risk gene of a trait, it likely acts on the trait only in particular trait-related contexts, thus finding the “causal contexts” would be biologically interesting. To apply cTWAS, we first created prediction models for gene expression in each context, followed by computing the Posterior Inclusion Probability (PIP) of each expression trait being causal to a phenotype. We also computed the aggregated PIPs of all expression traits of the same gene (i.e., “gene PIP”) as the probability of a gene being causal to the phenotype (Methods).

**Fig. 4.**
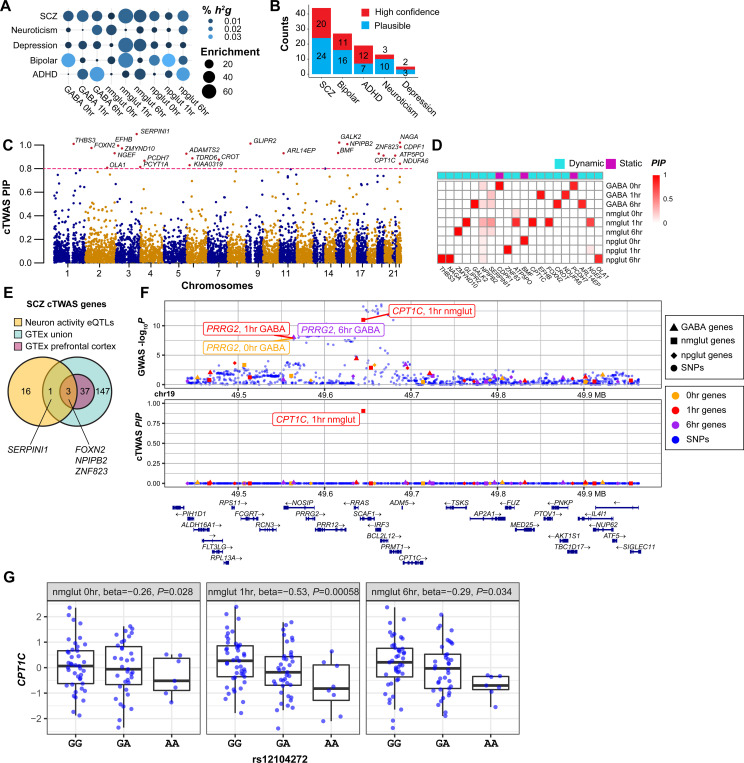
eQTL-based causal transcriptome-wide association study (cTWAS) identified putative neuronal stimulation-specific NPD risk genes. **(A)** Genetic parameters estimated by cTWAS. Enrichment, fold enrichment of causal GWAS signals in eGenes (vs. background SNPs); h^2^g, proportion of heritability mediated by eQTL. **(B)** Counts of candidate eGenes across 5 NPD phenotypes stratified by posterior inclusion probability (PIP). High confidence, PIP > 0.8; plausible, 0.5 < PIP < 0.8. **(C)** Manhattan plot for SCZ cTWAS with PIP as the y coordinates and genomic position as the x coordinates. Red dashed line, PIP threshold of 0.8. b PIPs across contexts for high confidence SCZ risk genes. A gene is “dynamic” if the sum of PIPs in stimulating states (1 or 6 hrs) is larger than the PIP at 0 hr by 0.5. **(E)** Sharing of risk genes from cTWAS between our neuronal activity eQTL and GTEx brain eQTL. **(F)** Locus plot of *CPT1C* in cTWAS. Top panel, *P* values of GWAS SNPs and gene expression traits (from standard TWAS); bottom panel, cTWAS PIPs of SNPs and gene expression traits. SNPs shown as dots, and eGenes as other shapes. **(G)** Box plot of gene expression (vs. genotype) of *CPT1C* in nmglut across time points. Only 1 hr has a strong genetic effect.

We first evaluated the enrichment of GWAS signals and the percentage of heritability explained by expression traits in each context. We found that genetically predicted expression traits across 9 contexts were broadly enriched with GWAS signals of neuropsychiatric traits (Fig. 4A), with SCZ showing the largest enrichments (20–60 folds). To quantify the overall contribution of eQTL to trait genetics, we used cTWAS to estimate percent of heritability explained by eQTL in each context (Fig. 4A). In total, eQTL across contexts explained about 5 to 14% of heritability for neuropsychiatric traits (Fig. S14G), which is in line with estimations for other traits using eQTL data(56, 57). In general, the expression traits upon stimulation showed higher GWAS enrichments than at the baseline condition. For ADHD, only stimulated contexts showed GWAS enrichments (Fig. 4A). These results highlight that the stimulation states more specifically capture disease-related cellular contexts.

We next identified putative disease causal genes (Fig. 4B). SCZ showed the largest number of causal genes (Table S21), with 44 plausible ones at PIP > 0.5 (Fig. 4B-C). Focusing on the 20 high confidence (PIP > 0.8) SCZ genes (Fig. 4C), we found that cTWAS often identified the likely causal contexts for these genes with larger PIPs (Fig. 4D). Notably, these causal contexts were often the stimulated states (Fig. 4D). To better quantify this trend, we classified a cTWAS gene as “dynamic” if the total PIP from the stimulated states (1 and 6 hrs) was greater than the PIP from 0 hr by at least 0.5, and as “static” otherwise. Using this criterion, we found the majority of cTWAS genes for SCZ were “dynamic” (Fig. 4C-D, Fig. S14H).

To assess the novelty of our finding, we compared our cTWAS results for SCZ to those from using the GTEx brain eQTL. At PIP > 0.8, we found only 3 shared genes (*FOXN2*, *NPIPB2*, *ZNF823*) with cTWAS results from prefrontal cortex (Fig. 4E). Notably, both *FOXN2* and *ZNF823* are credible SCZ risk genes(41, 58, 59). Even with cTWAS genes from all GTEx brain tissues, only one more gene (*SERPINI1*) were shared (Fig. 4E; Fig. S14I). These results highlight the utility of stimulation-driven eQTL in discovering novel risk genes.

To understand the biological relevance of the identified cTWAS genes, we performed GO enrichment analysis on the union of candidates (PIP > 0.8) for three related NPDs: SCZ, BP, and MDD. The top GO terms included “amino acid betaine metabolic processes”, “peripheral nervous systems development”, and “mitochondrial electron transport chain” (Table S22). The amino acid metabolic processes term (q = 0.01) was driven by two genes for SCZ, *CROT* (PIP = 0.87) and *CPT1C* (PIP = 0.91), with the latter driven by its eQTL at 1 hr of stimulated nmglut cells (Fig. 4F). Indeed, the eQTL driving the cTWAS results showed larger effects at 1 hr vs. other time points (Fig. 4G). Interestingly, *CPT1C* plays an important role in hypothalamic lipid metabolism(60, 61). These results are reminiscent of our earlier findings that suggested lipid metabolism as a key process activated during neuronal stimulation (Fig. 2B, Cluster 6) and in ASD risk gene regulation (i.e., RORB targets, Table S16), further implying possible dysregulation of lipid metabolism in NPD.

### Chromatin QTL mapping uncovers abundant neuronal activation-dependent regulatory variants

**Fig. 5.**
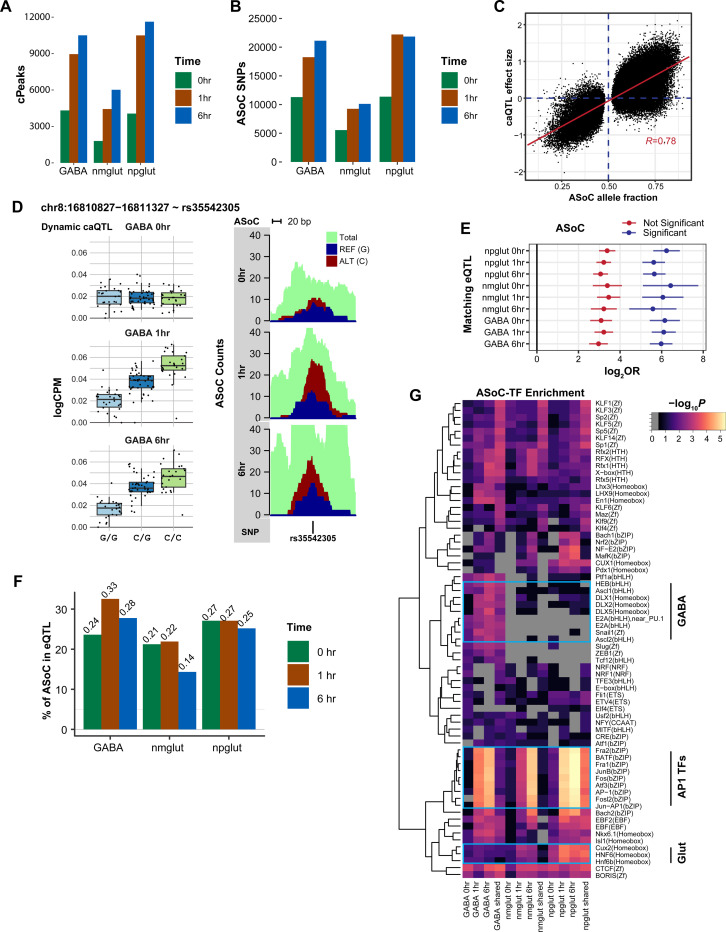
caQTL mapping uncovered neuronal stimulation-specific regulatory variants. **(A)** Counts of cPeaks (caQTL peaks) by context (cell type x time point). bCounts of ASoC SNPs by context. **(C)** Comparison of ASoC allele fractions and caQTL effect sizes across nine contexts. Red line, fitted slope; Pearson’s R=0.78. **(D)** A dynamic caQTL (rs35542305) for chromatin accessibility at chr8:16810827−16811327 in GABA (left) shows consistent allelic imbalance (i.e., ASoC, right). Each dot (left panels), a cell line; CPM, count per million sequencing reads. **(E)** Fold enrichment of ASoC in eQTL in each matched context using TORUS analysis. FDR < 5% as a cutoff for significant ASoC. **(F)** Non-null proportion (Pi1) of ASoC SNPs in eQTL from the match context. **(G)** Heatmap shows the enrichment of TF motifs in ASoC SNP-flanking sequences (+/− 25 bp) at each context.

Chromatin accessibility of *cis-*regulatory sequences controls gene expression and is influenced by individual genetic variation. We next carried out chromatin accessibility QTL (caQTL) mapping to identify genetic variants associated with chromatin accessibility for each context (cell type, time point). We found 1.8~11.6K peaks with at least one caQTL (cPeaks) in *cis* within 25 kb of a SNP (Fig. 5A, Table S23). Using a statistical interaction test, we found that 5,461 to 9,832 caQTL were dynamic, showing different effect sizes in 1 or 6 hrs than 0 hr (Table S25). We identified substantially more (>2-fold) cPeaks at 1 hr and 6 hrs of stimulation than at 0 hr (Fig. 5A). As another way of mapping genetic variants with effects on chromatin accessibility, we performed allele imbalance analysis of heterozygous variants as described(62, 63). This analysis revealed 5.6~21.9K allele-specific open chromatin (ASoC) variants across 9 contexts, again with substantially more ASoC variants under stimulation (Fig. 5B; Fig. S15A-C; Table S24). Because ASoC analysis, using intra-individual variations, was based on different statistical signals from caQTL, we assessed the agreement between the two analyses. We observed highly correlated effect sizes between the two (Pearson R = 0.78) (Fig. 5C), and top dynamic caQTL often showed strong stimulation-specific ASoC (Fig. 5D, Fig. S15D, Table S25), supporting the consistency of the two analyses. These results highlight the dynamic nature of variant effects on chromatin accessibility.

We hypothesized that caQTL variants likely influence gene expression. To test this, we assessed the enrichment of caQTL variants (for simplicity, we used ASoC variants here) in eQTL. We found strong enrichment (30–80 fold) of ASoC variants in eQTL of the matching context (Fig. 5E). We also estimated the proportion of likely eQTL in ASoC variants by using pi1 analysis. We found that 15–30% of ASoC variants were likely eQTL in the matching contexts (Fig. 5F). Further comparison to brain eQTL showed that ASoC SNPs were strongly enriched for frontal cortex eQTL in both GTEx and PsychoENCODE datasets(64, 65) (Fig. S16A-C), with 0 hr ASoC SNPs showing the largest overlap with brain eQTL. These results support that caQTL variants likely affect gene expression, but a large fraction of caQTL variants may be missed by eQTL mapping, thus providing a complementary way of uncovering regulatory variants.

To assign putative *cis*-target genes of ASoC SNPs, we examined whether an ASoC SNP was located inside a promoter or a promoter-interacting OCR in our Micro-C dataset (Fig. S12D, Table S10). With the 79–89K interacting bins across time points (Table S11), we found 24–44% of ASoC SNPs could be assigned to one or more target genes (Fig. S16D, Table S26). We next examined whether stimulation-specific AsoC SNPs were more likely in enhancers or promoters, and whether they were enriched for specific TF binding sites. Using GREAT(66), we found that stimulation-specific ASoC (vs. static ones) were more enriched in enhancers (vs. promoters) (Fig. S17A-C). To identify specific TFs that might drive ASoC in each context, we examined the TF-binding motif enrichment at ASoC SNP sites. We found cell-type-specific TF enrichment, e.g., ASCL1 and DLX1/2/5 for GABA neurons and CUX2 for Glut neurons (Fig. 5G). Notably, the enriched TFs could clearly distinguish stimulation phase: early response TFs such as FOS and JUNB were strongly enriched in ASoC SNPs at 1 hr and 6 hrs but not in ASoC at 0 hr (Fig. 5G). These results suggest that cell-type- and activation-specific TF binding in enhancers/promoters may drive context-specific caQTL.

### Activity-dependent caQTL partly explain heritability of NPD and other brain traits

**Fig. 6.**
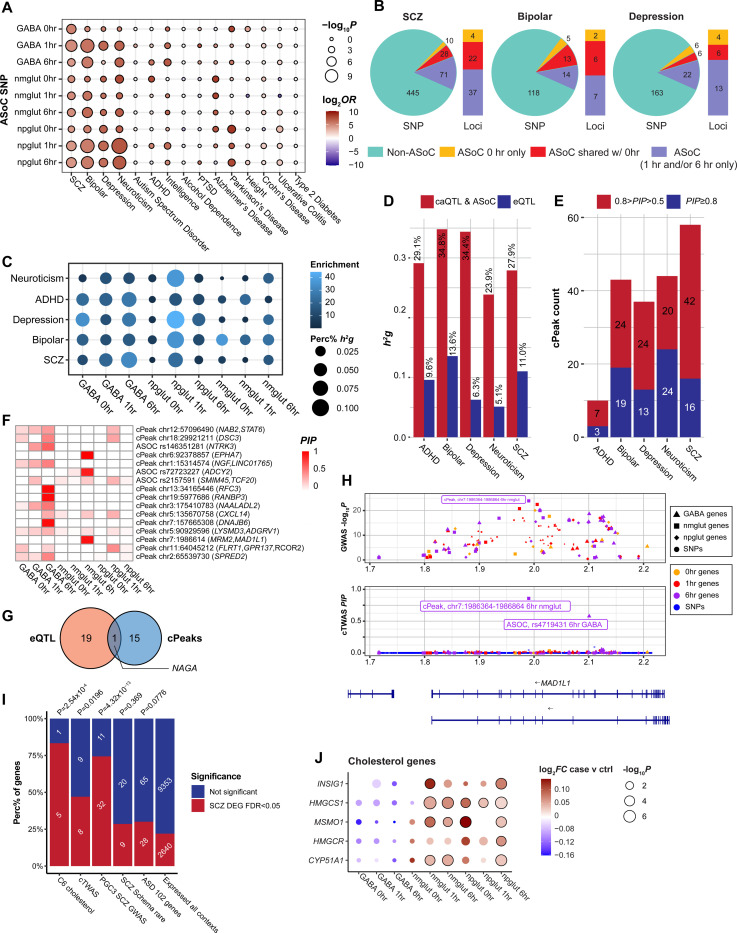
Activity-dependent caQTL explain genetic risk of NPD. **(A)** TORUS analysis of GWAS enrichment for ASoC SNPs of each context (vs. background SNPs). **(B)** Number of GWAS index SNPs and their LD proxies (R^2^>0.8) that also show ASoC (or not) for SCZ, BP, and MDD. The vertical bars show the number of putative functional GWAS risk loci that can be potentially explained by ASoC at 0 hr or upon stimulation. **(C)** GWAS enrichment and percentage of SNP heritability (h^2^g) of caQTL (including ASoC) in cTWAS in each context for major NPD. **(D)** Comparison of h^2^g explained by caQTL and eQTL for each phenotype. **(E)** Causal cPeak counts from cTWAS for each phenotype, stratified by PIPs. **(F)** Distribution of the PIPs of high confidence cPeaks across contexts. Target genes of a cPeak are defined as the nearest gene (TSS) and/or eGene for the caQTL SNPs. **(G)** Venn diagram showing the overlap between high-confidence cTWAS eGenes and cPeaks. Overlap is defined by eQTL SNPs within 500 kb range of the caQTL SNPs. **(H)** Locus plot of cTWAS cPeak (chr7:1986364–1986864) in *MAD1L1*. Top panel, *P* values of GWAS SNPs and peak accessibility traits (from standard TWAS); bottom panel, cTWAS PIPs of SNPs and cPeaks. SNPs: dots, cPeaks: other shapes. **(I)** Gene set enrichment (vs. expressed in all contexts) for SCZ-associated DEGs of all contexts. Fisher’s exact test. **(J)** Cholesterol genes (in module C6) showed SCZ-associated DE (FDR<0.05, circled). Bubble plot shows the log_2_FC and −log_10_*P* of DE from MAST test.

To assess the role of neuronal activity-dependent chromatin accessibility variants in NPD genetics, we started with a TORUS analysis of GWAS risk enrichment for ASoC variants of each context. For each cell type, ASoC SNPs at 1 hr or 6 hrs of stimulation generally showed stronger enrichment (for SCZ, bipolar, depression, and neuroticism) than ASoC SNPs of unstimulated neurons (Fig. 6A), suggesting stimulation helps unravel functional risk variants. To assess to what extent neuron stimulation can help prioritize functional GWAS risk variants for major NPD (SCZ(41), BP(67), and MDD(68)), we intersected ASoC SNPs to GWAS of NPDs and estimated the number of disease loci whose lead SNPs or LD proxies (r^2^>0.8) overlapped with at least one ASoC SNPs. We found that stimulation substantially increased the number of GWAS risk loci that have GWAS risk SNPs overlapping with ASoC SNPs (from 26 to 63 for SCZ, 18 to 32 for BP, and 10 to 23 for MDD) (Fig. 6B, Tables S27-29). For the ASoC SNPs that might be the functional GWAS risk variants, many (73% for SCZ, 66% for BP, and 50% for MDD) could be assigned to a Micro-C *cis*-target gene (Tables S27-29). Thus, our caQTL mapping, especially the activity-dependent ASoC, substantially increased the putatively functional GWAS risk variants of NPD.

We then extended our cTWAS analysis on all variants associated with chromatin accessibility, including caQTL and ASoC variants. We treated cPeaks as our analysis unit and assessed the contribution of the genetic components of these cPeaks to NPD. We took the union of cPeaks with either a caQTL or ASoC variant, resulting in 15–38K cPeaks across contexts (Table S30). We then used the top variant (caQTL or ASoC, based on *p*-values) for each cPeak as the prediction model of that peak in cTWAS analysis. We found that cPeaks at all contexts were broadly enriched with genetic signals from GWAS of major NPD (Fig. 6C). The enrichments and the proportions of heritability explained by cPeaks were generally higher in stimulated neurons than those at 0 hr (Fig. 6C). In total, cPeaks explained considerably larger proportions of SCZ heritability than eQTL (28% for cPeaks vs. 11% for eQTL) (Fig. 6D). Even after we controlled for eQTL in cTWAS analysis (see Methods), the proportion of heritability explained by cPeaks was only modestly reduced (28% to 25%) (Fig. S18A). These results highlighted the important contributions of genetic variants acting on epigenomes during neuronal activities to NPD.

cTWAS identified 3–24 high-confidence (PIP > 0.8) causal cPeaks across NPD (Table S30), with 16 for SCZ. A relaxed PIP (> 0.5) gave a larger number of cPeaks, including 58 cPeaks for SCZ (Fig. 6E). To identify the contexts driving these results, we partitioned the PIPs of SCZ cPeaks across 9 contexts. In most cases, PIPs were contributed by stimulated conditions and often in single cell types (Fig. 6F, Fig. S18B,C).

We next compared the caQTL-based cTWAS results for SCZ with earlier results using eQTL. For comparison, we considered the results from the two analyses “shared” if a cPeak and the TSS of an eGene were within 500 kb. We found only 1 (ASoC_rs2157591) out of 16 cPeaks at PIP > 0.8 (8 out of 58 with PIP > 0.5) were shared with the results from eQTL (*NAGA*, a SCZ risk gene that encodes lysosomal enzyme alpha-N-acetylgalactosaminidase that regulates dendritic maturation(69)) (Fig. 6G, Fig. S18D), highlighting the benefit of using caQTL to identify new causal genes.

Lastly, we examined the functional relevance of the cPeaks. We linked cPeaks to their putative target genes through several means: co-activation of peaks with gene expression, eQTL of the caQTL or ASoC variants, genes overlapping with the cPeaks, and ABC scores computed from ATAC and Micro-C data. Focusing on the potential target genes of eQTL (at FDR<0.2) for SCZ, we found 6 out of 16 cPeaks (PIP > 0.8) could be linked to at least one target gene, with five to a unique gene (Table S30). Except for one lncRNA gene, the other four unique genes are all plausible SCZ candidate genes (Fig. 6H, Fig. S18E). *MAD1L1* is a known SCZ risk gene(41) and plays a role in neurogenesis(70). The cTWAS result for MAD1L1 was largely driven by a single cPeak controlled by a caQTL of nmglut cells at 6 hrs (Fig. 6H). *STAT6* is important for neuroinflammation, learning and memory(71, 72). *TCF20* is linked to developmental disorders, playing a role in brain development and function(73). *ADGRV1* is a risk gene for several nervous system disorders (e.g., hearing loss, blindness, epilepsy)(74–76).

Altogether, cTWAS results using caQTL highlighted the power of our multiomics QTL mapping in stimulated neurons, nominating many candidate regulatory sequences and genes for future studies.

### Some SCZ risk genes and cholesterol metabolism genes are differentially activated in SCZ patient-specific neurons

Leveraging our sizable SCZ iPSC cohort (n=28), we examined whether the SCZ-relevant gene sets highlighted above (i.e.,SCZ GWAS risk genes, cTWAS genes, or cholesterol metabolic genes in C6 cluster) show differential neuronal activation in SCZ cases. We carried out single-cell DEG analysis in iNs between sex/age-matched SCZ cases and controls (Fig. S19A, Table S1). We identified 753 to 1753 SCZ-associated DEGs (FDR < 0.05) in each cell type, of which 59–61% are activity-dependent (Fig. S19B-D, Table S31). GO-term enrichment analysis showed that activity-dependent DEGs were strongly enriched in GO-terms related to axon guidance, axonogenesis, and synaptic transmission (Fig. S19B-D). Notably, cholesterol biosynthetic process is among the most enriched GO-terms in npglut (Fig. S19D), which is consistent with our observed enrichment of cholesterol genes in the SCZ-relevant C6 module (Fig. 2B,C). Indeed, 5/6 cholesterol genes in the C6 module exhibited SCZ-associated DE, representing a 3.8-fold enrichment (Fig. 6I,J). For cTWAS SCZ genes (caQTL-based, Fig. 6F) and SCZ GWAS genes (prioritized single genes, Table S5), we also observed significant enrichments (2.1 and 3.4, respectively) of SCZ-associated DEGs (Fig. 6I, Fig. S19E-F). In contrast, SCZ or ASD risk genes from rare variant analysis (Table S5) did not show enrichment of SCZ-associated DEGs. Moreover, many genes only showed SCZ-associated DE upon neuronal activation (Fig. S19E-I), especially the cholesterol genes (Fig.6J), highlighting the importance of context-specific regulation of disease risk genes.

## Discussion

Compared to previous studies(25, 26, 32, 33), our single-neuron multiomics study in a large cohort of human donors uniquely characterized the individual variation of neuronal response to KCI stimulation and its genetic control. We identified thousands of genetic variants associated with individual variations of neuronal responses (i.e., eQTL and caQTL including ASoC variants). We found that neuronal stimulation substantially increased the number of eQTL (or eGenes) and caQTL (or cPeaks). Compared to the QTL found at the resting state, these stimulation-specific QTL tend to be shared less with post-mortem brain eQTL and more enriched for NPD risk, highlighting the power of neuronal stimulation to reveal genetic effects that would otherwise be missed.

Our study systematically demonstrated epigenomic discordance between gene expression and chromatin accessibility (i.e., chromatin priming or transcriptional delay)(23) in single neurons upon activation. Moreover, our large cohort enabled us to characterize genetic variation of neuron activity-dependent gene expression and chromatin activity, and to show their relevance to NPD. We found that eQTL and eGenes upon stimulation showed stronger enrichments of NPD GWAS risk genes than baseline conditions (Fig. 4A), and most candidate genes were identified under the stimulation contexts (Fig. 4D, Fig. S14E). In support of the eQTL results, ASoC SNPs and cPeaks showed stronger enrichments for NPD GWAS risk genes upon stimulation (Fig. 6A,C), and caQTL-based cTWAS identified more “causal” cPeaks under stimulation contexts (Fig. 6F, Fig. S18B-C). These results suggest that many NPD risk variants/genes may only manifest functional effects upon neuronal stimulation, highlighting the importance of mapping genetic variants that regulate neuron activity-dependent epigenomes.

It is noteworthy that caQTL (including ASoC) explained considerably larger proportions of NPD heritability than eQTL (28% from cPeaks vs. 11% from eQTL for SCZ) (Fig. 6D). There are two possible explanations. First, regulation of gene expression is considerably more complex than individual regulatory elements, involving multiple enhancers that affect transcription and other elements regulating RNA stability. Thus, the effect of a genetic variant on expression is likely smaller than on chromatin accessibility, making it harder to identify eQTL than caQTL. Second, as we observed, epigenomic and transcriptomic changes are not always synchronized, e.g., regulatory sequences of ERG may remain open even after gene expression has restored to the resting state. This makes it possible to identify caQTL effects in the absence of eQTL effects. Regardless of the exact explanation, our observation that caQTL explain more heritability of complex traits has broad implications on genetic studies: caQTL mapping, especially in a cellular stimulation-context, may be more efficient than eQTL mapping for unraveling the effects of risk variants (77, 78).

Our study shed new mechanistic insights onto the regulatory mechanisms of cell-type-specific neuronal response. TFs regulating neuronal early responses tend to be shared by all cell types. Interestingly, some shared TFs (e.g., *FOS, JUNB, NPAS4*) showed peak expression at 1 hr while their motifs remain enriched at 6 hrs (Fig. S13B). In contrast, TFs regulating late responses tend to be cell-type-specific, especially in GABA cells where some TFs (e.g., *TCF4, ID3, DLX5*) showed high expression and strong motif enrichment even before stimulation (Fig. S13B,D). These results suggest that both shared and cell-type-specific TFs work together, possibly through regulatory cascades, to create cell-type-specific neuronal responses.

An interesting finding is that multiple analyses support a role of activity-dependent lipid/cholesterol metabolism in NPD. The early response gene cluster, C6, which showed GWAS enrichment for SCZ, was also enriched for cholesterol metabolic genes (Fig. 2B-C). Remarkably, 5/6 cholesterol genes in C6 showed DE in Glut iNs from SCZ donors upon activation (Fig.8J). Moreover, SREBF2, a TF with targets highly enriched for ASD genes (Fig. 2F), regulates lipid and cholesterol synthesis(52, 53). We also observed enrichment of the GO term “lipid droplets formation” among targets of RORB, an early response AD risk TF (Fig. 2E). Finally, CPT1C, a SCZ causal cTWAS gene identified in stimulated neurons regulates fatty acids transport into mitochondria for beta-oxidation(60). These results added additional support for a possible link between dysregulation of lipid/cholesterol-related processes and NPD(44).

In summary, our work provides novel mechanistic insights on neuron subtype-specific activity-dependent gene expression and substantially expands the repertoire of context-specific causal variants/genes for NPD and other brain traits, providing a rich resource for future studies.

## Supplementary Material

Supplement 1

Supplement 2

Supplement 3

Supplement 4

Supplement 5

Supplement 6

Supplement 7

Supplement 8

Supplement 9

Supplement 10

Supplement 11

Supplement 12

Supplement 13

Supplement 14

Supplement 15

Supplement 16

Supplement 17

Supplement 18

Supplement 19

Supplement 20

Supplement 21

Supplement 22

Supplement 23

Supplement 24

Supplement 25

Supplement 26

Supplement 27

Supplement 28

Supplement 29

Supplement 30

Supplement 31

Supplement 32

Supplement 33

Supplement 34

## Data Availability

The snRNA/ATAC-seq raw data, processed count matrices and fragment files, and VCF genotype files for samples demultiplex are accessible at Gene Expression Omnibus under accession code PRJNA1194194 and GSE286488. All codes used in the analyses are accessible at https://zenodo.org/records/14577991
