# Supplementary material for: Single-cell multiomics of neuronal activation reveals context-dependent genetic control of brain disorders
Source: bioRxiv. 2025 Feb 17:2025.02.17.638682. Preprint. [Version 1] doi: 10.1101/2025.02.17.638682 (PMC11870544; doi:10.1101/2025.02.17.638682)
Supplement: Supplement 1 [file media-1.docx]

Supplementary Materials for

**Single-cell multiomics of neuronal activation reveals context-dependent genetic control of brain disorders**

Lifan Liang^1,†^, Siwei Zhang^2,5,†^, Zicheng Wang^1,†^, Hanwen Zhang^2,†^, Chuxuan Li^2,3,†^, Alexandra C. Duhe^2^, Xiaotong Sun^1^, Xiaoyuan Zhong^1^, Alena Kozlova^2^, Brendan Jamison^1,2^, Whitney Wood^2^, Zhiping P. Pang^4^, Alan R. Sanders^2,5^, Xin He^1,‡^, Jubao Duan^2,5,‡^

‡ Corresponding authors: Xin He ([xinhe@uchicago.edu](mailto:xinhe@uchicago.edu)), Jubao Duan ([jduan@uchicago.edu](mailto:jduan@uchicago.edu))

The PDF file includes:

Table of Contents

Materials and Methods

Supplementary Text

Figs. S1 to S19

Tables S1 to S33

Reference (79-119)

Table of Contents

[Materials and Methods 4](#_Toc189155015)

[Human iPSC lines and cell culture 4](#_Toc189155016)

[Lentivirus preparation 4](#_Toc189155017)

[Neuronal differentiation and co-culture 4](#_Toc189155018)

[KCI stimulation of neuronal cultures 5](#_Toc189155019)

[Single nuclei multiomics sequencing library preparation 6](#_Toc189155020)

[Immunocytochemistry 6](#_Toc189155021)

[BDNF OCR peak deletion by CRISPR/Cas9 editing 6](#_Toc189155022)

[RNA isolation and qPCR 7](#_Toc189155023)

[Multiomics sequencing and data quality control (QC) 7](#_Toc189155024)

[Multiomic data analyses 8](#_Toc189155025)

[Barcode level identification of cell line identity 8](#_Toc189155026)

[snRNA-seq data analyses 8](#_Toc189155027)

[snATAC-seq data analyses 8](#_Toc189155028)

[Approximation of developmental stages for iPSC-derived neurons 9](#_Toc189155029)

[PCA analysis 9](#_Toc189155030)

[Pseudo-bulk RNA-seq differential gene expression analysis 9](#_Toc189155031)

[MAGMA gene set enrichment analysis 10](#_Toc189155032)

[Gene ontology enrichment analysis 10](#_Toc189155033)

[Differential peak accessibility analysis 10](#_Toc189155034)

[Stratified linkage disequilibrium score regression (sLDSC) for GWAS enrichment analysis 11](#_Toc189155035)

[Integration of snATAC-Seq Profiles and Peak Calling for caQTL and gene regulatory network analyses 11](#_Toc189155036)

[Single-cell Embeddings 12](#_Toc189155037)

[Inference of Pseudo-time 12](#_Toc189155038)

[Co-activation analysis to link OCRs to genes 12](#_Toc189155039)

[Activity-by-Contact (ABC) analysis 13](#_Toc189155040)

[Gene Module Analysis 13](#_Toc189155041)

[Defining candidate TFs regulating early and late responses 14](#_Toc189155042)

[Gene Regulatory Network Analysis 14](#_Toc189155043)

[eQTL mapping 14](#_Toc189155044)

[Dynamic eQTL testing 15](#_Toc189155045)

[TORUS enrichment analyses of eQTLs and ASoC variants 16](#_Toc189155046)

[Analysis of genetic effect sharing between neuronal activity eQTL and GTEx and ASoC. 17](#_Toc189155047)

[caQTL Mapping 17](#_Toc189155048)

[ASoC mapping 18](#_Toc189155049)

[Homer enrichment analysis of TF-binding motifs for ASoC SNPs 19](#_Toc189155050)

[Brain eQTL enrichment analysis for ASoC SNPs 19](#_Toc189155051)

[Micro-C analysis of ASoC SNPs and their targets 20](#_Toc189155052)

[Promoter and enhancer enrichment analysis for ASoC SNPs 20](#_Toc189155053)

[Integrative analysis of NPD GWAS with neuronal activity eQTL or caQTL 22](#_Toc189155054)

[Single-cell differential gene expression analysis in SCZ neurons 24](#_Toc189155055)

[Statistical analyses 24](#_Toc189155056)

[Supplemental Text 26](#_Toc189155057)

[Sn-Multiomics data QC and preliminary analysis 26](#_Toc189155058)

[Transcriptomic and epigenomic landscape of cell-type-specific neuronal activation and its relevance to NPD 26](#_Toc189155059)

[Activity-dependent expression of BDNF is regulated by cell-type-specific OCRs 28](#_Toc189155060)

[Integrative multiomic analysis identifies regulatory program of neuronal activation 28](#_Toc189155061)

[Supplemental Figures 31](#_Toc189155062)

[Fig. S1. Multiomics data processing and quality control (QC) by batch. 33](#_Toc189155063)

[Fig. S2: QC metrics of snATAC-seq. 34](#_Toc189155064)

[Fig. S3: Integrative analyses of snRNA-seq and snATAC-seq for all 100 lines. 36](#_Toc189155065)

[Fig. S4: Additional QC metrics on the merged snRNA-seq library. 38](#_Toc189155066)

[Fig. S5. Projection of iPSC-derived iGlut and GABA neurons to brain excitatory and inhibitory neurons of various developmental stages (Velmeshev 2023). 39](#_Toc189155067)

[Fig. S6. Cell compositions and known early or late response gene expression across three time points. 41](#_Toc189155068)

[Fig. S7. Differentially expressed genes (DEGs) upon neuronal stimulation. 43](#_Toc189155069)

[Fig. S8. Biological relevance of neuron activity-dependent DEGs. 45](#_Toc189155070)

[Fig. S9. GWAS enrichment of differentially expressed gene and the mapping of neuronal activity-dependent OCR peak. 47](#_Toc189155071)

[Fig. S10. Differentially accessible (DA) peak analysis. 49](#_Toc189155072)

[Fig. S11. DA peaks of BDNF. 50](#_Toc189155073)

[Fig. S12. Neuron activity-dependent gene expression modules (clusters) and correlation between chromatin accessibility and gene expression for OCR-gene pairs. 53](#_Toc189155074)

[Fig. S13. TF regulation of early and late neuronal response and ASD-related gene regulatory network (GRN). 54](#_Toc189155075)

[Fig. S14. Neuron activation-dependent eQTL mapping and characterization. 55](#_Toc189155076)

[Fig. S15. caQTL (including ASoC) mapping. 58](#_Toc189155077)

[Fig S16. Enrichment of ASoC SNPs (vs. non-ASoC control SNPs) in brain eQTL and comparison with Micro-C chromatin contact. 59](#_Toc189155078)

[Fig. S17. Enrichment of ASoC SNPs in different regulatory sequence elements. 61](#_Toc189155079)

[Fig. S18. Integrative analysis of caQTL and GWAS of NPD phenotypes by cTWAS. 62](#_Toc189155080)

[Fig. S19. Differential expressed genes (DEGs) in neurons from SCZ cases vs. controls. 65](#_Toc189155081)

[List of Supplemental Tables 66](#_Toc189155082)

[References Only Cited in Supplemental Materials 68](#_Toc189155083)

# Materials and Methods

## Human iPSC lines and cell culture

We initially started with 107 iPSC lines (European ancestry) of which 100 lines were successfully differentiated into both excitatory and inhibitory neurons (Table S1). Of the 100 iPSC lines used for data production, 58 with their IDs starting with “CD” were reprogrammed at Rutgers University Cell and DNA Repository (RUCDR)-NIMH Stem Cell Center using the cryopreserved lymphocytes (CPLs) of donors of Molecular Genetics of Schizophrenia (MGS) cohort, and have been used in previous studies(*62, 63, 79-81*). The rest were purchased from the California Institute of Regenerative Medicine (CIRM). 28 iPSC lines are from SCZ cases and 72 are from healthy controls. The donors’ average age is 52 (+/-17) years old, and 56 of them are males. All iPSC lines were verified for their identify based on the matching of snRNA/ATAC-seq-inferred genotypes with their known genotypes. Cells were maintained in feeder-free mTeSR Plus (Stemcell# 100-0276) and passaged using ReLeSR (Stemcell# 100-0483) every 4-6 days following the vendor’s instructions.

## Lentivirus preparation

Lentiviral particles were prepared using low-passage 293T cells maintained in DMEM media supplemented with 10% FBS. 24 hrs prior to transfection, 95% confluent 293T cells were dissociated using Accutase and replated at 1:3 ratio to achieve 70-80% confluence the next day. On the day of transfection, lentiviral plasmids including FUW-rtTA (Addgene# 20342), TetO-Ascl1-puro (Addgene# 97329), Dlx2-hygro (Addgene# 97330), pTet-O-Ngn2-puro (Addgene# 50247) were co-transfected with pMDLg/pRRE (Addgene #12251), pMD2.G (Addgene #12259) and pRSV-Rev (Addgene #12253) at 1:1:1:1 molar ratio using FuGENE HD (Promega). 18 hrs post transfection, DMEM with 10% FBS media was replaced with fresh mTeSR Plus media. 48 hrs post transfection, supernatant containing viral particles was collected and cell debris were removed by centrifugation at 500 × g for 5 min. Supernatant was aliquoted into low-binding tubes and stored at -80°C.

## Neuronal differentiation and co-culture

We differentiated iPSC lines into both excitatory and inhibitory neurons and co-cultured with rat glial cells. For excitatory neuronal differentiation, we used the method for deriving Ngn2-induced glutamatergic neurons(*34*) with minor modifications. On DIV (days in vitro) 0, 60-80% confluent iPSCs were dissociated into single cells using Accutase and replated into Matrigel-coated 6-well plate at 5 × 10^5^ cells per well in mTeSR Plus media with 5μM ROCK inhibitor, together with appropriate amount of rtTA virus and Ngn2-puro virus. On DIV1, media was refreshed with mTeSR Plus containing 5μM ROCK inhibitor and 2μg/ml doxycycline. On DIV2 to DIV4, cells were treated with neural culture media (Neuralbasal media with 1× B27Plus and 1× Glutamax) supplemented with 2μg/ml puromycin, 2μg/ml doxycycline to remove non-transduced cells. On DIV5 to DIV6, puromycin was withdrawn and cells were treated with neural culture media with 2μg/ml doxycycline. On DIV7, cells were ready for replating.

For generating excitatory neurons, we used the method for deriving Ascl1/Dlx2-GABAergic neurons(*35*) with minor modifications. On DIV0, 60-80% confluent iPSCs were dissociated into single cells using Accutase and replated into Matrigel-coated 6-well plate at 7 × 10^5^ cells per well in mTeSR Plus media with 5μM ROCK inhibitor together with rtTA virus, Ascl1-puro virus and Dlx2-hygro virus. On DIV1, media was refreshed with mTeSR Plus containing 5μM ROCK inhibitor and 2μg/ml doxycycline. From DIV2 to DIV4, cells were treated with neural culture media supplemented with 2μg/ml puromycin, 150μg/ml hygromycin and 2μg/ml doxycycline to remove non-transduced cells. On DIV5 to DIV6, cells were treated with neural culture media with 2μg/ml doxycycline and 2μM AraC. Cells were ready for replating on DIV7.

To co-culture the excitatory and inhibitory neurons, on DIV7, separately cultured Ngn2-glutamatergic neurons, Ascl1/Dlx2-GABAergic neurons, and primary rat cortical astrocytes (Thermofisher; N7745100) were dissociated using Accutase at 37°C for 15-20 min. Ngn2-glutamatergic neurons, Ascl1/Dlx2-GABAergic neurons and astrocytes were replated at 5:5:1 ratio onto Matrigel pre-coated 12-well plate in neural culture media supplemented with 2 μg/ml doxycycline, 10 ng/ml BDNF, 10 ng/ml GDNF, 10 ng/ml NT-3, and 1% FBS. For each co-culture, we pooled cells from 3-4 donor lines with equal proportion. On DIV8, we refilled cells with more media. From DIV9 to DIV33, media was refreshed every three days with half volume changes. Doxycycline was withdrawn at DIV14 and 1 µM AraC was added in the media from DIV8 to DIV17 to ensure neuron purity. DIV33 neurons were used for KCI stimulation.

## KCI stimulation of neuronal cultures

To model neuronal activation, we followed a previously used protocol to treat the cells with KCI.(*32*) Briefly, one day before stimulation, old media was aspirated and DIV33 neuron co-culture were silenced overnight in neural culture media supplemented with 1 μM TTX and 100μM DL-AP5. The next day, we added 0.45 volume (e.g., 0.45 ml for 1 ml original culture media) of warmed depolarization solution (10 mM HEPES, 170 mM KCl, 1 mM MgCl_2_, 2 mM CaCl_2_) to initiate KCI stimulation. We prepared three conditions with different treatment durations (0 hr, 1 hr and 6 hrs). After stimulation, cells were dissociated for processing for 10× Genomics sequencing library preparation.

## Single nuclei multiomics sequencing library preparation

After stimulation, cells were briefly washed with 1× PBS and dissociated in Accutase at 37°C for 40 min with gentle shaking. After Accutase incubation, we pipetted cells multiple times and filtered suspension twice with 40μm tip filter (Sigma# BAH136800040-50EA) to obtain single cells. The washed cells were subjected to fresh nuclei isolation following 10× Genomics’ protocols (CG000365 and CG000338). For each library, around 15,000 freshly isolated nuclei were loaded for GEM capture targeting 10,000 nuclei recovery. The pre-amplified DNAs (for snATAC-seq) and cDNAs (for snRNA-seq) were shipped to the University of Minnesota Genomics Center (UMGC) for sn-multiomics sequencing library construction following 10× Genomics’ standard protocol.

## Immunocytochemistry

For Immunocytochemistry, iPSCs/neurons were fixed with 4% PFA in PBS at room temperature for 15 min. After three brief washes in PBS, cells were permeabilized with 0.5% Triton X-100 in PBS for 15 min at room temperature and further blocked with 3% BSA and 0.1% Triton X-100 in PBS at room temperature for 1 hr or 4ºC overnight. After blocking, samples were incubated with primary antibodies diluted with blocking buffer at room temperature for 1 hr or 4ºC overnight. After 3 PBS washes, samples were incubated with secondary antibodies diluted in blocking buffer at room temperature for 1 hr followed by 3 more PBS washes. Then samples were incubated in PBS containing 1 μg/ml DAPI (4', 6-diamidino-2-phenylindole) at room temperature for 10 min. After DAPI staining, samples were washed once with PBS and mounted on glass slides. The images were taken by a Nikon ECLIPSE C2 confocal microscope.

## BDNF OCR peak deletion by CRISPR/Cas9 editing

For BDNF OCR peak deletion, two gRNAs flanking the targeted region were cloned into pSpCas9(BB)-2A-Puro (PX459) V2.0 (Addgene# 62988) respectively. Two iPSC lines were used for editing. For editing, 24 hrs prior to transfection, 60-80% confluent iPSCs were dissociated into single cells using Accutase and replated into Matrigel-coated 60 mm dish at 4.5 × 10^5^ cells per well in mTeSR Plus media with 5μM ROCK inhibitor. On the day of transfection, 3 μg of plasmid DNA carrying gRNA1 and 3 μg of plasmid DNA carrying gRNA2 were introduced into iPSCs using LipofectamineSTEM (Thermofisher) following vendor’s instruction at 1:2 DNA:reagent ratio. 24-48 hrs post transfection, cells were selected with 0.5 μg/ml puromycin; 48-72 hrs post transfection, cells were selected with 0.25 μg/ml puromycin. Afterwards, antibiotics were withdrawn, and cells were maintained in regular mTeSR Plus until colonies reached appropriate size for picking. After colony picking, genomic DNAs (gDNAs) from collected cell pellets was isolated using QuickExtract DNA Extraction Solution for PCR amplification. Amplified DNAs were loaded on 1% agarose gel for electrophoresis to examine fragment size. gDNAs of the colonies with confirmed peak deletion were used for Sanger sequencing confirmation. The confirmed colonies (2-3) with the expected peak deletion were sub-cloned and expanded for cryopreservation. Please refer to Table S32 for gRNA and primer sequences.

## RNA isolation and qPCR

Total RNA was isolated using RNeasy Plus Kits (QIAGEN) following vendor instructions. For qPCR, 300 ng-1 μg RNAs were reverse transcribed using High-Capacity cDNA Reverse Transcription Kit (Thermofisher) following vendor instruction. cDNAs were diluted with nuclease-free water at 1:10 ratio and qPCR reactions were prepared using Taqman Universal PCR Master Mix (Thermofisher). Reactions were loaded on Roche LightCycler 480 system in a 384-well white plate. The 2–∆∆Ct method was used for RNA expression quantification, with GAPDH as endogenous control. Please refer to Table S32 for qPCR assay information.

## Multiomics sequencing and data quality control (QC)

10× Genomics Chromium single cell Multiome sequencing libraries were sequenced at UMGC on Novaseq S4 platform targeting pair-end (2 × 150bp) 50 K reads per nuclei for ATAC library and 25 K reads per nuclei for gene expression library. Briefly, after raw data collection, Illumina’s BCL2FASTQ software was used to demultiplex and assemble the fastq files corresponding to reads (R1/R2) and indices (i5/i7) for Cell Ranger ARC. The fastq files were subsequently processed by 10× Genomics Cell Ranger ARC (v2.0.2) and aligned twice to both the human GRCh38.p14 genome and a contingent of human GRCh38.p14 and mouse GRCm38 provided by 10× Genomics for efficient identification and removal of rodent astrocytes. For snATAC-seq, the per-library Transcription Start Sites (TSS) enrichment score is usually 7-9, and the Fraction of high-quality Reads overlapping Peaks (FRiP) is usually 40-80%. We excluded cells with >15% of the reads mapped to mitochondrial genes, cells with > 8,000 or < 400 number of features, and cells with > 40,000 or <500 number of UMI counts (filtered by 400 < nCount < 8,000 using Seurat).

## Multiomic data analyses

### Barcode level identification of cell line identity

For each library that contained cells derived from 3-4 iPSC lines, barcode-level identification was performed separately for snRNA-seq (GEX) and snATAC-seq data with genotyping information of all donors. Briefly, the BAM files generated from GEX and ATAC assays (gex_possorted_bam.bam and atac_possorted_bam.bam) were processed by demuxlet(*82*) with known genotype information provided as .vcf files. Barcodes (cells) identified as singlets with P1 likelihood (p1LLK) < 1 × 10^-8^ were collected, and only barcodes positively identified in both GEX and ATAC assays were retained for downstream analysis.

### snRNA-seq data analyses

snRNA-seq (GEX) data were processed by extracting gene expression data from the .h5 files generated by Cell Ranger ARC. Only barcodes (cells) confidently assigned to individual iPSC lines were retained. With Seurat 5.1.0(*83*), To assign cell type identity, Leiden clustering was performed at the library level based on the first 30 PCs (or appropriate as determined by the elbow plot) and subsequently comparing cell clusters and their marker gene expressions (*GAD1*, *GAD2*, *SLC17A6*, *SLC17A7*, *NEFM*). The library-level gene expression matrices were subsequently collated in Seurat 5.1.0(*83*) as one large object with cell line-specific metadata assigned. Harmony(*84*) was used to integrate the library-level gene matrices and removed sequencing batch-derived effects.

### snATAC-seq data analyses

For snATAC-seq data, the aggregation function of Cell Ranger ARC was used to merge and generate new fragment files for MACS2-based peak calling with the CallPeaks() function in Signac(*85*) with default settings. We made two merged fragment files, one containing all libraries from batch 024 (18 cell lines) and the other from batch 018, 024, 025, and 029 (76 cell lines). From each fragment file, we iterated through the combinations of cell types (NEFM+ glut, NEFM- glut, GABA) and stimulation stages (0 hr, 1 hr, 6 hrs), which generated nine peak sets (cell type × time). We further generated a union peak set from batch 024 by setting CallPeaks(combine.peaks = TRUE) for peak analysis across different cell types. Finally, peaks that fell within the ENCODE blacklisted regions were removed. We also removed peaks that fell within chromosomes X and Y and the mitochondrial genome.

## Approximation of developmental stages for iPSC-derived neurons

We used the reference single-cell dataset of human embryonic and postmortem brains(*38*) to evaluate our iPSC-derived neurons and their comparable developmental stages. Briefly, the excitatory neuron (ExNeu) and interneuron (IN) populations were extracted using the identity assigned in the original publications. The excised data were then processed as documented in the original publication with Harmony-assisted data normalization, scaling, and dimension reduction using the first 30 PCs to generate the anchor gene set, UMAP dimensions, and unimodal UMAP coordinates for projection. The three main cell types identified (npglut, nmglut, GABA) from snRNA-seq were used as queries. Each cell type was firstly randomly subset to 10,000 cells to reduce computational demands. Subsequently, each cell subset was separately projected by the MapQuery() function to its corresponding cell map (npglut/nmglut to ExNeu and GABA to IN) to show their approximate developmental stages in the human brain.

## PCA analysis

For PCA analysis, we generated pseudo-bulk count matrices from merged snRNA-seq data using the AggregatedExpression() function from Seurat, and each sample represented one cell line in one of three main cell types and its stimulation stage. To further eliminate the interference from sequencing batch-specific effects, we ran regression using the ComBat-seq() function from the R package sva(*86*) using sequencing batch information as the factor. The sva-adjusted pseudo-bulk count matrices were log-transformed and estimated for their observation-level weights using the voom() function from the R package limma(*87*). Finally, we calculated the principal components (PCs) with the prcomp() function and plotted the samples using their first two PCs using the fviz_pca_ind() function from the R package factoextra (<https://cran.r-project.org/package=factoextra>).

## Pseudo-bulk RNA-seq differential gene expression analysis

Gene differential expression (DE) analysis was performed using the R package limma. Briefly, the ComBat-seq-adjusted pseudo-bulk count matrices generated from PCA analysis were used as the initial input. Low-expression genes whose Count Per Million (CPM) value was less than < 1 in half of lines in any of the 3 time points were removed. The matrices were log-transformed and observation-level weights were calculated by voom(). Subsequently, we made the design matrix of known covariates (time, batch, age, sex, SCZ-affection status, and the percentage of corresponding cell types). We fitted the linear model using the lmFit() function of limma. After checking the mean-variance trend (SA plot) for gene distributions, the DE values (log_2_FC, *p*-value, and FDR) were calculated using the topTable() function with corresponding coefficients (1 hr vs 0 hr, 6 hrs vs 0 hr, 6 hrs vs. 1 hr) for all three cell types.

## MAGMA gene set enrichment analysis

We performed MAGMA analysis using MAGMA version 1.08b(*88*) to evaluate the enrichment for the GWAS risk of several psychiatric disorders (SCZ, Neuroticism, ASD, BP, and MDD)(*7, 10, 89-92*), as well as the GWAS set of Crohn’s disease that served as a control set(*93*) (Table S33). The method was adapted from our previous publications with modifications(*63*). Specifically, we compiled the MAGMA-required gene annotation data files based on the more recent GRCh38/hg38 genome to get the gene-SNP annotation file. With the gene-SNP annotation file, we then performed gene-level analysis on SNP *p*-values using the reference SNP data of 1,000 Genomes European panel (g1000_eur_hg38, --bfile) and the pre-computed SNP *p*-values from each disorder’s GWAS data set. The sample size (ncol=) was derived from either GWAS data frames or specified according to the affiliated README data. Subsequently, the result files (--gene-results) from the gene-level analysis were read in for competitive gene-set analysis (--set-annot), where we used default setting (correct=all) to control for gene sizes in the number of SNPs and the gene density (a measure of within-gene L.). The gene-set analysis produced the output files (.gsa.out) with competitive gene-set analysis results that contained the effect size (BETA) and the statistical significance of the enrichment of each gene set (upregulated/downregulated) for each disorder’s GWAS data set.

## Gene ontology enrichment analysis

GO term analysis was performed for the DEG sets specific to each cell type and expression pattern. Briefly, the list of DE genes was used as the SynGo(*94*) input and all the expressed genes in the corresponding cell type were used as the background gene list. A similar approach was used to calculate the enrichment of different gene expression patterns against a multitude of known NPD gene sets.

## Differential peak accessibility analysis

DA peak accessibility was performed using aggregated pseudo-bulk counts based on the three cell-type-specific peak intervals (nmglut, npglut, GABA). Briefly, the AggregatedExpression() function was used to generate cell line × peaks matrices using the cell-type-specific peak intervals (approximately 300 K peaks per cell type). The ComBat_seq() function from sva was subsequently applied to correct group bias for each count matrix. We removed all low-count peaks (CPM < 1 in at least half of the cell lines), and approximately 200 K of the original 300 K peaks survived filtration. The matrices were log-transformed and observation-level weights were calculated by voom(). Subsequently, we applied design matrices of known covariates (time, batch, age, sex, SCZ-affection status, and the percentage of corresponding cell types) as we did in DE gene analysis. We fitted the linear model using the lmFit() function of limma. The DA values (log_2_FC, *p*-value, and FDR) of peaks for each cell type and contrast were calculated using the topTable() function with corresponding coefficients (1 hr vs 0 hr, 6 hrs vs 0 hr).

## Stratified linkage disequilibrium score regression (sLDSC) for GWAS enrichment analysis

sLDSC(*95*) analysis was performed by using the hg38 version of European genotype data (SNPs) from 1000 Genomes Phase 3 and v2.2 baseline linkage disequilibrium/weights. Briefly, linkage disequilibrium score estimations were pre-calculated from the hg38 version of the 1000 Genomes EUR file set (w_hm3_no_hla.snplist), window size 1 cM (ld-wind-cm 1). We used the summary statistics of major psychiatric disorders and non-psychiatric diseases for partition heritability, with several data sets lifted over from hg19 to hg38 when necessary. Disease-specific regressions were performed independently using hm3 SNP weights against each disease for cell-type-specific analysis.

## Integration of snATAC-Seq Profiles and Peak Calling for caQTL and gene regulatory network analyses

To integrate the snATAC-seq data, we utilized ArchR. Quality control measures were applied by filtering cells with a Transcription Start Site (TSS) enrichment score of at least 4 and fragment counts of at least 1,000. After quality control and integration of expression data, we retained a total of 552,653 cells. To call peaks from the snATAC-Seq data, pseudo-bulk replicates were generated for each cell type and for each time point. To ensure equal representation of all cell types, we followed the default setting of ArchR, and sampled a maximum of 500 cells and 25 million fragments from each pseudo-bulk replicate. With the pseudo-bulk replicates, 501-bp fixed-width peaks were called using MACS2(*96*) and integrated in ArchR. Peaks were required to be reproducible in at least two samples, and sex chromosomes were excluded in the analysis. To ensure high-quality peak detection, we limited the number of peaks to a maximum of 250,000 for each cell type and time point context. Consensus peaks were obtained through an iterative overlap peak merging approach in ArchR. This method allowed for robust and reproducible peak identification across different samples and conditions.

## Single-cell Embeddings

To reduce computational cost, we downsampled 18 cell lines (115,855 cells in total) from Batch 024 for all analyses related to gene regulation, including dimensionality reduction, chromVAR analysis, pseudo-time inference, peak-gene mapping, and gene regulatory network construction. Using ArchR, we performed dimensionality reduction on snRNA-Seq and snATAC-Seq data separately, utilizing the iterative latent semantic indexing approach. To create a more robust two-dimensional representation using UMAP, we combined the embeddings of expression and peak accessibility, subsequently correcting for batch effects using Harmony.

## Inference of Pseudo-time

Pseudo-time trajectories were estimated for the GABA, nmglut, and npglut cell types separately to model the transitions between neuron states from unstimulated neurons to neurons stimulated for 1 hr and 6 hrs. Based on the UMAP constructed in the previous section, cell-type-specific trajectories were inferred using the addTrajectory() function in ArchR. We fine-tuned the sparsity of the spline fit and quality filters for the trajectory of each cell type to avoid overfitting of smoothing spline. Cells were divided into 100 bins based on pseudo-time, and the average activities were calculated by pooling cells with similar pseudo-time values.

## Co-activation analysis to link OCRs to genes

To link OCRs to potential target genes, we assessed the correlation of gene expression and normalized chromatin accessibility for all ORCs within 500 kb of genes. All differentially expressed genes were included in the analysis. For each cell type, peaks present in at least 5% of the cells were considered. Gene expression was regressed on peak accessibility using a negative binomial mixed regression implemented using lme4 R package (DOI: https://doi.org/10.18637/jss.v067.i01) with the following formula:

$$Expression_{g} \sim Accessibility_{p}+CellLine\_ID + log(Gex\_LibrarySize)+log(Gex\_MitoRatio)$$

where:

- $Expression_{g}$ represents the raw counts of gene expression for gene $g$,

- $Accessibility_{p}$ denotes the normalized chromatin accessibility of peak $p$,

- $CellLine\_ID$ is a categorical variable representing cell line identity, included as a random intercept to account for donor-specific effects,

- $Gex\_LibrarySize$ refers to the single-cell level library size from the snRNA-Seq data, captured by the number of reads per cell,

- $Gex\_MitoRatio$ captures the single-cell level percentage of mitochondrial reads from the snRNA-Seq experiment, serving as a measure control for cell quality.

To control for multiple testing, the Benjamini–Hochberg procedure was applied to calculate the false discovery rate (FDR). OCR-gene pairs were considered significant and biologically relevant if they had an FDR ≤ 0.05 and a positive regression coefficient.

## Activity-by-Contact (ABC) analysis

We employed the Activity-by-Contact (ABC) model(*46, 97*) to predict enhancer-gene regulatory relationships. For our analysis, we utilized snATAC-Seq data from all 18 samples in batch 024. Additionally, we incorporated time-specific bulk Micro-C data derived from one cell line (CD-11), averaged across all cell types, which allowed us to capture the dynamic nature of chromatin interactions during different neuronal stages. Our analysis aimed to predict enhancer-gene pairs across nine distinct contexts, defined by different combinations of time and cell type. To achieve this, we called peaks for each specific context and subsequently predicted ABC scores under these conditions. An enhancer-gene pair was classified as significant if its ABC score was greater than or equal to 0.021, a threshold recommended by the software to achieve 70% recall.

## Gene Module Analysis

To cluster genes based on their expression trajectory in pseudo-time, we first selected 5,221 differentially expressed and highly variable genes, using criteria of FDR ≤ 0.05 and abs (LogFC) > 1 in at least one differential expression test. We then normalized expression for each gene separately, so that genes with similar trajectory patterns would be clustered together, irrespective of their absolute expression levels. Using the average expression values from 100 pseudo-time points across three cell types, we performed K-means clustering with 15 cluster centers to group the genes into 15 distinct modules. Gene Ontology enrichment analysis was conducted using the Enrichr package(*98-100*), and GWAS enrichment analysis was performed in MAGMA. We inferred chromatin accessibility trajectories for the 15 gene modules by mapping all peaks to the genes within each module, as described in the previous section.

## Defining candidate TFs regulating early and late responses

We used chromVAR to estimate single-cell level enrichment of TF activity. With motif annotations curated from Cis-BP, we estimated chromVAR motif deviations implemented in the ArchR toolkit. We also had single-cell expression from scRNA-seq for all TFs. For each TF, we assessed its role in early and late responses by testing differential motif activity and expression between conditions, one cell type at a time. Specifically, a TF was considered a candidate TF for early response if it showed both higher motif activity and increased expression at 1 hr compared to 0 hr. Similarly, a TF was considered a candidate TF for late response if it showed higher motif activity and elevated expression at 6 hrs compared to both 1 hr and 0 hr. Differential expression analysis was performed using the limma-voom analysis, and differential motif analysis was performed using one-tail Wilcoxon signed-rank test using single-cell level motif deviations estimated by chromVAR.

## Gene Regulatory Network Analysis

To construct the gene regulatory network, we followed these steps: (1) Identify Potential TF Regulators: We identified a set of potential TF regulators by selecting TFs that were differentially expressed in at least one condition. We also ensured that a TF’s motif activity (estimated by chromVAR) and its expression activity were positively correlated. Specifically, we filtered TFs based on the correlation between their expression trajectories and motif activity trajectories, retaining only those with a Spearman’s correlation > 0.3. (2) Check Motif Presence in Open Chromatin Regions (OCRs): We examined the presence of the TF motifs in OCRs linked to each gene, using an FDR threshold of ≤ 0.1. (3) Correlate TF Motif Activity with Target Gene Expression: For motifs present in at least one peak associated with a gene, we correlated the motif activity of TFs with the expression of the target genes. We retained the TF-gene pairs that exhibited a Spearman’s correlation > 0.5 between the motif activity of the TF and the expression of the target gene. This methodology allows us to identify key regulatory relationships between TFs and their target genes, revealing insights into the underlying gene regulatory networks the cellular transitions observed in the pseudo-time trajectories.

## eQTL mapping

To associate the genotypic variation and gene expression variation within each context (combination of cell types and time points), we used the scRNA read count matrix of 36,601 features and 548,800 cells from 100 cell lines and three cell types (i.e., GABA, nmglut, npglut). We first generated pseudo-bulk matrices by summing up the read counts by each feature for cells within a certain time point, a certain cell type, and a certain cell line. We removed samples with less than 1 million total reads. We also removed genes that were not protein-coding, in mitochondria, with less than 2,000 reads across all cells, or without HGNC symbol. After filtering, we retained the pseudo-bulk read count matrix of 14,818 genes by 824 samples. Then, we performed trimmed mean of M values (TMM) normalization and inverse Normal Transformation. The resulting gene expression matrix served as input for MatrixeQTL, along with covariates of cell type composition, gender, age, SCZ affection status, 5 genotype PCs, and 15 gene expression PCs. For genotypes, we removed SNP with minor allele frequency (MAF) below 5%. 5,966,820 SNPs remained for eQTL testing. We used *cis*-variants 250 kb up- and downstream of the gene body. This *cis* window size and the number of gene expression PCs were chosen to optimize the number of eGenes (i.e., genes with at least one significant eQTL) discovered. Our scheme for defining significant eQTL is detailed by the standard procedure of MatrixeQTL(*101*).

In addition to the eQTL mapping in each context, we also performed eQTL mapping by aggregating all contexts. We removed the 5 cell lines from batch 22 and summed up the read counts per cell line per contexts. TPM normalization was applied to the summed reads and then we further summed up all contexts within the same cell line. We proceeded to eQTL mapping with the resulting 95 samples (cell lines) by applying TMM, INT, and MatrixeQTL. This is the same procedure as described above, except that the number of expression PCs was 21 instead of 15. eQTL results from this approach are called “pseudo-bulk eQTL” in subsequent sections.

## Dynamic eQTL testing

We tested the genetic effect heterogeneity across 3 times points, one cell type at a time. We used the following procedure to choose the candidate set of gene-SNP pairs to be tested for each cell type. We considered only eGenes found in at least one time point. Genes that were only significant in pseudo-bulk eQTL were not considered. For each of these eGenes, we started with its eQTL(s) with the smallest nominal P values per condition. Then we took a union of these top eQTLs along with the pseudo-bulk eQTL. There are at most four eQTLs for each eGene in a cell type. We then performed LD pruning with r2<0.1 on these eQTLs within the same eGene.

For the candidate gene-SNP pairs, we used CellRegMap (*102*) to test the heterogeneity of genetic effects across different contexts (time points in our study) from eQTLs. We have n samples (95 cell lines in our case), and each sample belongs to one of K contexts (3 in our case). We use *Z* ($n\times K$ matrix) as the design matrix, encoding whether a sample belongs to a certain context. Under the CellRegMap model, the genetic effect of a variant on expression of a gene has two components: an average effect denoted as $\beta$, and a context-specific effect, denoted as $u_{k}$ for the k-th context. The model assumes that the context-specific effects follow a normal distribution, $u_{k}\sim N(0,\sigma^{2})$. To test if the genetic effects vary across context means testing if $\sigma=0$.

We write the regression model in a matrix form, equivalent to the assumption described above:

$$y = G\beta+ G\gamma+ W\psi+ \mu+ \epsilon$$

where $y$ is gene expression jointly normalized across three time points for each cell type (dimension is n = 95x3), $G$ is genotype, $W$ are covariates, $\mu$ is the individual random effect, $\epsilon$ is the iid error term, and $\beta$ and $\psi$ are fixed effects corresponding to genotypes and covariates, respectively. The focus of the model is $\gamma$, the random slope for genotypes. $\gamma$ is a vector with n dimensions, following multivariate Normal distribution:

$$\gamma\sim MVN(0,\sigma^{2} ZZ^{T})$$

CellRegMap follows StructLMM's idea of using Rao's score test to evaluate the null hypothesis that $\sigma$ is 0. According to CellRegMap, this is the highest power test when many contexts were jointly tested. We used Bonferroni to adjust nominal P values in each gene, retain eQTLs with the minimum adjusted P-value per gene, and then applied Benhamini-Hochberg FDR control to all the remaining eQTL.

## TORUS enrichment analyses of eQTLs and ASoC variants

We applied a Bayesian hierarchical model (TORUS) to perform SNP-based enrichment analysis, testing the enrichment of certain features with eQTL and ASoC variants (*62, 103*). Briefly, TORUS associates the prior probability that a variant is causal to gene expression with the annotations of the variant with a logistic regression model. TORUS then uses this prior in a simple fine-mapping model (assuming a single causal variant per genetic locus) of eQTL data. TORUS uses Maximum Likelihood to estimate the parameters of the logistic regression. In dynamic eQTL enrichment analysis, we used the upregulated peaks in the matched cell type. To assess the enrichment of ASoC in eQTL, we applied the ASoC in matched condition as the neuronal activity eQTL. ASoC SNPs were treated as binary annotations: 1 being within the significant ASoC and 0 otherwise. We used SNPs that did not pass the ASoC testing (FDR>5%) for comparison. For testing disease GWAS enrichment, TORUS assumes that every variant is a risk variant or not, represented by a binary indicator variable (1 or 0). The prior probability of the indicator of a SNP being 1 depends on its annotations. Here, we tested ASoC SNPs categorised by their cell type × time against a selection of diseases/traits GWAS databases. All the annotations are encoded as binary (1 if an SNP is found in the corresponding GWAS database, 0 otherwise). We performed univariate analysis in TORUS to assess the enrichment of each ASoC cell type × time combinations. The GWAS datasets used for enrichment/TORUS analysis were from multiple sources, including NPDs, neuro-related traits, and control disorders/traits, as listed in Table S33.

## Analysis of genetic effect sharing between neuronal activity eQTL and GTEx and ASoC.

We used Storey’s $\pi1$ analysis to analyze the sharing between our eQTL and other summary statistics. This is a common approach to investigate sharing of eQTL under different cell types. For each context in our eQTL and each tissue in GTEx brain eQTL, we obtained all eQTL (FDR<0.05) in a context and estimated the proportion of non-null tests ($\pi1$, Pi1) based on the binomial *p* values of these gene-SNP pairs in a GTEx brain tissue. A similar approach was taken when we computed $\pi1$ for our dynamic eQTL in GTEx brain tissues.

We also used Storey’s $\pi1$ analysis to investigate the sharing between our eQTL and ASoCs. For each ASoC SNP with FDR<0.05, we located its nearest gene and obtained the nominal *P* values of the corresponding eQTL in the matched context if possible. Then, we estimated non-null proportions of these *P* values.

## caQTL Mapping

We calculated pseudo-bulk counts from ATAC-seq for 94 GABAergic neuron cell lines and 95 Glutamatergic cell lines under each time point. The peak insertion counts were initially normalized for library size using the trimmed mean of M values (TMM) method. Subsequently, we applied a rank-based inverse normal transformation (INT) to standardize the accessibility of each peak to a standard normal distribution, utilizing the RNOmni package(*104*). These counts served as the input for caQTL analysis. We employed TensorQTL (DOI: https://doi.org/10.1186/s13059-019-1836-7) for caQTL mapping. To adjust for batch effects and confounding factors, we included three genotype principal components and between four to nine chromatin accessibility PCs as covariates in our analysis:

$$Accessibility_{p} \sim Genotype_{i} + P{C^{G}}_{1}+ ... +P{C^{A}}_{n}$$

where $Accessibility_{p}$ denotes the normalized chromatin accessibility of peak $p$, $Genotype_{i}$ genotype of a *cis*-SNP $i$, $P{C^{G}}_{n}$ are genotype principle components, and $P{C^{A}}_{n}$ are the chromatin accessibility principle components,

For each peak, we conducted 1,000 permutations and computed beta-approximated *P*-values of the top QTL. To account for multiple testing, we calculated Q-values using the qvalue package (DOI: 10.18129/B9.bioc.qvalue). Peaks with a Q-value ≤ 0.05 were considered significant and designated as cPeaks (caQTL peaks). This threshold controlled the FDR and ensured the reliability of our findings.

To investigate time-dependent QTL effects for each cell type, we included the union of cPeaks from all time points. This inclusion ensured that all cPeaks from different time points were considered. For each cPeak, we identified and tested the SNP from the strongest QTL across all time points. This SNP served as the representative variant for assessing QTL effects. Similar to caQTL mapping, we first normalized the library size using counts per million (CPM), then applied rank-based INT to each peak. To test time-dependent effects, we modified the caQTL test above, including Time, and Genotype-Time interaction term. We included 3 genotype PCs, 5 chromatin accessibility PCs, and their interaction effects with time as covariates and ran linear regression as:

$$Accessibility_{p} \sim Genotype_{i}+Time_{t} +Genotype_{i}*Time_{t} + P{C^{G}}_{1}+P{C^{G}}_{1}*Time_{t}+ ... +P{C^{A}}_{5}+P{C^{A}}_{5}*Time_{t}$$

where $Time_{t}$is a categorical variable of the duration of KCl stimulation.

We employed an ANOVA test to compare the full model (including the time-genotype interaction effects) with a reduced model (excluding the time-dependent effect). This allowed us to assess the significance of the time-dependent effect on QTLs. FDR correction was applied to adjust for multiple comparisons, ensuring the robustness of our results. To validate the test statistics and avoid inflation, we performed 1,000 permutations. This permutation approach verified that the observed test statistics were not artificially inflated, thereby enhancing the reliability of our time-dependent QTL effect analysis.

## ASoC mapping

We applied an improved, two-step ASoC mapping with WASP to calibrate for alternate-allele bias effect and to gain more accurate results. BAM files generated from Cell Ranger ARC (atac_possorted_bam.bam) were firstly split and re-merged by cell line using barcodes from initial demultiplexing. Each line-specific BAM file included aligned reads of all cell types and three stimulation stages. GATK (4.2.6.0) HaplotypeCaller was applied to each BAM file (cell line), which identified and extracted all heterozygotic SNP sites and homozygotic SNP sites carrying alternative alleles. The extracted SNP coordination was subsequently used to generate the positional reference of WASP. Next, the original BAM files from Cell Ranger ARC were re-split at the cell line × cell type × stimulation stage (time) levels to generate raw BAM files for WASP calibration. For a set of BAM files from the same line, WASP calibration was performed at the individual BAM files using the line-specific SNP heterozygosity information to re-align reads carrying alternative alleles with bwa and remove any bad read pairs. A second round of GATK HaplotypeCaller was used to identify heterozygous SNP sites and their counts in each calibrated BAM file to generate corresponding VCF files as recommended by the GATK Best Practice (software.broadinstitute.org/Gatk/best-practices/) with VariantRecalibrator (-an DP -an Q.D. -an F.S. -an SOR -an M.Q. -an ReadPosRankSum -mode SNP -tranche 100.0 -tranche 99.5 -tranche 95.0 -tranche 90.0) and reference databases including HapMap v3.3 (priority = 15), 1000G_omni v2.5 (priority = 12), Broad Institute 1000G high confidence SNP list phase 1 (priority = 10), Mills 1000G golden standard INDEL list (priority = 12), and dbSNP v154 (priority = 2). Heterozygous sites with tranche level >95.0% were extracted. And only SNPs with corresponding rs# records found in dbSNP v154 were retained. The individual VCF files were subsequently merged at the cell type × stimulation stage level to increase the statistical power (each merged output included 96-100 lines as cell type varied). After merging, Biallelic SNP sites (GT: 0/1) with minimum read depth count (DP) ≥ 30 and minimum reference or alternative allele count ≥ 2 were retained. The binomial *p*-values (non-hyperbolic) were calculated using the binom.test(x, n, P = 0.5, alternative = “two.sided”, conf.level = 0.95) from the R package, and Benjamini & Hochberg correction was applied to all qualified SNPs as the correcting factor of R function p.adjust(x, method = "fdr"). We set the threshold of ASoC SNP at FDR value = 0.05.

## Homer enrichment analysis of TF-binding motifs for ASoC SNPs

We used HOMER(*105*) to assess the enrichment of TF binding motifs in sequences flanking ASoC SNPs (±25 bp) adapted from our previous investigations(*62*). We used the JASPAR database (2022 release) with all 522 human TF motifs. For each cell type × time, we used the ASoC SNPs (FDR < 0.05) as input intervals. The parameters used were findMotifsGenome.pl <input.bed> hg38 -cpg -mknown jasper2018.known <output>. HOMER provided the genetic background for the enrichment test, and the enrichment significance was derived from HOMER.

## Brain eQTL enrichment analysis for ASoC SNPs

We performed eQTL enrichment analysis to investigate the differences in the association between ASoC and non-ASoC SNPs in GTEx cortex-eQTL(*91*) and PsychENCODE eQTL(*106*) databases with adaptation(*62*). For ASoC/non-ASoC SNPs in each set of cell type × time combination, the SNPs were assigned to genes (eQTL targets) if the distance of the SNP and its corresponding gene TSS was less than 500 kb, and the SNP was associated with the expression of the corresponding gene at FDR < 0.05.

## Micro-C analysis of ASoC SNPs and their targets

The Micro-C experiment was conducted in co-cultured Ngn2-Glut and GABA neurons of two donor lines (CD11, CD12) at 0 hr, 1 hr, and 6 hrs post-stimulation. Dovetail™ Micro-C (Pan-promoter Capture) kit was used process the samples. To minimize background interference from cell-line discrepancies, we used the samples derived from a single line (CD11) from comparing different time points. The Micro-C data was performed by an external vendor and analysed using ChiCAGO(*107*) at 500 bp resolution. To assign the micro-C targets of each SNP, we firstly associated each SNP (0 hr, 1 hr, 6 hrs) with its nearest interacting micro-C fragment (from 0 hr, 1 hr, 6 hrs samples) if their distance was less than 2.5 kb (upstream/downstream). If such an association coould be established, we extracted the other end of the interacting micro-C fragment and checked if it overlapped with the promoter region (-2 kb / +1 kb of TSS). If the other fragment fell into the promoter region, we added the original SNP to the pool of ASoC SNPs with their corresponding identity.

## Promoter and enhancer enrichment analysis for ASoC SNPs

We defined the interactions of ASoC SNPs and human enhancers/promoters according to our previously developed algorithms with adaptation(*62*). The analysis was similar to that implemented in GREAT and was described previously(*66, 108, 109*) for testing the folds of peak enrichment within the annotated genomic regions and epigenetically annotated regions. To adapt the original algorithm for SNP-based testing, we developed an updated version of the algorithm, and the enrichments were calculated using the formula below:

$$\text{ratio}=\frac{\left( \frac{\text{total number of SNPs in the cell type }\text{}\text{ time combination overlap the features}}{\text{genome size}} \right)}{\left( \frac{\text{total lengths of the features}}{\text{genome size}} \right)\cdot\left( \frac{\text{total SNP number of the cell type }\text{}\text{ time combination}}{\text{genome size}} \right)}$$

For ASoC SNPs that were cell-type-specific or shared by three cell types, we analyzed enrichment considering SNP intervals as the unit similar to OCR peaks. The enrichment *p*-values were estimated based on the binomial test as implemented in GREAT and as previously described(*108, 109*).

$p_{\text{enrichment}}=\sum_{i=k_{\pi}}^{n} \left( \begin{matrix} n \\ i \end{matrix} \right)p_{\pi}^{i}\left( 1-p_{\pi} \right)^{n-i}$

from which we calculated the p-value using the

binom.test(x, n, p, alternative=“greater”) function in R, where

*x* = numbers of SNPs that fell within the designated epigenetically annotated features;

*n* = total length of the SNP intervals (in bp);

*p* = total length of the designated epigenetically annotated features (in bp) / genome size (3.2 × 10^9^ bp)

The Gene-based annotation of the genome was derived from GENCODE v35 as part of the built-in database of the updated HOMER package(*105*). The list of human forebrain/non-forebrain enhancers was from the VISTA enhancer browser(*110*). Human-gained enhancers were acquired from GSE63648 using data from either the frontal or occipital cortex at 12 PCW and marked by H3K27ac or H3K4Me2(*111*). The definitions of chromatin state were assembled using an imputed 25-state model derived from individual #E081 of fetal brain tissue by the Roadmap Epigenomics Project(*109, 112*). For estimating the enrichment of ASoC SNPs (Fig. 2A), the categories of epigenomic features used for promoter and enhancer annotations were promoter = TssA, PromU, PromD1 and PromD2; enhancers = TxReg, TxEnh5, TxEnh3, TxEnhW, EnhA1, EnhA2, EnhAF, EnhW1, EnhW2, EnhAc, and DNase. The abbreviation of different chromatin states was promulgated below, as used in(*109*):

| TssA | Active TSS |
| --- | --- |
| PromU | Promoter Upstream TSS |
| PromD1 | Promoter Downstream TSS with DNase |
| PromD2 | Promoter Downstream TSS |
| Tx5’ | Transcription 5’ |
| Tx | Transcription |
| Tx3’ | Transcription 3’ |
| TxWk | Weak transcription |
| TxReg | Transcription Regulatory |
| TxEnh5’ | Transcription 5’ Enhancer |
| TxEnh3’ | Transcription 3’ Enhancer |
| TxEnhW | Transcription Weak Enhancer |
| EnhA1 | Active Enhancer 1 |
| EnhA2 | Active Enhancer 2 |
| EnhAF | Active Enhancer Flank |
| EnhW1 | Weak Enhancer 1 |
| EnhW2 | Weak Enhancer 2 |
| EnhAc | Enhancer Acetylation Only |
| Dnase | DNase only |
| ZNF/Rpts | ZNF genes & repeats |
| Het | Heterochromatin |
| PromP | Poised Promoter |
| PromBiv | Bivalent Promoter |
| ReprPC | Repressed PolyComb |
| Quies | Quiescent |
| Forebrain_enh | Forebrain enhancers |
| Non_forebrain_enh | Non-forebrain enhancers |
| H3K27acF | H3K27 acetylated regions found in the frontal lobe |
| H3K27acO | H3K27 acetylated regions found in the occipital lobe |
| H3K4me2F | H3K4me2 regions found in the frontal lobe |
| H3K4me2O | H3K4me2 regions found in the occipital lobe |

## Integrative analysis of NPD GWAS with neuronal activity eQTL or caQTL

Causal TWAS (cTWAS) (*56*) was used to analyze the heritability of NPD mediated by the molecular traits from our QTL and ASoCs. Compared to other TWAS approaches, cTWAS employs a regression model that effectively reduces false positives by jointly analyzing all nearby variants and genes. Given the genetically predicted expression of all genes (as in standard TWAS), cTWAS assumes a regression model:

$$y=\sum_{j} \beta_{j}\tilde{X}_{j}+\sum_{m} \theta_{m}G_{m}+\epsilon, \epsilon\sim N(0,\sigma^{2})$$

where *β*_j_ and *θ*_m_ are the effect sizes of gene expression j and the variant m, respectively. cTWAS assumes a spike-and-slab prior for the effects of gene expression and variants, respectively. The parameters of these distributions are estimated through an expectation-maximization (EM) algorithm, using all the data from the genome. Once these parameters are estimated, cTWAS solves the regression model above with SuSiE, a fine-mapping method. The results would be Posterior Inclusion Probabilities (PIPs) for all the expression traits and variants. While the model is formulated using individual level data, the method can be used with summary statistics. In that case, a reference LD matrix matching the GWAS samples is used.

The cTWAS model above has only gene expression in a single context. But it is easy to extend the model to allow multiple groups of QTL in the same model, with exactly the same algorithm. For example, we could have eQTL from multiple tissues or cellular contexts; or one eQTL group and one caQTL group, in the model. We used this version of cTWAS in our study. This integration can combine evidence across multiple groups to improve power. For example, if we have eQTLs across several contexts in the model, then we can obtain the PIP of a gene in each of the contexts. These PIPs can be combined to obtain the probability that the gene is causal in at least one context (denoted as gene PIP). Meanwhile, we can also quantify the importance of each context, defined as the ratio of PIP of that context over the total PIP across all contexts. This allows us to assign causal context for a putative causal gene.

Another advantage of cTWAS is that it can estimate (1) the proportion of heritability mediated by each group of molecular traits and (2) fold enrichment of molecular traits compared to background SNPs. These parameters are derived from the prior parameter estimation step:

$$enrichment=\pi_{G}/\pi_{v}$$

$$mediated h^{2}g=PVE_{G}/(PVE_{G}+PVE_{v})$$

where $\pi_{G}$ is the estimated prior of a group of molecular trait, $\pi_{v}$ is the estimated prior of background SNPs, $PVE_{G}$ is the proportion of variance explained by a group of molecular traits, and $PVE_{v}$ is the proportion of variance explained by the background SNPs.

When we used cTWAS to integrate eQTL with NPD traits, we first extract top eQTL per gene per context and use these eQTL as the genetic prediction models (denoted as “weights”) of the gene expression traits. cTWAS then includes eQTL data across all 9 contexts in analysis. We note that even though we analyzed all 9 contexts together, a particular gene may occur only in a subset of 9 contexts, as it may not have eQTL in all contexts. However, one problem is that our eQTL study has incomplete power, thus for a particular gene, we may miss some relevant contexts. To address this issue, we used a relaxed threshold. Suppose a gene has eQTL in one context (say C1), but no eQTL in the second context (C2). If the top eQTL in C1 has p value < 0.1 in C2, then this eQTL would be included as the weight in C2. This allows us to include as many contexts as possible for a single gene.

To run cTWAS, we used the default setting, allowing at most 5 causal signals per locus. We used the LD reference of UK Biobank White British samples. For ADHD GWAS, we initially found a large number of putative causal genes, suggesting that LD mismatch between the reference and GWAS samples may be a problem. We thus ran ADHD analysis with the option of *L* = 1, i.e., allowing at most a single causal signal per locus. Under this setting, the results are independent of the reference LD matrix.

We used the same procedure when applying cTWAS to caQTL. The difference is that we complemented caQTL with ASoC SNPs. In the case where caQTL and ASoC have overlapped peaks, we chose the one with smaller nominal *P* values. In addition, we used a more stringent threshold of nominal *P* values at 0.05 instead of 0.10 to increase confidence in the results and reduce the number of molecular traits included.

## Single-cell differential gene expression analysis in SCZ neurons

We performed DEG analysis between neurons from SCZ donors and those from controls to identify genes that exhibited SCZ case-control differential neuronal activation. Briefly, we first subset the snRNA-seq data of the 28 SCZ lines and the sex/age-matched 28 controls (17 males and 11 females in SCZ cases with an average age of 48; 16 males and 12 females in controls with an average age of 50). The subset Seurat object was first split by individual x cell type and processed by standard Seurat SCTransform for data transformation. Subsequently, all transformed libraries were re-integrated by Seurat IntegrateLayers using “HarmonyIntegration” method to construct the master Seurat object for MAST analysis using the zlm hurdle model. We used covariates including cell co-culture batch, sex, age, and cell type fraction in MAST test. The Seurat wrapper FindMarkers was used to find DE genes between SCZ case and control groups. Only genes expressed in at least 5% of cells in either comparison group were used. We recalculated FDR for the result of each context (cell type/ timepoint). FDR < 0.05 was used as cut-off for DEGs used for the gene set enrichment analyses.

## Statistical analyses

For genetic analyses (eQTL and caQTL mapping and GWAS enrichment analyses), different statistical methods were used and specified in the method section above and in different figure legends. For other assays, unless otherwise specified, Student’s *t*-test (between two groups) or the Kruskal Wallis test with Dunn’s multiple comparisons and *P*-value adjustment (more than two groups) was used to determining significance between groups. Samples were assumed to be unpaired and have non-parametric distribution unless otherwise specified. Data were analyzed using R 4.3.2 and GraphPad Prism 10. Results were considered as significant if *P* < 0.05 (*: *P* < 0.05; **: *P* < 0.01; ***: *P* < 0.001; ****: *P* < 0.0001). All data are reported as mean ± SEM.

# Supplemental Text

## Sn-Multiomics data QC and preliminary analysis

The sequencing reads of 1,053,422 nuclei (Tables S1-2) were aligned to a hybrid human/rat genome using 10x Genomics Cell Ranger ARC (v2.0.2) and analyzed in Seurat 5.1.0(*83*). We performed data quality control (QC) by sequencing batch and after merging all batches (Fig. S1A). For each batch, we demultiplexed(*82*) different donor lines (n=2-4) and confirmed the clear separation of clusters of iGlut (SLC17A6+) and iGABA (GAD1+/GAD2+) on Uniform Manifold Approximation and Projection (UMAP) (Fig. S1B-G). For snATAC-seq data, we transferred the cell identity labels from RNA-seq data on UMAP of gene-activity-score (gact) (Fig. S1H-I) and verified the robust transcription start site (TSS) read enrichment (>5) (Fig. S2A-D, Table S2). After merging all the post-batch-QC data, we obtained 651,012 neurons with an average of 7,510 unique molecular identifiers (UMI) per nucleus that are comparable across the 100 lines (Table S3, Fig. S4A-C). We defined three major subtypes of neurons: GABA (n=251,501), NEFM+ Glut (npglut; with stronger NEFM expression) (n=159,735) and NEFM- Glut (nmglut; with weaker NEFM expression) (n=137,564) (Fig. 1E-H, Fig. S4D-F, Table S1), with reproducible cell type clustering patterns across samples (Fig. S3A-E). Compared to single-cell transcriptomic profiles of brain excitatory and inhibitory neurons(*38*), our iGlut and iGABA are mostly similar to neurons of early brain developmental stages (from 2^nd^ trimester to 2 years old; predominately from 2^nd^ trimester) (Fig. S5A-F).

## Transcriptomic and epigenomic landscape of cell-type-specific neuronal activation and its relevance to NPD

With sn-Multiomics data of co-cultured human Glut/GABA neurons and mouse glia, we first identified differentially expressed genes (DEGs) upon stimulation by KCI (1 hr vs. 0 hr, and 6 hrs vs. 0 hr) in each neuron subtype. To minimize possible batch effects, we analyzed data from 18 donor lines of the same sequencing batch (batch 24; Table S1) and corrected for co-culture batch effect (Fig. S7A-B). We found that 65-82% genes were either up- or down-regulated in any cell type/time point (1 or 6 hrs) at FDR<0.05. The number of DEGs and their log_2_ fold changes (log_2_FC) from analysis of the 18 donor lines were very similar to those from 76 donor lines (Fig. S7C-D; Table S4). The upregulated genes showed much larger magnitude of expression changes than the downregulated genes, with the well-known ERGs (e.g., *FOS*, *FOSB*) exhibiting the largest FC (Fig. S7D, Table S4).

To examine the biological relevance of these activity-dependent DEGs, we performed gene set enrichment analyses. Analysis of synaptic gene ontologies (SynGO)(*113*) showed that only the upregulated genes showed significant enrichment for synaptic genes (Fig. S8A). Our MAGMA(*114*) analysis found that common GWAS risk of NPD or traits had higher enrichment in upregulated genes than downregulated or unchanged genes, with strongest enrichment for SCZ (Fig. S9A). We also found a lack of enrichment for autism spectrum disorders (ASD), which may be due to the relatively small number of ASD GWAS risk loci. We thus performed an enrichment analysis using a set of 102 ASD risk genes from rare variant analysis(*40*), together with a set of SCZ rare variant-based risk genes(*39*) and other sets of GWAS risk genes for SCZ(*41*), bipolar disorder (BP)(*67*), major depressive disorder (MDD)(*68*), and post-traumatic stress disorder (PTSD)(*115*) (Table S5). We found strong enrichment for ASD risk genes among the upregulated genes across cell types, and to a less extent for SCZ rare variant-based risk genes (Fig. S8B). Interestingly, the majority of SCZ rare risk genes were upregulated by stimulation, rather than downregulated (Fig. 2B, Fig. S8C). We also confirmed the pseudobulk-based expression changes of some selected SCZ and ASD genes in single neurons (Fig. S8D) and validated the downregulation of *SNAP91* (a SCZ GWAS risk gene) and *IMMP2L* (a GWAS risk gene for SCZ and MDD) by qPCR in independent cell cultures (Fig. S8E).

We next analyzed the snATAC-seq (Fig. 1F, Fig. S1H) to characterize the landscape of activity-dependent OCR peaks upon stimulation. For the same 18 donor lines used for DEG analysis, we identified 150K to 300K peaks across cell types/timepoints (76 donor lines gave a similar number of peaks) (Fig. S9B). There were more peaks at 1 and 6 hrs (Fig. S9B), suggesting a robust effect of neuronal stimulation on global chromatin accessibility. About 19-22% of peaks are stimulation-specific across cell types (Fig. S9C). To identify OCR peaks that showed differential accessibility (i.e., DA peaks) upon neuronal stimulation in each cell type, we used limma(*116*) to analyze a set of merged peaks (170K for GABA, 196K for nmglut, and 207K for npglut). We found that 26-34% and 40-51% of the peaks showed DA at 1 hr and 6 hrs, respectively (Fig. S10A-C; Table S6), with more upregulated peaks than downregulated ones. Similar to DEGs (Fig. S7D), the upregulated peaks often showed larger magnitude of changes (Fig. S10C). As expected, *FOS* showed the largest increase of peak accessibility at 1 hr, highlighting its driver role as ERG in inducing activity-dependent gene expression (Fig. S10C). The OCR peak of *FOS* with the strongest accessibility increase at 1 hr is ~4.2 kb downstream of its TSS with a conserved CEBP binding motif in the middle of the peak that showed peak-to-gene link (Fig. S10C-D), indicating this peak and its accessibility to CEBP may mediate the immediate expression of *FOS* upon stimulation. We next assessed the DA peak enrichment for SNP heritability of NPD (Fig. S10F, Fig. S10E). We found that only the DA peaks at 1 or 6 hrs of stimulation but not those static peaks (i.e., chromatin accessibility unaltered) showed GWAS enrichments (Fig. S10E). Taken together, our results highlight the widespread effects of neuronal stimulation on cell-type-specific transcriptomes and chromatin accessibility as well as their relevance to NPD.

## Activity-dependent expression of BDNF is regulated by cell-type-specific OCRs

*BDNF* is a SCZ risk gene(*41*) that encodes a neurotrophin important for neuronal differentiation and synaptic plasticity(*117, 118*). Despite being a well-established LRG, its cell-type-specific regulation is unknown. We found that *BDNF* exhibited late response in both Glut and GABA cells (Fig. 1I-J, Fig. S7D, and Fig. S9C), but the DA peaks upon stimulation were different between cell types (Fig. S9D, Fig. S11 A, and Table S6). The DA peak showing the strongest increase of accessibility in npglut and nmglut was about 49 kb upstream (i.e., putative enhancer) of the TSS of *BDNF* (Fig. S9D, Fig. S11A), but with much weaker accessibility in nmglut. However, the DA peak showing the largest increase of peak accessibility in GABA was near the 3’-UTR of *BDNF*. These DA peaks showed peak-gene promoter linkage at 1 hr and/or 6 hrs of stimulation and correlated with *BDNF* expression in their respective cell types (Fig. S9D, Fig. S11A), suggesting their possible enhancer function.

We next validated whether the DA peak 49 kb upstream of the TSS of *BDNF* indeed regulated the activity-dependent *BDNF* expression in iGlut. We noted that this DA peak encompassed the binding site of early response AP-1 TFs (FOSB, JUNB, JUND, FOSL1, FOSL2) (Fig. S9E), suggesting an enhancer role of the OCR peak in regulating *BDNF* expression. We thus CRISPR/Cas9-engineered two iPSC lines (CD07 and CD15) by deleting the OCR peak (~600bp) (Fig. S9E, Fig. S11B-C). We then differentiated the two isogenic pairs of CRISPR/Cas9-edited iPSC lines into iGlut and carried out KCI stimulation. We found that while *BDNF* expression was similar between the unedited and deleted lines at 1 hr, its expression was significantly lower at 6 hrs of stimulation in the OCR-deleted lines (Fig. S9F). This supports the role of this OCR in regulating the activity-dependent expression of *BDNF* in iGlut. These results highlight that similar transcriptional responses across cell types may have distinct epigenomic regulatory mechanisms.

## Integrative multiomic analysis identifies regulatory program of neuronal activation

While some TFs, e.g., FOS and JUNB, are well known regulators of early response, the regulatory programs controlling cell-type-specific late response are far less clear. We thus leveraged our multiomic data to identify putative TF regulators. We limited the analysis to TFs that showed differential expression and motif enrichment in at least one condition. We used chromVAR(*119*) to assess a single-cell level motif enrichment score in each of the 9 contexts (3 cell types, 3 time points). Combining motif scores with gene expression, we classified the role of each TF in early or late response. For a given cell type, we considered a TF to be a candidate regulator of early or late response when it showed higher motif enrichment and gene expression at 1 hr (vs. 0 hr), or at 6 hrs (vs. 1 hr), respectively (Table S13).

We identified 145 candidate TF regulators of early response, nearly half of which are shared by all cell types (Fig. S13A). Some shared TFs with the largest motif changes from 0 to 1 hr are well-known early response TFs, e.g., FOS, JUNB, EGR1, and NPAS4, while some others have less established roles in early response, e.g., BACH2, JDP2, MAFK, and SMARCC1 (Fig. S13B). We noted that the expression of the shared TFs tends to be elevated transiently at 1 hr, but their motifs remain enriched at 6 hrs (e.g., FOS, JUNB, and NPAS4) (Fig. S13B, 4D), suggesting that the epigenomic changes established by these early response TFs were maintained at a later stage.

Our analysis of candidate late response regulators revealed a very different pattern. We found 64 candidate TFs in at least one cell type. Compared to early response TF regulators, these TFs are much more cell-type-specific (Fig. S13C). Only 6 TFs were shared across cell types, including some important for neurodevelopment, such as MEF2C and ESR1 (Fig. S13B, D). This highlighted the distinct regulatory programs controlling late responses in Glut and GABA neurons.

To understand the cell-type-specific responses, we focused on TFs whose roles are limited to only Glut or GABA cells. For Glut cells, we observed a relatively large number of candidate TFs regulating late response. The TFs showing strongest motif enrichment at 6 hrs are regulators of early response in all cell types, such as the AP1 family TFs (FOSL, JUND) and a member of SWI/SNF family (SMARCC1) (Fig. S13B). While the motifs of these TFs are also enriched in GABA cells, their motif enrichment is much stronger at 6 hrs in Glut cells, and their expression are also upregulated in Glut cells (Fig. S13B). These results thus suggested that a subset of early response TF regulators continue to drive late response, primarily in Glut cells.

For GABA cells, we found 21 TF candidates for late response (Table S13). Most of the TFs showed modest changes of motif activities between 1 and 6 hrs, with a few exceptions (NR2F2, NR2C1, ESRRA) (Fig. S13B). We hypothesized that some TFs without large motif changes from 1 to 6 hr may still play a role in neuronal response. We thus assessed motif enrichment of TFs at 6 hrs in GABA cells. The TFs showing highest motif enrichment include several GABA-specific TFs, ID3, EVX1, DLX5, and TCF4, a risk gene and a master regulator in SCZ(*49*). These TFs showed high expression and strong motif enrichment even before stimulation (Fig. S13B,D). We also found other TFs likely regulating early response specifically in GABA cells, such as EMX2 and NR4A2 (Fig. S13B,D). In contrast, there were much fewer Glut-specific early response TFs (Fig. S13A). These results thus suggest that distinct TF activities before and during the early response to the stimulation primed the epigenome of GABA cells, leading to varied late responses in GABA cells.

Altogether, our results highlighted a shared regulatory program controlling early responses of all cell types, and distinct programs controlling late responses in Glut and in GABA cells. Some of the key TFs in these programs are NPD risk genes (e.g., MEF2C, TCF4), highlighting the importance of these regulatory programs to the development of NPD.

Supplemental Figures

## Fig. S1. Multiomics data processing and quality control (QC) by batch.

(A) A flowchart depicts raw data processing from FASTQ format to QC-data for downstream analysis. (B) snRNA-seq UMAP shows a representative sequencing library, library 05-0 hr, for cell clustering. (C) Feature plots of the same library show the expression of Rat Gfap (astrocyte marker) and human GAD1 (GABAergic neuron marker). (D) UMAP of the same library shows individual identity of each cell (i.e., demultiplexed three iPSC lines and rat astrocytes). (E) Violin plots of the same library show the distribution of RNA features per cell (nFeature_RNA), UMI counts per cell (nCount_RNA), and the percentage of mitochondrial transcripts per cell (percent.mt). (F) UMAP projection of aggregated snRNA-seq results of sequencing batch 024, including 15 libraries from 18 lines and all three time points (0 hr, 1 hr, and 6 hrs). Rat astrocytes have been removed before library aggregation. (G) The violin plot (left) shows the expression of key genes in each cluster of (F) and the UMAP projection (right) of the assigned major cell type identities.npglut, iGlut with higher NEFM expression; nmglut, iGlut with lower NEFM expression; GABA, iGABA neurons stained positives for GAD1 and GAD2; unknown, cells can not be assigned to either iGlut or iGABA. (H) UMAP of aggregated snATAC-seq data from the same batch (024) with transferred label from snRNA-seq. (I) Gene activity (OCR peak reads) plots (1 kb TSS+gene body) of human GAD1, GAD2 (GABAergic neuron markers) and human SLC17A6, SLC17A7 (glutamatergic neuron markers) of the same ATAC-seq UMAP projection in (H).

## Fig. S2: QC metrics of snATAC-seq.

(A) Representative TSS enrichment plots (1 kb TSS+gene body) for different sequencing libraries, related to Figure 1. (B) The box-whisker plot shows the summarized distribution of TSS enrichment values of all libraries in the study. (C) Representative fragment size distribution patterns in different sequencing libraries. Note the expected pattern of nucleosomal periodical size in each graph. (D) The box-whisker plot shows the summarized proportions of ATAC-seq Fragment of Reads in Peaks (FRiP) for all sequencing libraries in the study.

## Fig. S3: Integrative analyses of snRNA-seq and snATAC-seq for all 100 lines.

(A-B) UMAP projection plots show the merged raw snRNA-seq data from all sequencing libraries, pre- (A) or post- (B) Harmony normalization. (C) UMAP projection of post-Harmony snRNA-seq data stratified by different sequencing batches. (D) UMAP projection of post-Harmony snRNA-seq data stratified by different stimulation time points (0 hr, 1 hr and 6 hrs). (E) UMAP of each sequencing library from (D) show reproducibility of cellular compositon and distribution of different contexts (cell types × time points).

## Fig. S4: Additional QC metrics on the merged snRNA-seq library.

(A) A bar graph shows the number of demultiplexed cells of each of the 100 iPSC lines across three time points. (B) A box-whisker plot shows the distribution of mean snRNA-seq UMI counts/cell of all cell lines across three time points. (C) A box-whisker plot shows the distribution of mean snATAC-seq reads/per cell of all cell lines across three time points. (D) Leiden-based clustering results of the merged snRNA-seq library (resolution = 1). A total of 20 clusters were defined. (E) Violin plots show the expression of key cell type-specific marker genes across all clusters. (F) Feature plots show the absent of expression of pluripotency marker genes POU5F1 and NANOG, and strong expression of neuron-specific marker genes NEFM and MAP2 in the merged library.

## Fig. S5. Projection of iPSC-derived iGlut and GABA neurons to brain excitatory and inhibitory neurons of various developmental stages (Velmeshev 2023).

(A) and (B) UMAP projection of all excitatory neurons and inhibitory neuorons, respectively (Velmeshev 2023). (C) and (D) UMAP projection of subsampled 10,000 npglut and nmglut neurons (this study), respectively, using principal components (PCs) derived from A. (E) UMAP projection of subsampled 10,000 GABA neurons (this study) using PCs derived from B. (F) Percentages of the neurons transcriptionally similar to those matched excitatory or inhibitory neurons of different brain developmental stages.

## Fig. S6. Cell compositions and known early or late response gene expression across three time points.

(A) Reproducible cell compositions (major subtype of neurons) of 100 cell lines across time points. (B) Gene expression and gene activity (at TSS and gene body) of some known early response genes (ERG) in each context (cell type × timepoint). (C) Gene expression and gene activity (at TSS and gene body) of some known late response genes (LRG) in each context (cell type × timepoint). (D) Pseudo-time trajectories of neuron stimulation of each cell type (see Methods). The direction of an arrow in each UMAP points to late response time points along 100 bins of pseudo-time points.

## Fig. S7. Differentially expressed genes (DEGs) upon neuronal stimulation.

(A) Expression variance partitioned to each cellular variable in 18-line snRNA-seq data. Aff, schizophrenia affection status. (B) Pairwise Pearson’s correaltions of the log2FC of each expressed gene at each cellular context (cell type × time point) from DEG analyses in 18 lines and in 76 lines. Note the strongest correlation (near 1) between 18-line and 76-line datasets for the same context. (C) Principal component analysis (PCA) plots show clear separation of each sample by time point for each cell type (GABA, left; nmglut, middle; npglut, right). Pseudobulk RNA-seq expression values of all the expressed genes (CPM >1 in at least half of the samples) in 18 lines after correcting for co-culture batch (group; each batch has 2-4 iPSC lines) were used for PCA. (D) Vocalno plots of DEGs at 1 and 6 hrs of stimulation (vs. 0 hr) in each cell type. Red, upregulated; blue, downregulated; FDR < 0.05. In all analyses, pseudobulk RNA-seq data were used. Highlighted genes are some known ERGs and LRGs.

## Fig. S8. Biological relevance of neuron activity-dependent DEGs.

(A) Radial charts show the enrichment of synaptic GO terms in DEGs (from SynGO analysis) for up- or down-regulated genes upon stimulation (1 hr or 6 hr) in each cell type. Note only stimulation-upregulated genes show enrichment of synaptic GO terms. (B) Bar plots show the enrichment of NPD GWAS genes or schizophrenia (SCZ) genes with disease-associated ultra-rare protein truncating variants (from SCHEMA study) and ASD risk genes from exome sequencing study. Up- or downregulated genes were separately analyzed for disease gene set enrichment using Fisher’s exact test (compared to genes unaltered by stimulation). *: P < 0.05, ***: P < 0.001. (C) Log2 (FC) of SCZ GWAS risk genes (prioritized single credible risk gene in PGC3 SZ GWAS study) upon stimulation in each cell type. * indicates a DEG with FDR <0.05. (D) Dot plot shows single neuron activity-dependent expression of some selected NPD risk genes. (E) qPCR confirmation of the mRNA expression changes of two stimulation downregulated SCZ risk genes (IMMP2L and SNAP91) in co-cultured neurons at different stages post-stimulation (using the same cDNAs for snRNA-seq; n=3 libraries). Expression was normalized mRNA level of GAPDH.

## Fig. S9. GWAS enrichment of differentially expressed gene and the mapping of neuronal activity-dependent OCR peak.

(A) GWAS enrichment of differentially expressed gene (DEG; 1 hr vs. 0 hr and 6 hrs vs. 0 hr) sets. Several non-NPD traits were included as control in MAGMA analysis. IBD, inflammatory bowel disease; T2B, type 2 diabetes. (B) Peak counts of major neuron subtypes at different post-stimulation time points using the 18-line or 76-line datasets. Note the comparable number of peaks called from the two datasets. (C) Venn diagram of OCR peaks in iGABA, npglut and nmglut at three time points. (D) Peak-gene linkage plot of BDNF in npglut. The highlighted is the most-upregulated peak and its gene-peak linkage was observed only at 1 hr. (E) The highlighted peak in (D) overlaps with ENCODE CRE and has AP1 TF-binding sites. (F) The boxed OCR region in (E) was deleted by CRISPR/Cas9 editing, which affected BDNF mRNA expression (assayed by qPCR) in iGlut at 6 hrs post-stimulation. n = 3-5 independent cultures of 2 donor lines (CD07 and CD15). Unpaired two-side Student’s t-test.

## Fig. S10. Differentially accessible (DA) peak analysis.

(A) Violin plot shows variances partitioned to each cellular variable in 18-line snATAC-seq dataset. Aff, schizophrenia affection status. (B) Q-Q plot shows the distribution of expected (x, assuming uniform distribution) against the permutated values (-log10P) of DA peaks. Note the minimal p-value inflation in our DA peak analysis. (C) Volcano plots of DA peaks in each cell type upon stimulation. Red dots, upregulated peaks; blue dots, downregulated peaks; FDR< 0.05 as cut-off. (D) Putative OCR peaks that mediates FOS expression in early response. From left to right, panels showing OCR peaks, gene expression and peak-gene linkage at 0 hr, 1 hr, and 6 hrs, respectively. Note the specific peak-gene linkage was only observed at 1 hr post-stimulation and the presence of CEBPE-binding motif in the 3’ OCR of FOS. Only data from npglut is shown. (E) Straified-LDSC analysis of GWAS SNP heritability enrichment in dynamic peak sets (up- or downregulated) for GWAS of several NPD and non-NPD control disorders (e.g., Insomania). Shown are fold of enrichment (bubble size) and significance (color scheme, -log10P). Background SNPs are used as control for enrichment test. (F) SCZ heritability enrichment analysis for up- or down-regulated, or static (unchanged) OCR peaks.

## Fig. S11. DA peaks of BDNF.

(A) The DA peak (green arrow) with strongest gain of accessibility in npglut (in main Fig. 2) upon stimulaltion shows much weaker accessibility in nmglut (left panel) and in GABA (right panel). Note the same specfic peak-gene linkage as in npglut only presents at 1 hr in both nmglut and GABA. Light blue arrow points to a GABA-specific DA peak. (B) PCR confirmation of the CRISPR-edited iPSC lines with the BDNF OCR peak deletion. Note the ~650 bp reduction in fragment size on agarose gel (1%) in CRISPR-edited lines (vs. unedited lines). Two donor lines (CD07, CD15) were used for editing. Each lane represents a different isogenic clone before or after editing. (C) Sanger sequencing confirmation of unedited and CRISPR-edited line carrying the BDNF peak deletion (homozygous). The PAM sequence is highlighted in yellow.

## Fig. S12. Neuron activity-dependent gene expression modules (clusters) and correlation between chromatin accessibility and gene expression for OCR-gene pairs.

(A) Pseudo-time trajectories of normalized chromatin accessibility (red) and gene expression (blue) for all expression clusters. (B) Heatmap of pseudo-time activity of chromatin accessibility and gene expression for identified OCR-gene pairs in GABA and nmglut. For each cell type, the left panel depicts normalized chromatin accessibility of mapped peaks, while the right panel illustrates normalized gene expression. Highlighted are most variable peak-gene pairs. (C) Schematics of the Dovetail Micro-C (Pan promoter capture) experimental design. The co-cultured excitatory and inhibitory neurons before and after KCI stimulation (as in Figure 1A) were processed for Micro-C via three major steps (crosslinking, fragmentation amd hybridization capture), followed by sequencing and data analyses to identify chromatin interaction bins (using CHICAGO). Neuron co-cultures (both iGlut and GABA) of two iPSC lines (CD11, CD12) were used, of which CD11 line has data from all three time points (0, 1, and 6 hrs) of KCI stimulation. (D) Proportion of peak-gene pairs overlapped with ABC-defined peak-gene contacts at different FDR cutoffs.

## Fig. S13. TF regulation of early and late neuronal response and ASD-related gene regulatory network (GRN).

(A) Number of cell-type-specific and shared candidate TFs regulating early responses. (B) Motif activity (bubble size) and expression (color) of selected TFs across time points and cell types. Motif activities are in a Z-score scale. (C) Number of cell-type-specific and shared candidate TFs regulating late responses. (D) Pseudo-time trajectories showing expression (red) and motif activity (blue) of FOS, MEF2C, TCF4, and DLX5.

## Fig. S14. Neuron activation-dependent eQTL mapping and characterization.

(A)-(C) Venn diagram of eGenes in each cell type at three different time points. (D) Proportions (from Pi analysis) of eGenes in neuronal activity eQTL that are also eGenes in GTEx brain tissues. (E) Effect size concordance between nmglut 0 hr and GTEx whole blood. Blue dashed line is diagonal line with slope=1. Red line is fitted to the effect sizes. (F) Comparison of eQTL effects between 0 hr nmglut and GTEx cerebellum or whole blood. The “Direction” bar, concordance of the signs of effects. The “Z-score” bar, effect size correlation. (G) Total proportion of h2g mediated by neuronal activity eQTL across the five NPD phenotypes. (H) The count of risk genes derived by integrating neuronal activity eQTL with GWAS of the five NPD phenotypes. Genes are labelled as “Dynamic” or “Static” according to how PIPs are partitioned across contexts (see Fig. 6C legends). (I) Comparison of eGenes found by cTWAS in neuron activity eQTLs and in GWAS.The group “Shared” dictates the number of high confidence risk genes shared between neuronal activity eQTL cTWAS results and the GTEx brain cTWAS results. The group “Unique” dictates the 16 genes that are only discovered by neuronal activity eQTL cTWAS.

## Fig. S15. caQTL (including ASoC) mapping.

(A) Schematics of neuron stimulation-specific (1 hr and 6 hrs) ASoC SNP site. (B) Volcano plots show the ASoC SNPs (red dots) in each context (cell type × time point). Plotted are allele fractions of the reference alleles (Ref/total read depth) and -log10P of the ASoC test. FDR < 0.05 for ASoC. (C) Venn diagrams show the overlaps of statistically significant (FDR < 0.05) ASoC SNPs of each cell type at three time points. (D) Box plots of two dynamic QTL from nmglut and npglut, illustrating chromatin accessibility stratified by the genotype of the SNP, across time points. Each dot represents log(CPM) (peak accessibility) of the SNP-associated cPeak of an individual cell line. CPM, counts per million reads.

## Fig S16. Enrichment of ASoC SNPs (vs. non-ASoC control SNPs) in brain eQTL and comparison with Micro-C chromatin contact.

(A) Bar plot shows the percentage of ASoC and control SNPs that are also GTEx cortex eQTL across contexts (cell types × time points). Control SNP sets contain random set of SNPs that are not ASoC. (B) Enrichment fold and P-values for ASoC SNPs (vs. control non-ASoC SNPs) in (A). Fisher’s exact test was used. (C) Bar plot shows the percentage of ASoC SNPs and control SNPs that are also PsychENCODE eQTL across contexts (cell types × time points). (D) Proportion of ASoC SNPs that can be assigned to a cis-target based on Micro-C chromatin contacts (gene TSS to OCRs) at matching context (cell type x time point).

## Fig. S17. Enrichment of ASoC SNPs in different regulatory sequence elements.

(A) Bar plot shows the percentage and count of ASoC SNPs of each temporal category located in the annotated enhancer (red bars) or promoter (blue bars) regions. ASoC SNP categories in each cell type include those specific to each time point (0 hr, 1 hr or 6 hrs) and shared by all time points. (B) and (C) Bar plots show the 25-way GREAT functional annotation of ASoC SNPs that are specific to or shared across time points in each cell type. (B) shows the fold of enrichment and SNP counts while (C) shows the GREAT enrichment P-value (-log10P) and SNP counts.

## Fig. S18. Integrative analysis of caQTL and GWAS of NPD phenotypes by cTWAS.

(A) The proportion of h2g mediated by caQTL and ASoCs with or without controlling for eQTL in cTWAS. (B) The number of risk peaks across the five NPD phenotypes stratified by peaks being dynamic or static. See the legend of Fig. 6C for the definition of “dynamic” or “static”. (C) The number of risk peaks across the five NPD phenotypes stratified by the cell type from which the risk peaks were identified. (D) The Venn diagram between risk peaks and risk genes with PIP over 0.5. They are overlapped if the corresponding caQTL/ASoC SNP is located within 500 kb distance from the eQTL SNP. (E) Example locus plots of the risk peaks with SNPs overlapping with a neuronal activity eQTL. For each locus plot, the top panel is GWAS P values of the genes and SNPs, and the bottom panel is cTWAS PIP.

## Fig. S19. Differential expressed genes (DEGs) in neurons from SCZ cases vs. controls.

(A) UMAP projection of snRNA-seq data of neurons from matched SCZ cases and controls. 28 SCZ cases were matched with the same number of controls by sex and ages. (B)-(D) Venn diagram showing SZ-associated DEGs of each cell type at three time points after neuronal activation (0, 1, and 6 hrs) as well as the top 10 enriched GO terms (biological processes) among stimulation-specific DEGs. All the listed GO terms have FDR < 0.05 and are ranked by their combined enrichment score from Enrichr. (E) and (F) Bubble plots show the log2FC and Z-scored -log10P of DE from MAST test for cTWAS (caQTL-based) SCZ genes and PGC3 SCZ GWAS prioritized single genes, respectively. Only genes that are also SCZ-associated DEGs (FDR<0.05) at any context (cell type and time points) are included. (G)-(I) Violin plots of SCZ case-control single-cell expression of example genes in cholesterol metabolic gene set (HMGCS1), SCZ cTWAS gene set (RFC3), and SCZ GWAS prioritized single gene set (PTPTK), respectively. Only the cell type showing the strongest DE in SCZ cases is shown for each gene.

List of Supplemental Tables

(The most relevant tables have been attached to the manuscript as supporting materials. Tables that are too large to submit through the Science submission system are available **at** [**https://doi.org/10.5061/dryad.0zpc8677w**](https://doi.org/10.5061/dryad.0zpc8677w)**.)**

Table S1. Donor information of all the iPSC lines and their summary statistics of sn-multiomics data for all three time points of KCI stimulation.

Table S2. Summary statistics of each sequencing library before and after QC. Each sequencing library is for cells of a co-cultured 2-4 iPSC lines at a particular time point of stimulation. TSS=transcription starting site.

Table S3. SnRNA-seq post-QC summary data for all 100 lines.

Table S4. Differentially expressed genes upon KCI stimulation in each cell type.

Table S5. NPD gene sets used for enrichment analyses.

Table S6. Differentially accessible peaks upon KCI stimulation in each cell type.

Table S7. Genes included in each gene module (cluster).

Table S8. GO terms (biological process) enriched for each gene module.

Table S9. OCR-gene pairs identified through single-cell level peak-gene correlation (co-activation) analysis.

Table S10. Micro-C summary statistics.

Table S11. Micro-C chromatin contacts in each sample. Listed are 5 kb bins.

Table S12. Enhancer-gene pairs predicted by the ABC (Activity-by-Contact) model, with an ABC score ≥ 0.021. Predictions from all nine contexts are included.

Table S13. Differential TF motif activity analysis using a one-tailed Wilcoxon signed-rank test.

Table S14. Gene regulatory networks identified in three cell types.

Table S15. Target genes regulated by the ASD risk TFs in each cell type.

Table S16. GO-term (Biological Process) enrichment for four ASD risk-associated TFs. The FDR was recalculated to account for multiple testing across the four TFs.

Table S17. GO-term (Molecular Function) enrichment of target genes shared by at least three of the ASD risk TFs. Only "molecular function" terms are enriched.

Table S18. TFs with target genes enriched for ASD genes in each cell type.

Table S19. Significant eQTLs in each cell type and time point.

Table S20. Dynamic cGenes in each cell type and time point.

Table S21. eQTL-based cTWAS results.

Table S22. GO term enrichment of cTWAS NPD genes.

Table S23. cPeak and caQTL identified by tensorQTL in each cell type and time point.

Table S24. ASoC SNPs in each cell type and time point.

Table S25. Dynamic caQTLs for each cell type with permutation (n=1000) testing result.

Table S26. Percentage of ASoC SNPs that can be assigned to a Micro-C based cis-target gene in a matching context.

Table S27. ASoC status of Schizophrenia (SCZ) GWAS risk SNPs and their LD proxies (R2>0.8) in each cell type and time point. Brain eQTL and Micro-C target annotations are from column BN to BT.

Table S28. ASoC status of Bipolar disorder (BP) GWAS risk SNPs and their LD proxies (R2>0.8) in each cell type and time point. Brain eQTL and Micro-C target annotations are from column BN to BT.

Table S29. ASoC status of Major depression disorder (MDD) GWAS risk SNPs and their LD proxies (R2>0.8) in each cell type and time point. Brain eQTL and Micro-C target annotations are from column BN to BT.

Table S30. caQTL-based cTWAS results.

Table S31. Result of single cell DE analysis in neurons of each context (Cell type x time point after stimulation) between 28 SCZ cases and matched controls. BH_FDR values were derived from the MAST p-value for each context.

Table S32. sgRNA sequences and PCR primer sequences as well as qPCR assays.

Table S33. GWAS datasets used in enrichment tests (MAGMA, TORUS, sLDSC and cTWAS).

References Only Cited in Supplemental Materials

79. X. Zhao et al., Alzheimer's disease protective allele of Clusterin modulates neuronal excitability through lipid-droplet-mediated neuron-glia communication. medRxiv, (2024).10.1101/2024.08.14.24312009

80. H. Zhang et al., Scaled and efficient derivation of loss-of-function alleles in risk genes for neurodevelopmental and psychiatric disorders in human iPSCs. Stem cell reports 19, 1489-1504 (2024).10.1016/j.stemcr.2024.08.003

81. A. Kozlova et al., Alzheimer's disease risk allele of PICALM causes detrimental lipid droplets in microglia. Res Sq, (2024).10.21203/rs.3.rs-4407146/v1

82. H. M. Kang et al., Multiplexed droplet single-cell RNA-sequencing using natural genetic variation. Nat Biotechnol 36, 89-94 (2018).10.1038/nbt.4042

83. Y. Hao et al., Dictionary learning for integrative, multimodal and scalable single-cell analysis. Nat Biotechnol 42, 293-304 (2024).10.1038/s41587-023-01767-y

84. I. Korsunsky et al., Fast, sensitive and accurate integration of single-cell data with Harmony. Nature methods 16, 1289-1296 (2019).10.1038/s41592-019-0619-0

85. T. Stuart, A. Srivastava, S. Madad, C. A. Lareau, R. Satija, Single-cell chromatin state analysis with Signac. Nature methods 18, 1333-1341 (2021).10.1038/s41592-021-01282-5

86. W. E. Johnson, C. Li, A. Rabinovic, Adjusting batch effects in microarray expression data using empirical Bayes methods. Biostatistics 8, 118-127 (2007).10.1093/biostatistics/kxj037

87. M. E. Ritchie et al., limma powers differential expression analyses for RNA-sequencing and microarray studies. Nucleic Acids Res 43, e47 (2015).10.1093/nar/gkv007

88. N. Y. A. Sey et al., A computational tool (H-MAGMA) for improved prediction of brain-disorder risk genes by incorporating brain chromatin interaction profiles. Nature Neuroscience 23, 583-593 (2020).10.1038/s41593-020-0603-0

89. M. Nagel et al., Meta-analysis of genome-wide association studies for neuroticism in 449,484 individuals identifies novel genetic loci and pathways. Nat Genet 50, 920-927 (2018).10.1038/s41588-018-0151-7

90. D. M. Howard et al., Genome-wide meta-analysis of depression identifies 102 independent variants and highlights the importance of the prefrontal brain regions. Nat Neurosci 22, 343-352 (2019).10.1038/s41593-018-0326-7

91. T. S. W. G. o. t. P. G. Consortium, S. Ripke, J. T. Walters, M. C. O’Donovan, Mapping genomic loci prioritises genes and implicates synaptic biology in schizophrenia. medRxiv, (2020)

92. Z. Zhou, Q. Yuan, D. C. Mash, D. Goldman, Substance-specific and shared transcription and epigenetic changes in the human hippocampus chronically exposed to cocaine and alcohol. Proc Natl Acad Sci U S A 108, 6626-6631 (2011).10.1073/pnas.1018514108

93. J. Z. Liu et al., Association analyses identify 38 susceptibility loci for inflammatory bowel disease and highlight shared genetic risk across populations. Nat Genet 47, 979-986 (2015).10.1038/ng.3359

94. P. D. Thomas et al., PANTHER: Making genome-scale phylogenetics accessible to all. Protein Sci 31, 8-22 (2022).10.1002/pro.4218

95. B. K. Bulik-Sullivan et al., LD Score regression distinguishes confounding from polygenicity in genome-wide association studies. Nat Genet 47, 291-295 (2015).10.1038/ng.3211

96. Y. Zhang et al., Model-based analysis of ChIP-Seq (MACS). Genome Biol 9, R137 (2008).10.1186/gb-2008-9-9-r137

97. J. Nasser et al., Genome-wide enhancer maps link risk variants to disease genes. Nature 593, 238-243 (2021).10.1038/s41586-021-03446-x

98. E. Y. Chen et al., Enrichr: interactive and collaborative HTML5 gene list enrichment analysis tool. BMC Bioinformatics 14, 128 (2013).10.1186/1471-2105-14-128

99. M. V. Kuleshov et al., Enrichr: a comprehensive gene set enrichment analysis web server 2016 update. Nucleic Acids Res 44, W90-97 (2016).10.1093/nar/gkw377

100. Z. Xie et al., Gene Set Knowledge Discovery with Enrichr. Curr Protoc 1, e90 (2021).10.1002/cpz1.90

101. A. A. Shabalin, Matrix eQTL: ultra fast eQTL analysis via large matrix operations. Bioinformatics 28, 1353-1358 (2012).10.1093/bioinformatics/bts163

102. A. S. E. Cuomo et al., CellRegMap: a statistical framework for mapping context-specific regulatory variants using scRNA-seq. Molecular systems biology 18, e10663 (2022).10.15252/msb.202110663

103. X. Wen, Molecular QTL discovery incorporating genomic annotations using Bayesian false discovery rate control. Ann Appl Stat 10, (2016)

104. Z. R. McCaw, J. M. Lane, R. Saxena, S. Redline, X. Lin, Operating characteristics of the rank-based inverse normal transformation for quantitative trait analysis in genome-wide association studies. Biometrics 76, 1262-1272 (2020).10.1111/biom.13214

105. S. Heinz et al., Simple combinations of lineage-determining transcription factors prime cis-regulatory elements required for macrophage and B cell identities. Mol Cell 38, 576-589 (2010).10.1016/j.molcel.2010.05.004

106. S. Akbarian et al., The PsychENCODE project. Nat Neurosci 18, 1707-1712 (2015).10.1038/nn.4156

107. J. Cairns et al., CHiCAGO: robust detection of DNA looping interactions in Capture Hi-C data. Genome Biol 17, 127 (2016).10.1186/s13059-016-0992-2

108. L. de la Torre-Ubieta et al., The Dynamic Landscape of Open Chromatin during Human Cortical Neurogenesis. Cell 172, 289-304 e218 (2018).10.1016/j.cell.2017.12.014

109. C. Roadmap Epigenomics et al., Integrative analysis of 111 reference human epigenomes. Nature 518, 317-330 (2015).10.1038/nature14248

110. A. Visel, S. Minovitsky, I. Dubchak, L. A. Pennacchio, VISTA Enhancer Browser--a database of tissue-specific human enhancers. Nucleic Acids Res 35, D88-92 (2007).10.1093/nar/gkl822

111. S. K. Reilly et al., Evolutionary genomics. Evolutionary changes in promoter and enhancer activity during human corticogenesis. Science 347, 1155-1159 (2015).10.1126/science.1260943

112. J. Ernst, M. Kellis, Large-scale imputation of epigenomic datasets for systematic annotation of diverse human tissues. Nat Biotechnol 33, 364-376 (2015).10.1038/nbt.3157

113. F. Koopmans et al., SynGO: An Evidence-Based, Expert-Curated Knowledge Base for the Synapse. Neuron 103, 217-234.e214 (2019).https://doi.org/10.1016/j.neuron.2019.05.002

114. C. A. de Leeuw, J. M. Mooij, T. Heskes, D. Posthuma, MAGMA: generalized gene-set analysis of GWAS data. PLoS Comput Biol 11, e1004219 (2015).10.1371/journal.pcbi.1004219

115. M. B. Stein et al., Genome-wide association analyses of post-traumatic stress disorder and its symptom subdomains in the Million Veteran Program. Nature Genetics 53, 174-184 (2021).10.1038/s41588-020-00767-x

116. M. E. Ritchie et al., limma powers differential expression analyses for RNA-sequencing and microarray studies. Nucleic Acids Research 43, e47-e47 (2015).10.1093/nar/gkv007

117. M. V. Chao, Neurotrophins and their receptors: a convergence point for many signalling pathways. Nat Rev Neurosci 4, 299-309 (2003).10.1038/nrn1078

118. H. Park, M. M. Poo, Neurotrophin regulation of neural circuit development and function. Nat Rev Neurosci 14, 7-23 (2013).10.1038/nrn3379

119. A. N. Schep, B. Wu, J. D. Buenrostro, W. J. Greenleaf, chromVAR: inferring transcription-factor-associated accessibility from single-cell epigenomic data. Nat Methods 14, 975-978 (2017).10.1038/nmeth.4401
