# Supplementary figures and images for: Single-cell multiomics of neuronal activation reveals context-dependent genetic control of brain disorders
Source: bioRxiv. 2025 Feb 17:2025.02.17.638682. Preprint. [Version 1] doi: 10.1101/2025.02.17.638682 (PMC11870544; doi:10.1101/2025.02.17.638682)

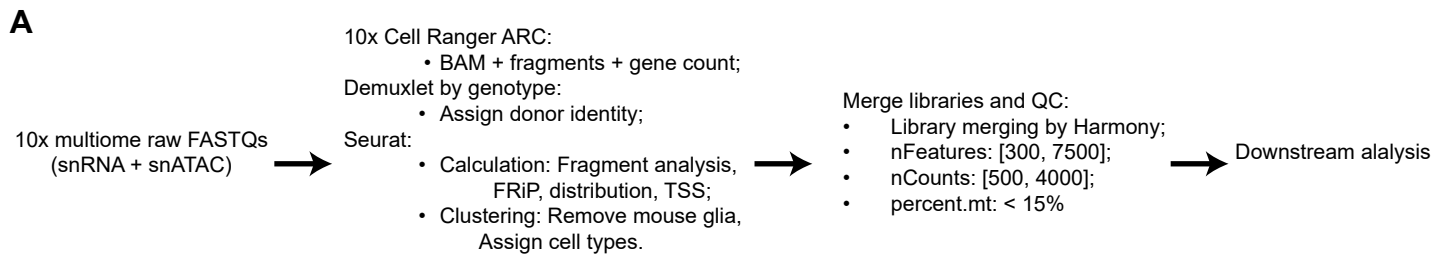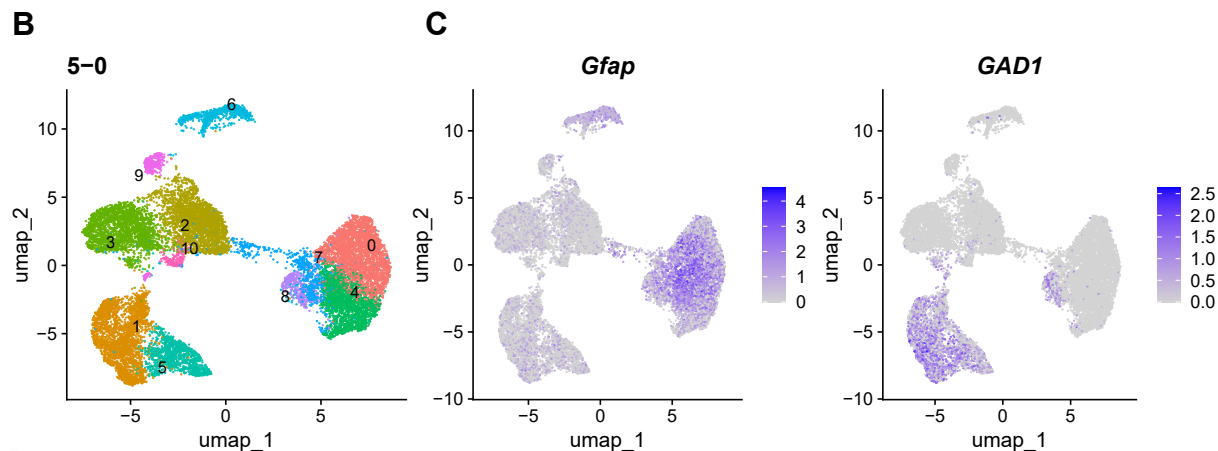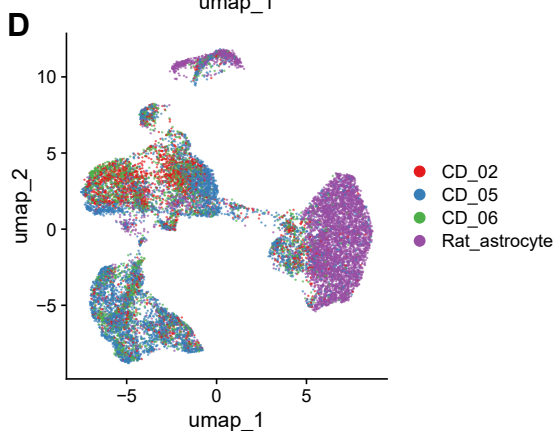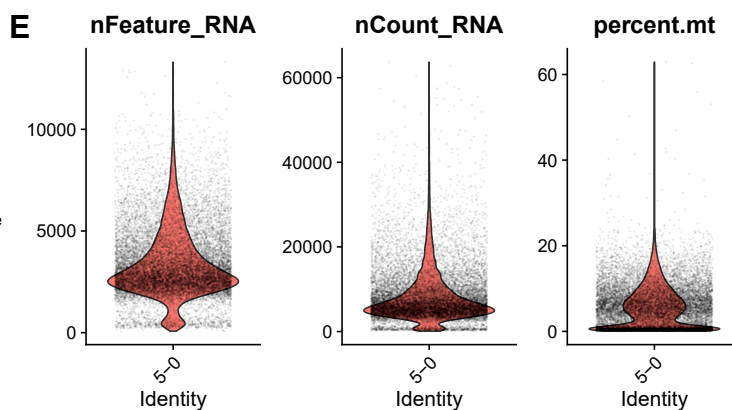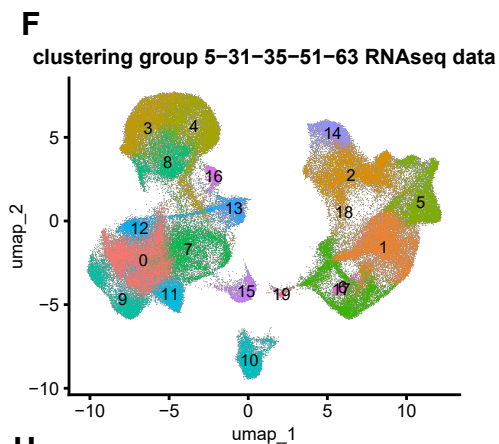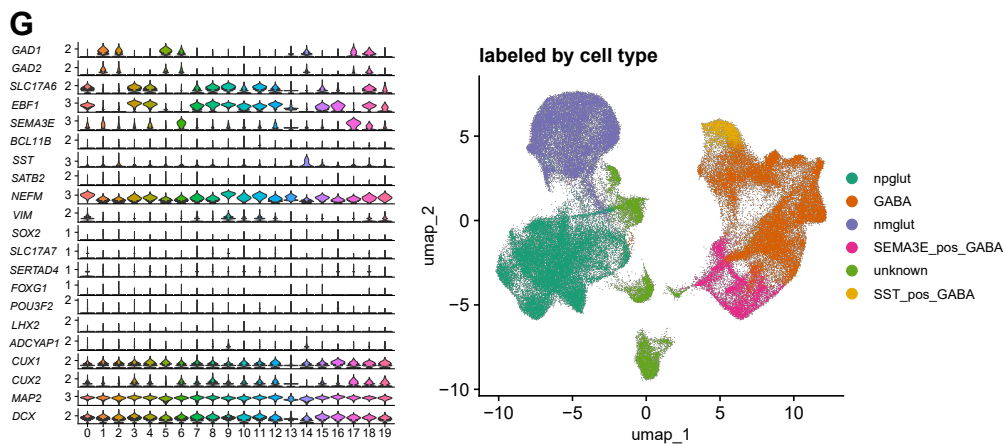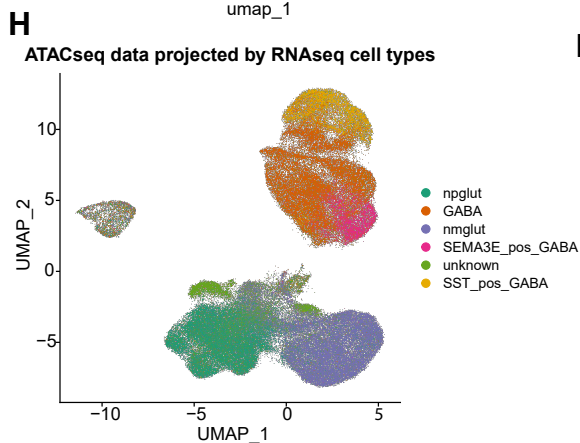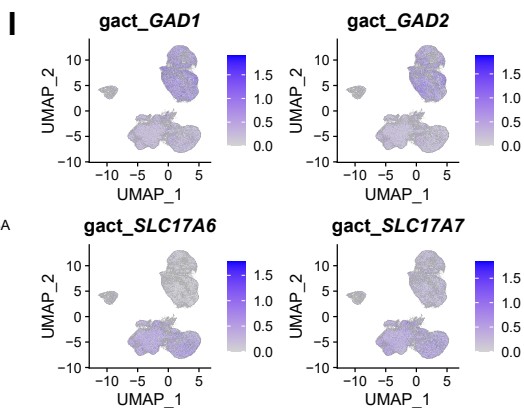

Supplement: Supplement 2 [file media-2.pdf]

**A****TSS enrichment**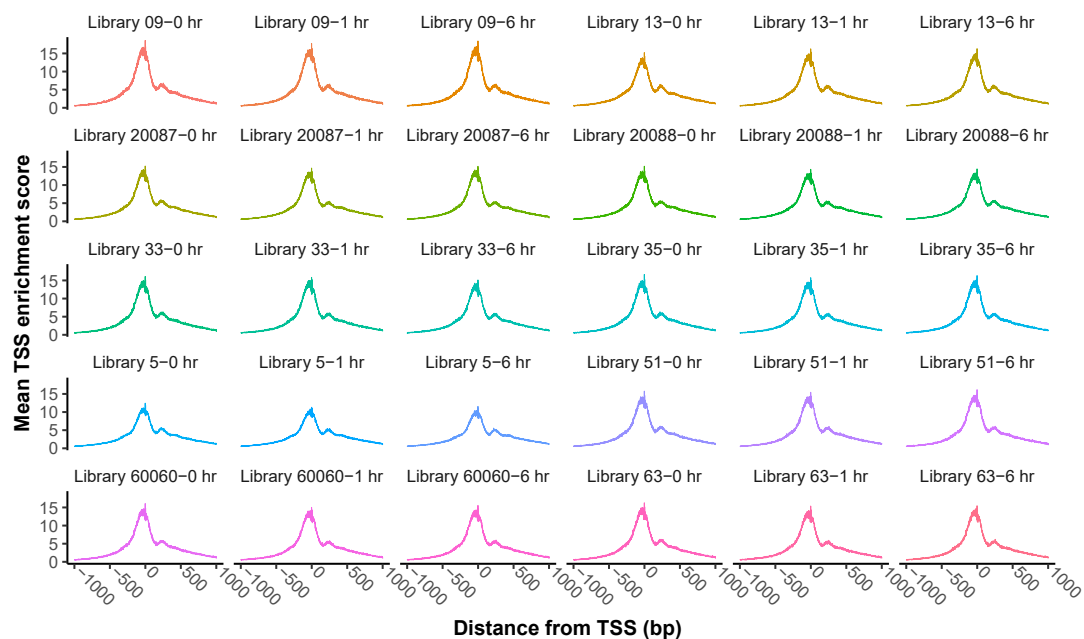**B**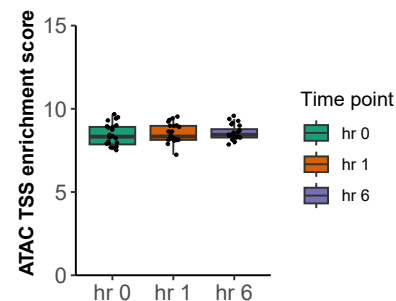**C****Fragment size histogram**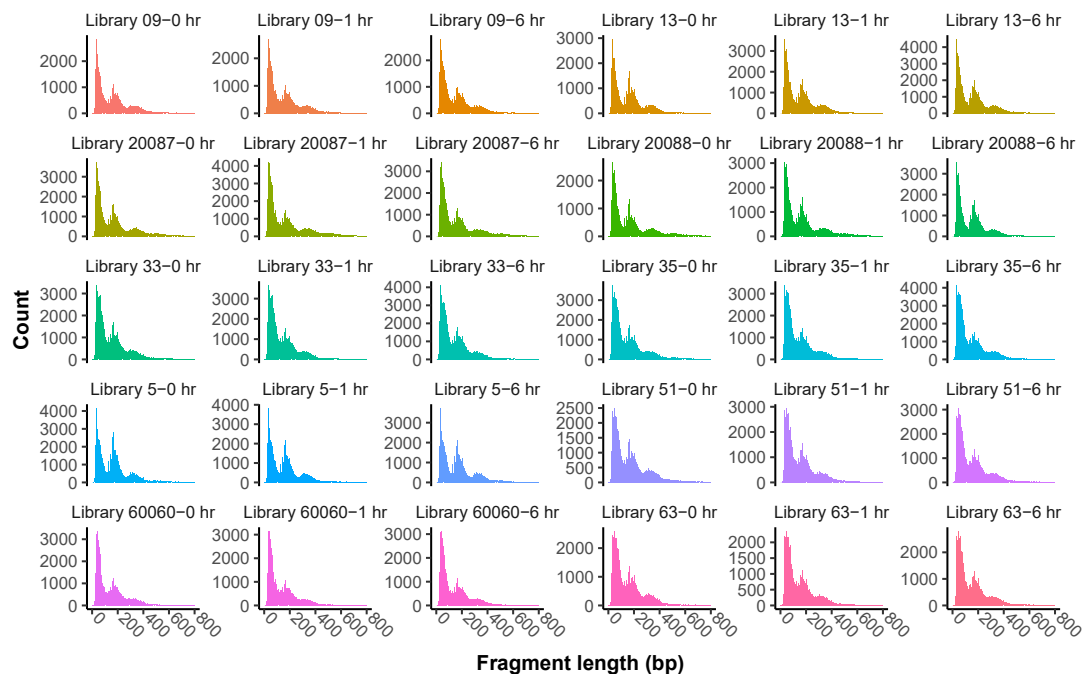**D**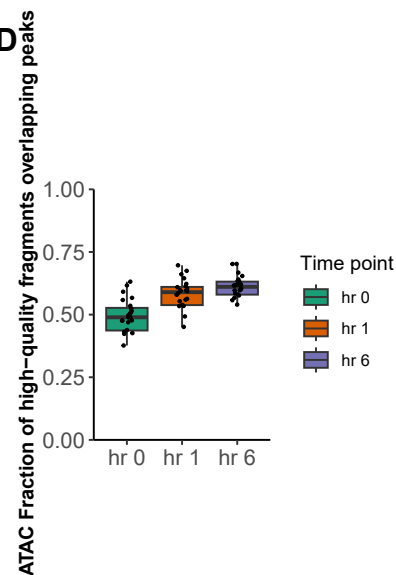

Supplement: Supplement 3 [file media-3.pdf]

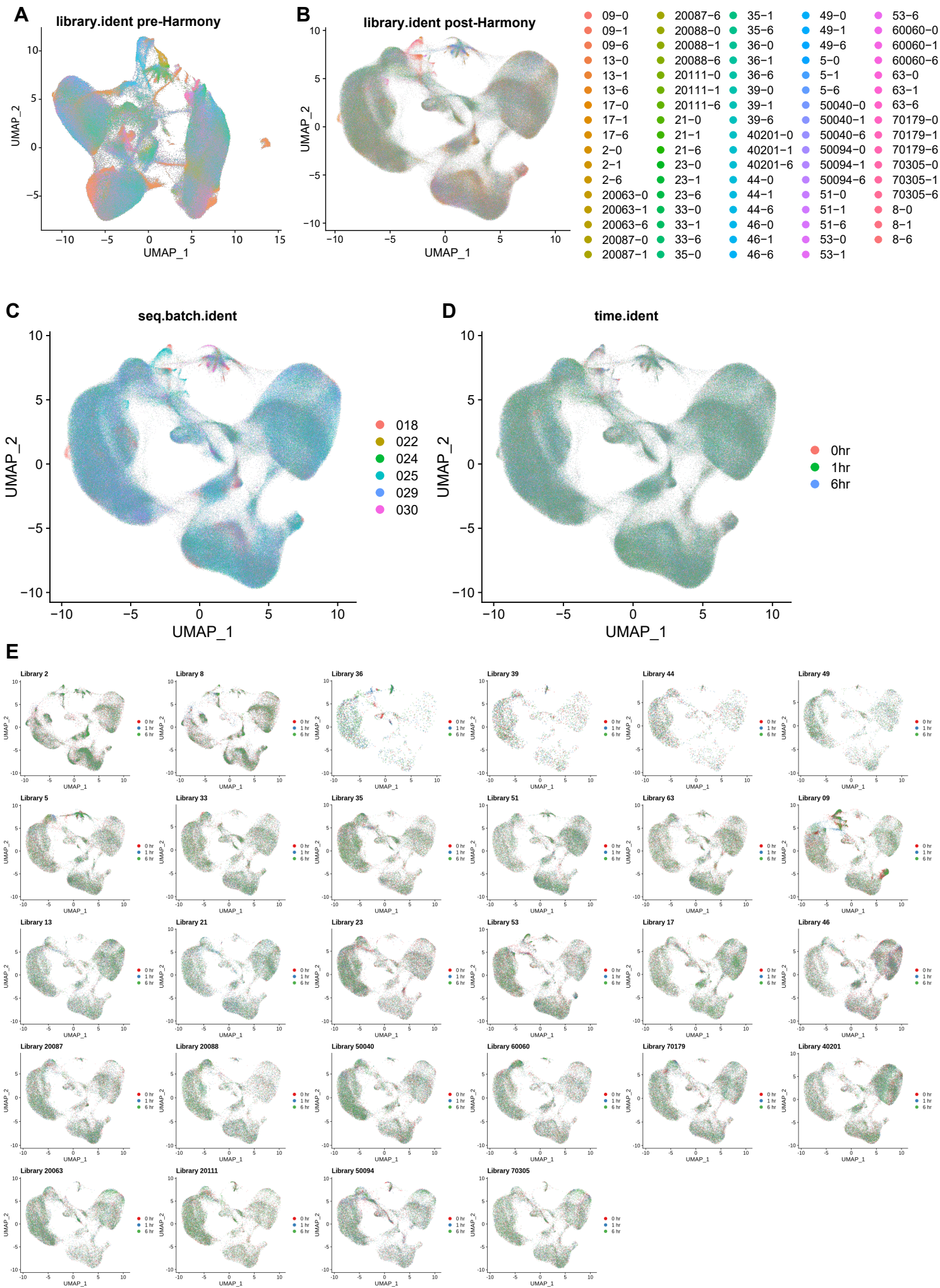

Supplement: Supplement 4 [file media-4.pdf]

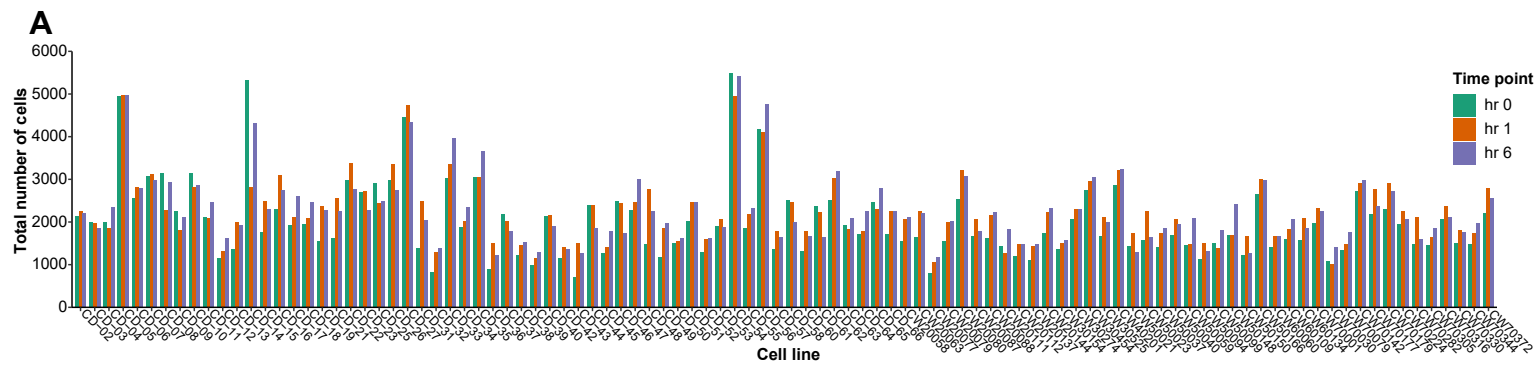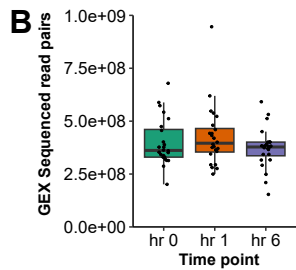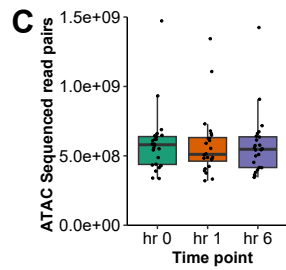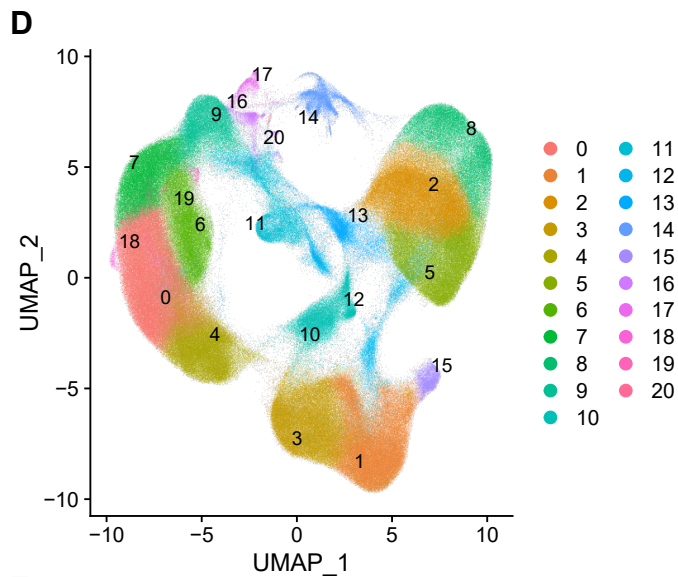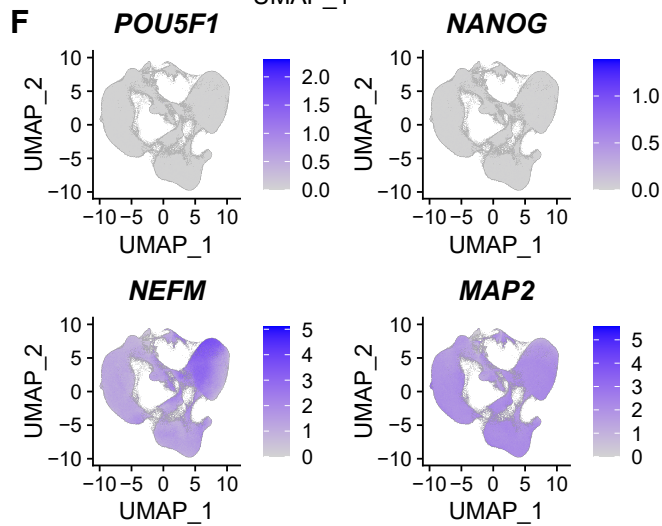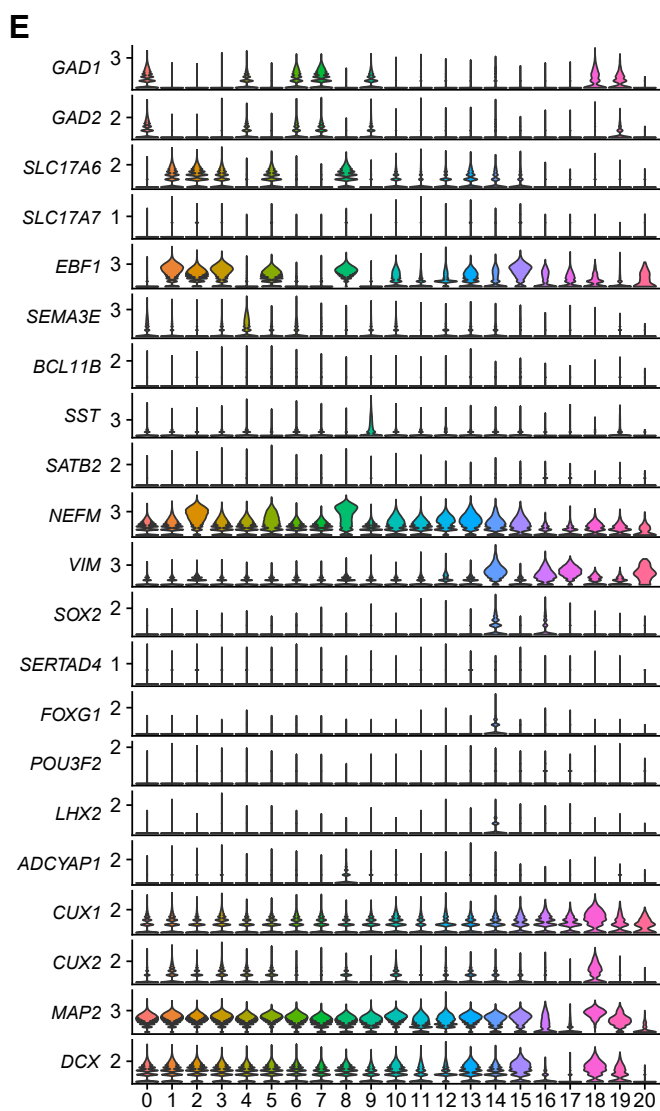

Supplement: Supplement 5 [file media-5.pdf]

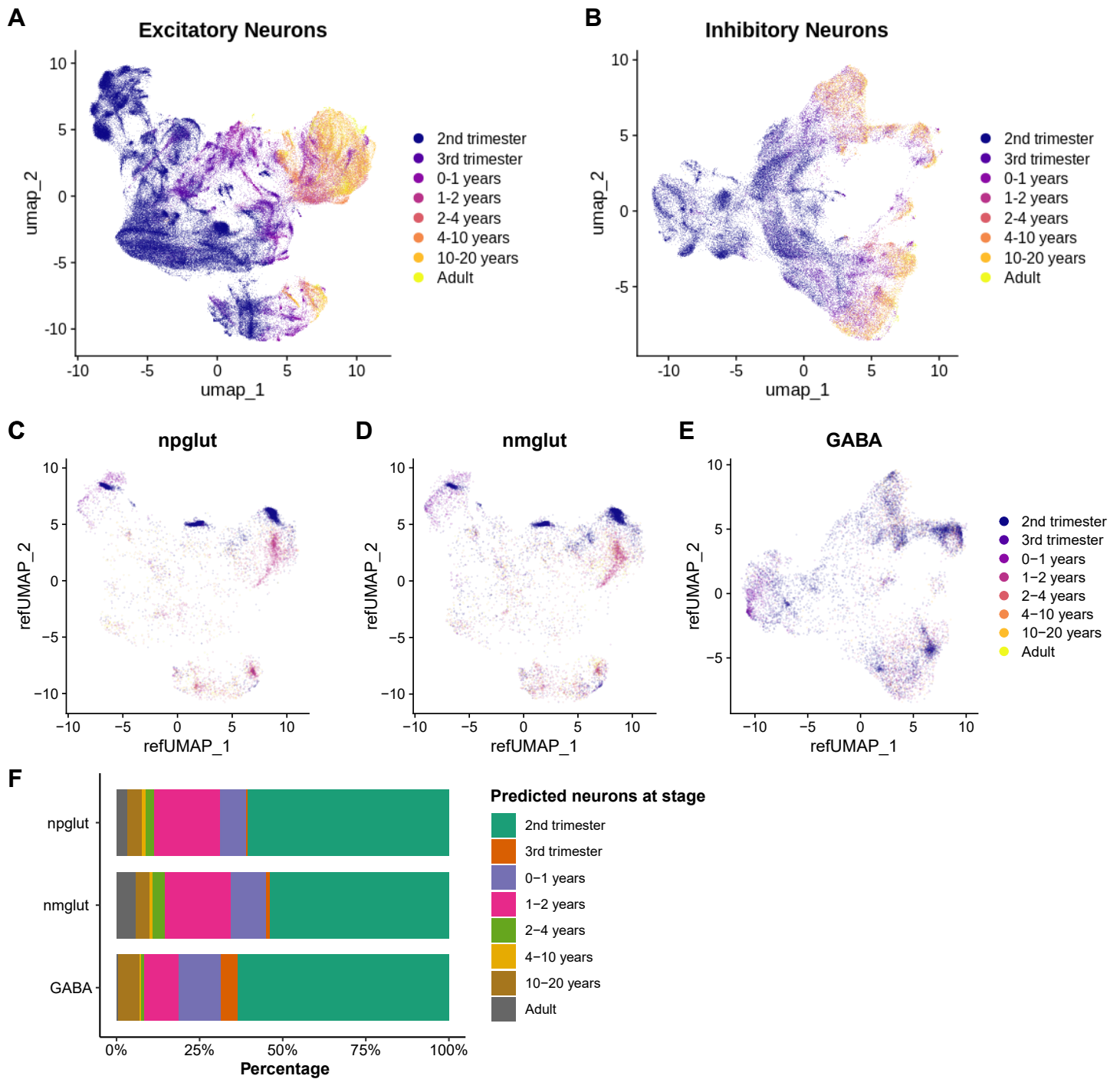

Supplement: Supplement 6 [file media-6.pdf]

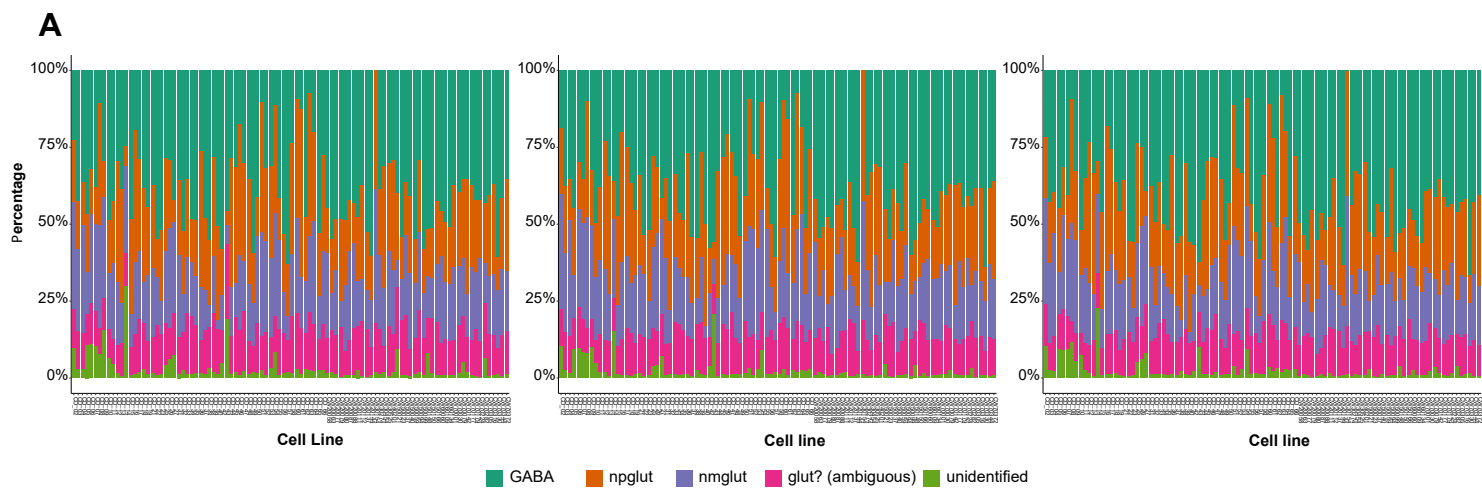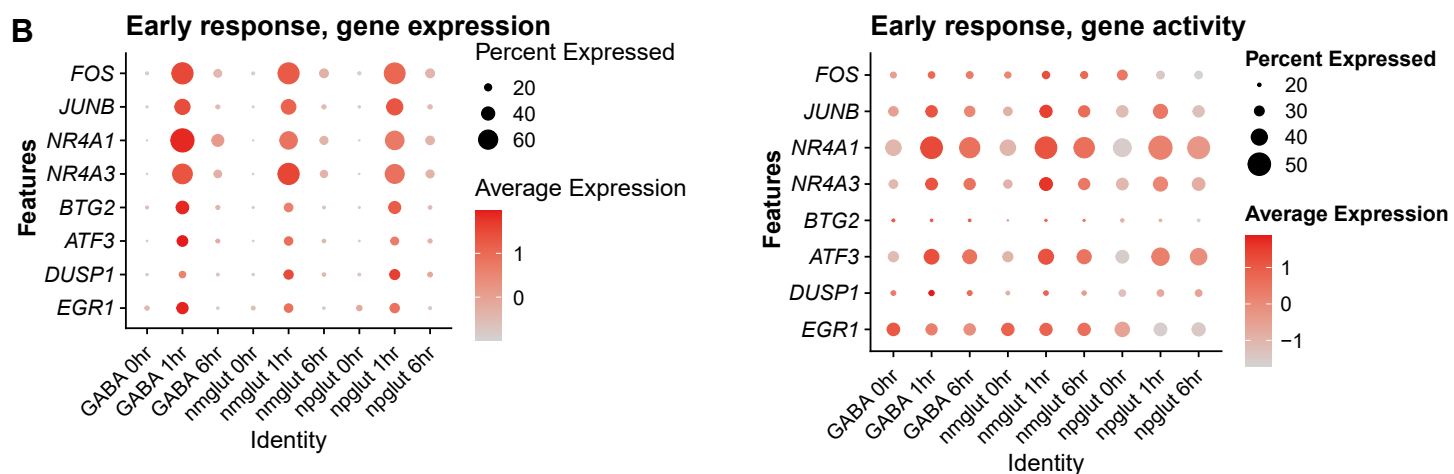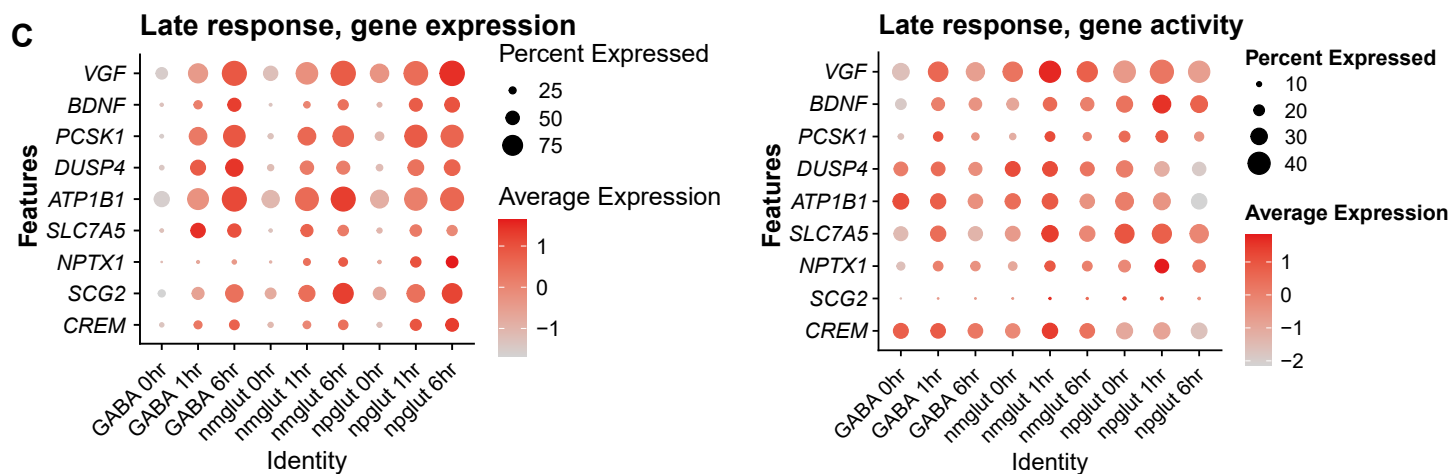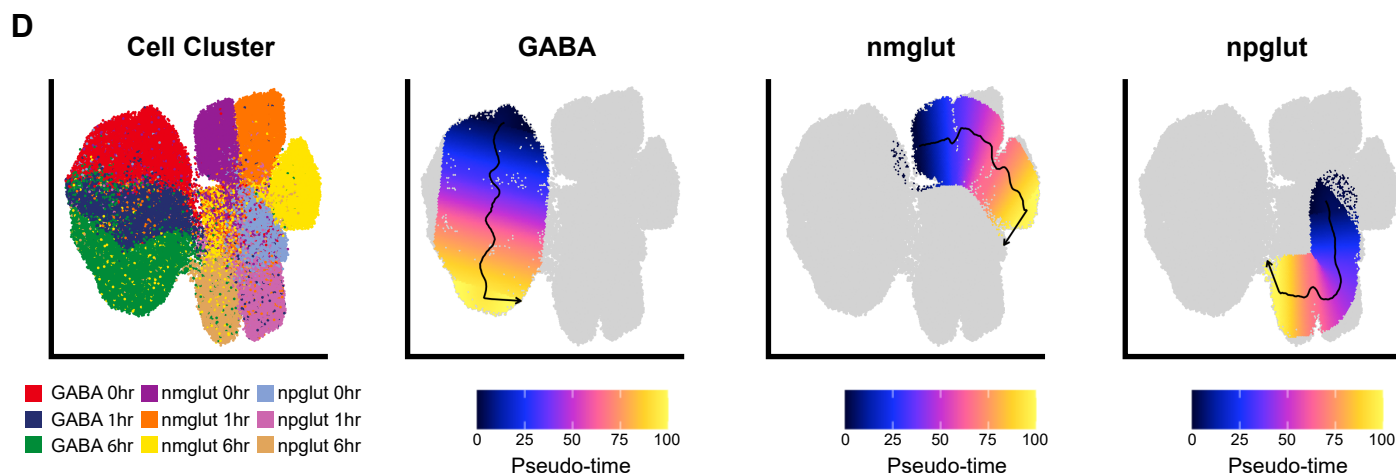

Supplement: Supplement 7 [file media-7.pdf]

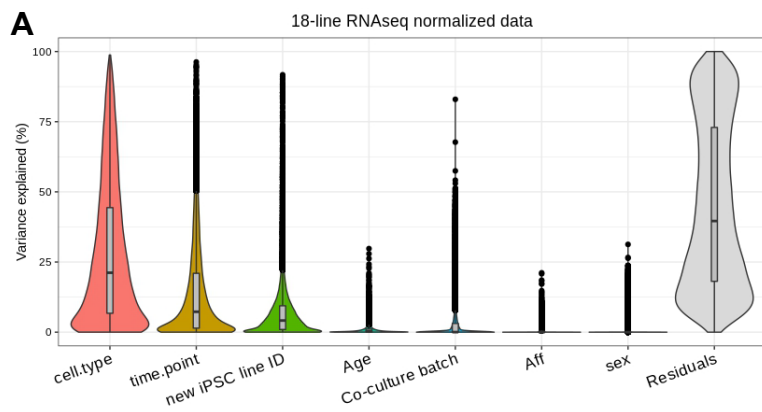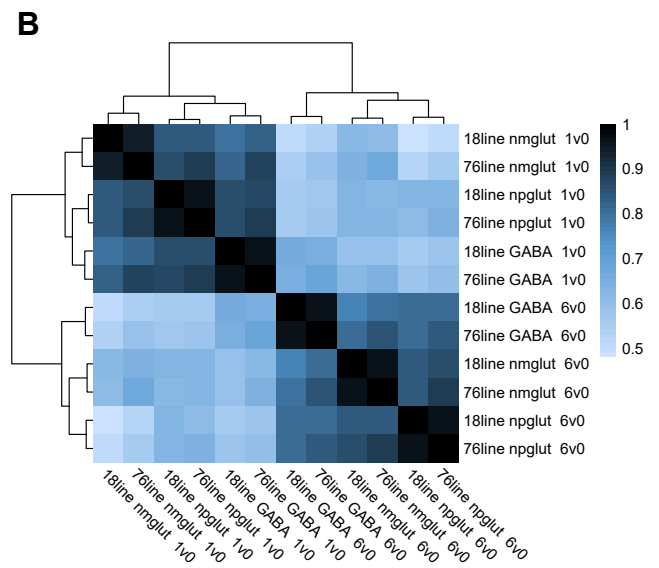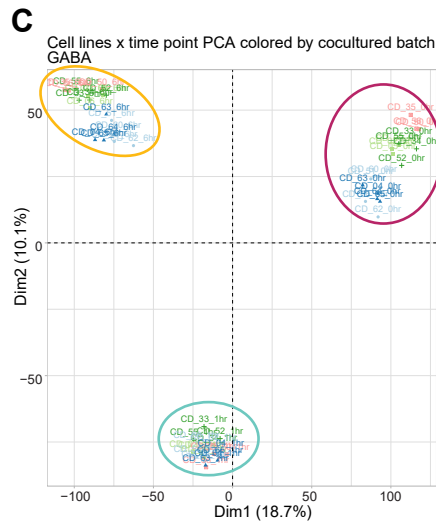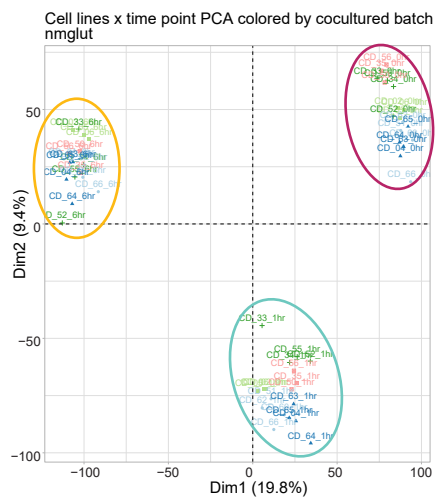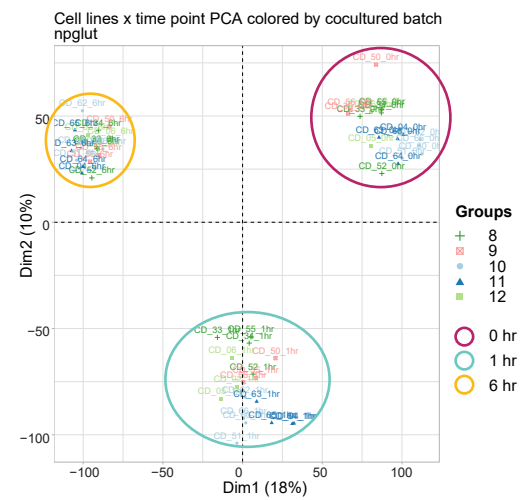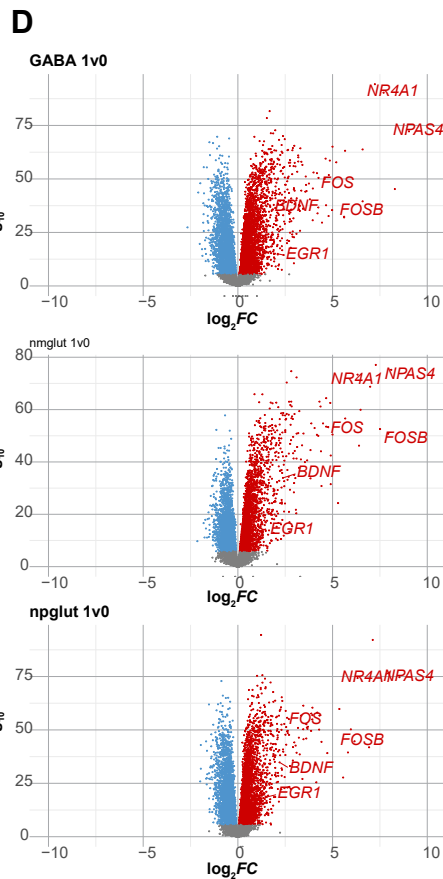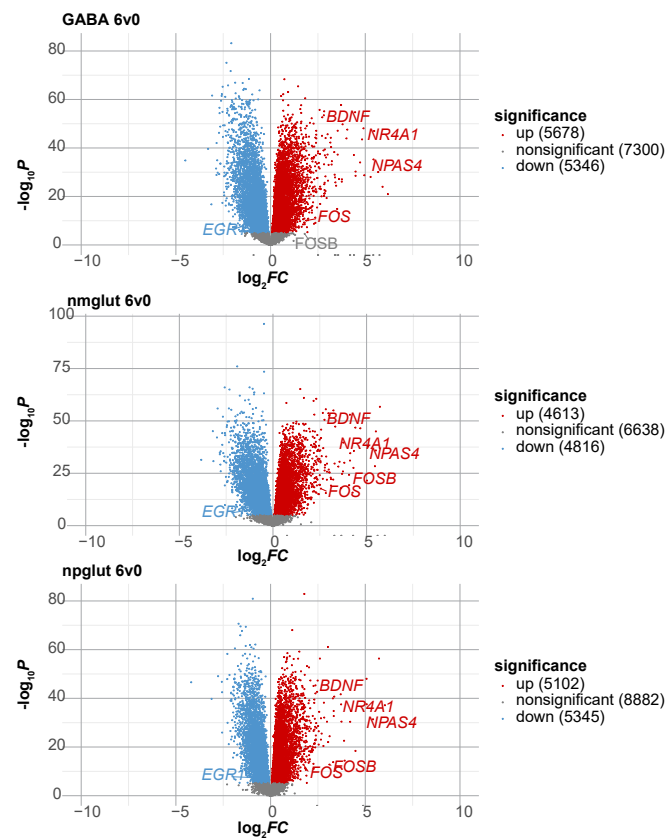

Supplement: Supplement 8 [file media-8.pdf]

A

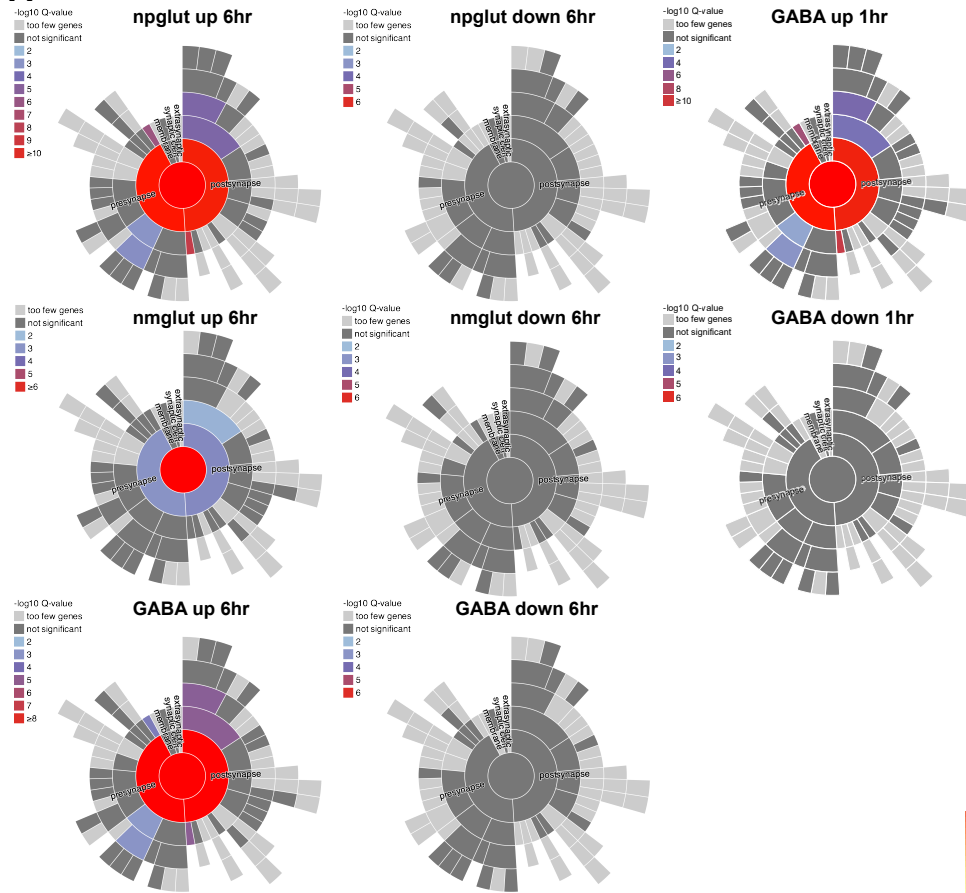

B

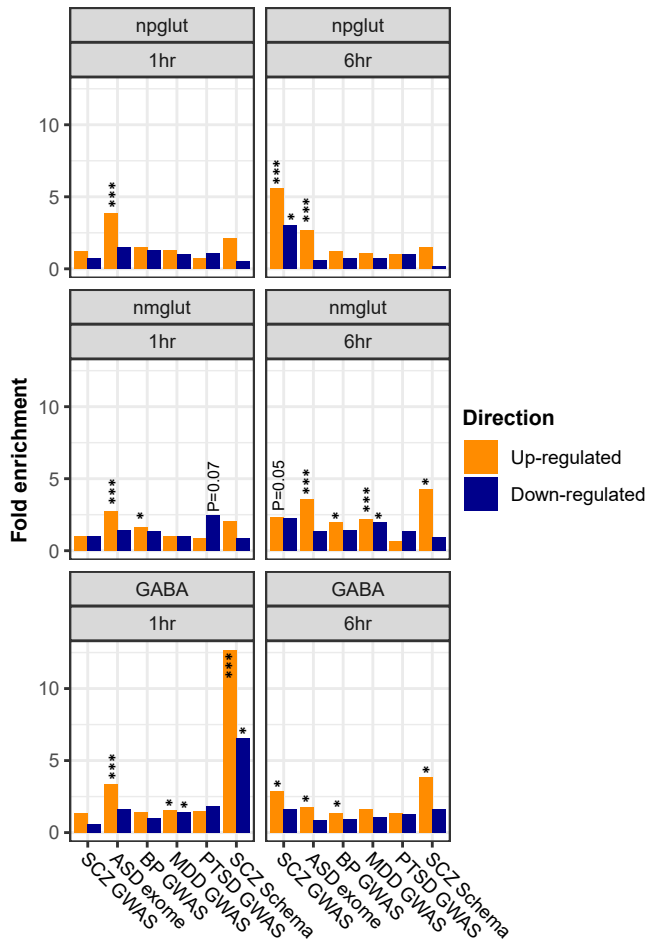

C

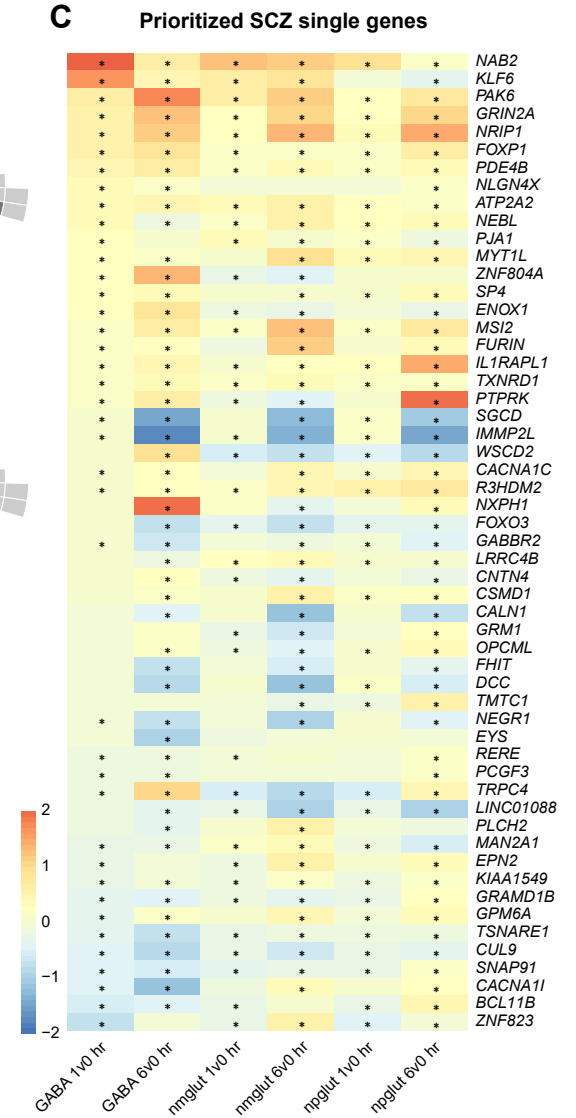

D

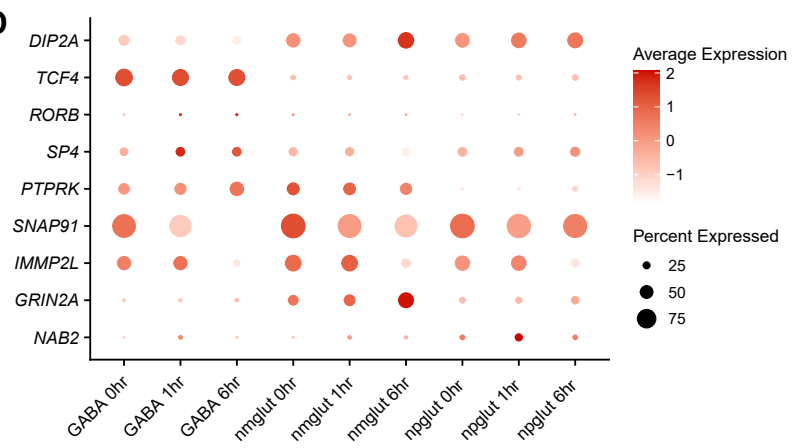

E

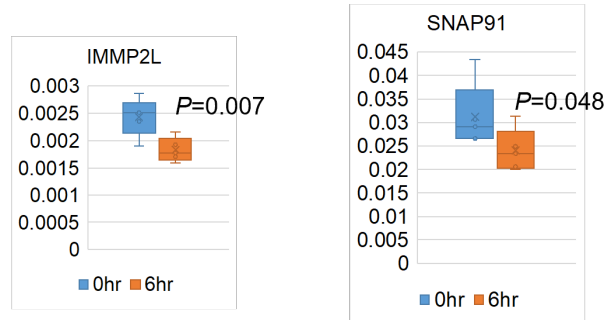

Supplement: Supplement 9 [file media-9.pdf]

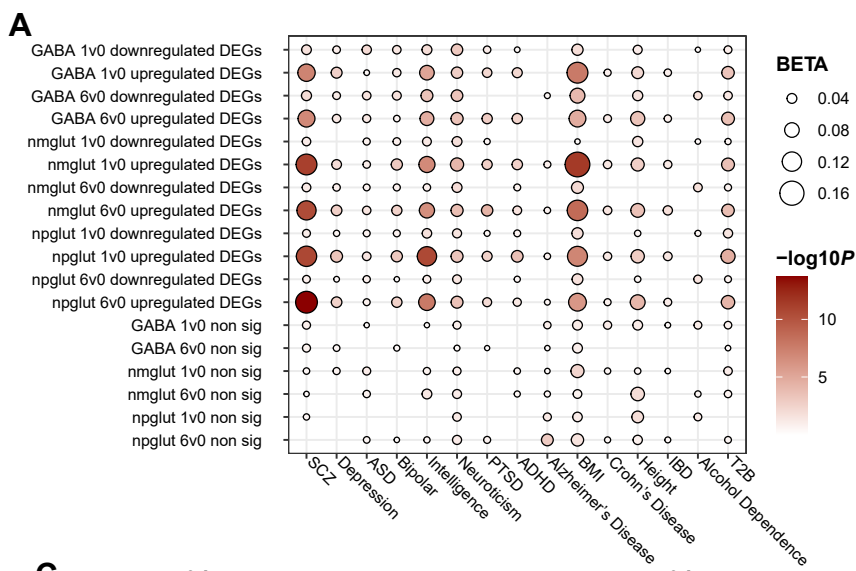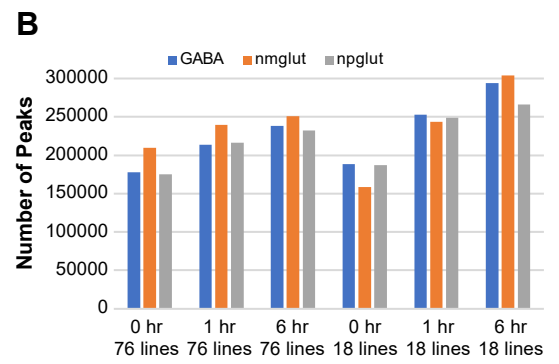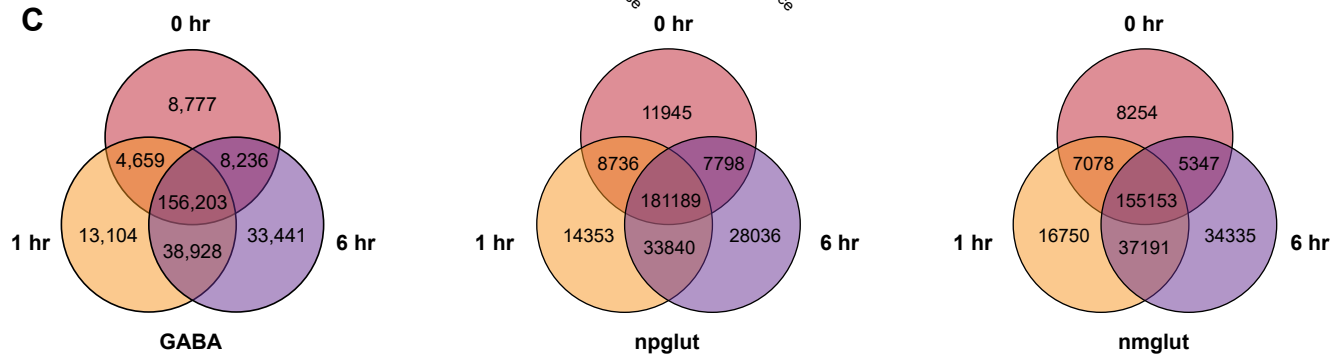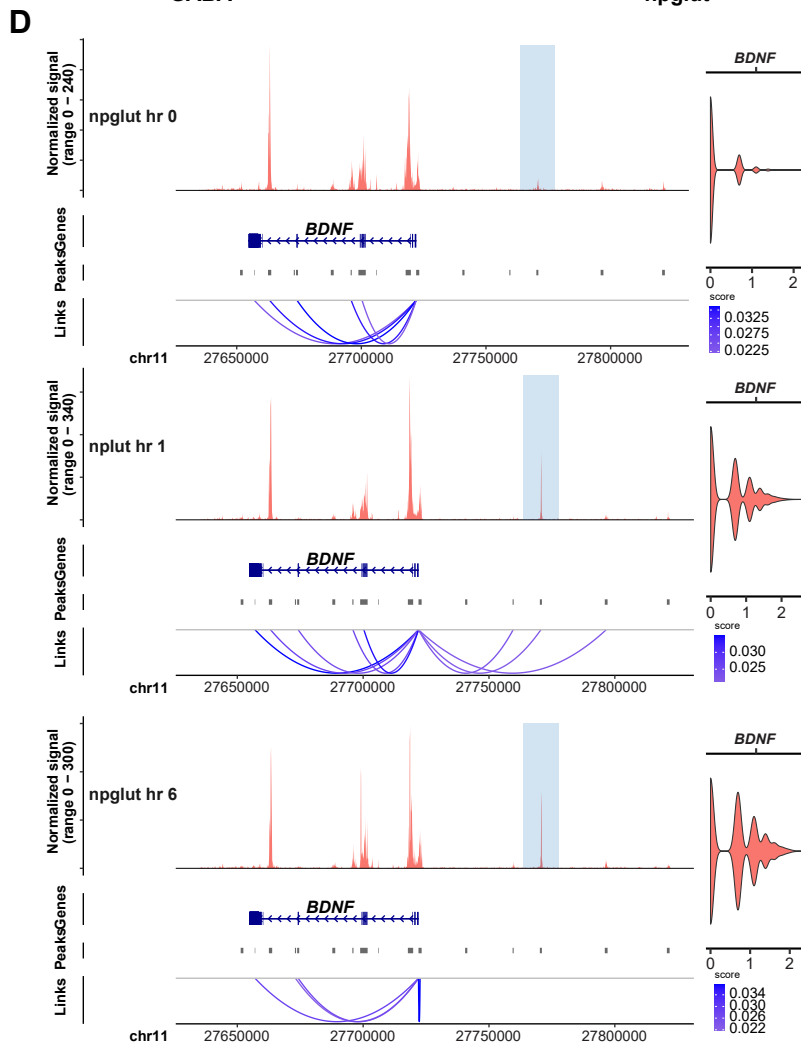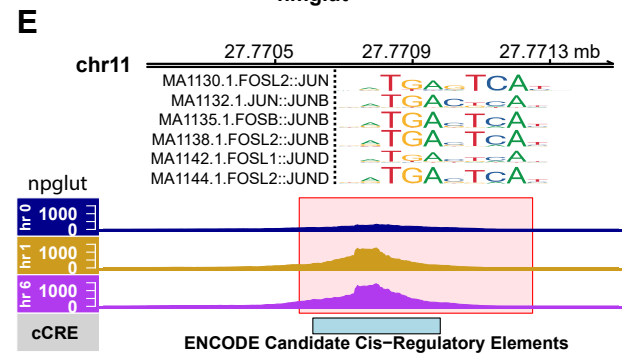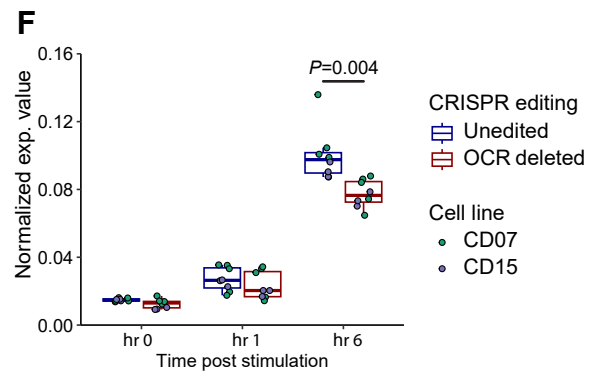

Supplement: Supplement 10 [file media-10.pdf]

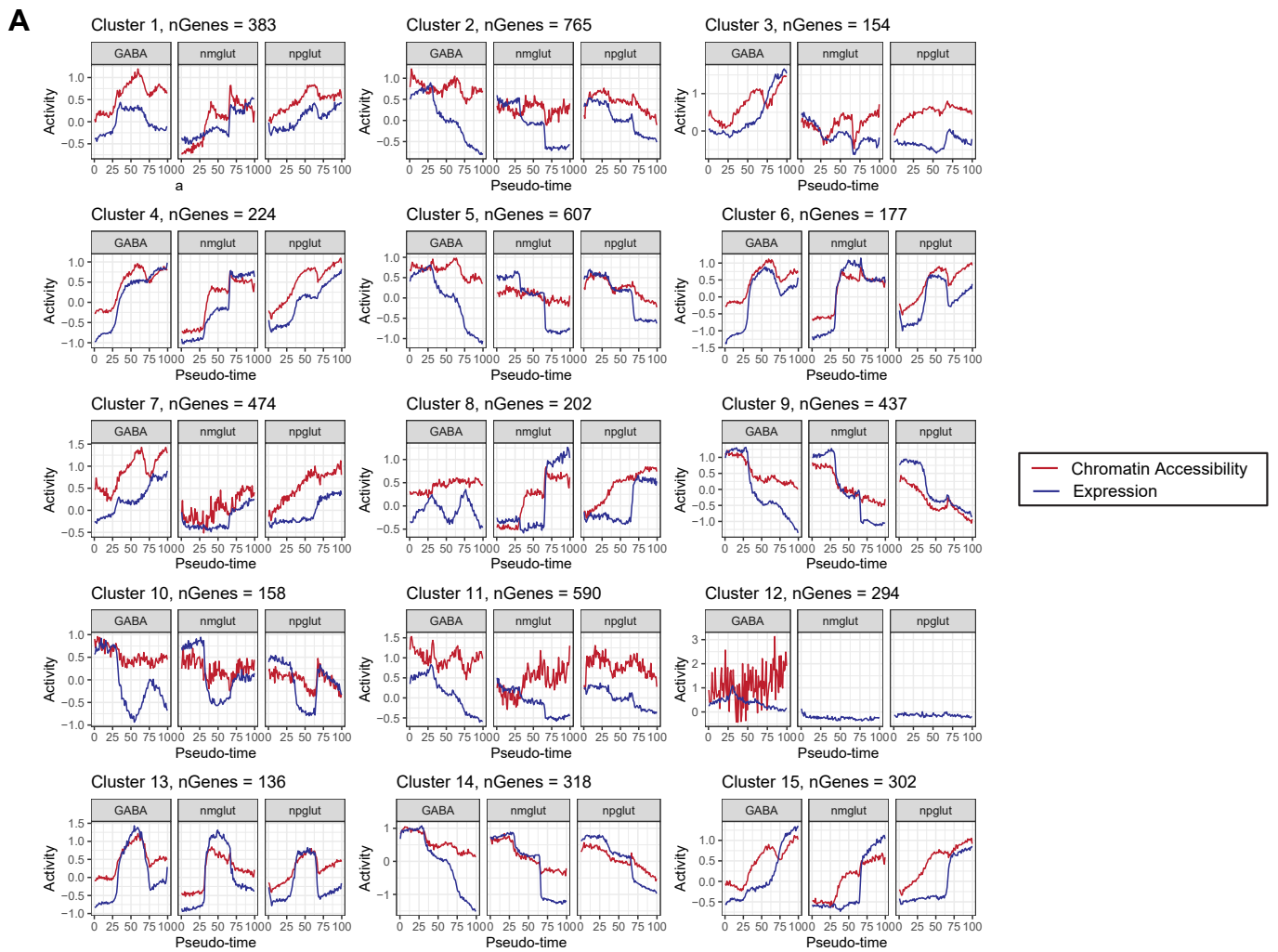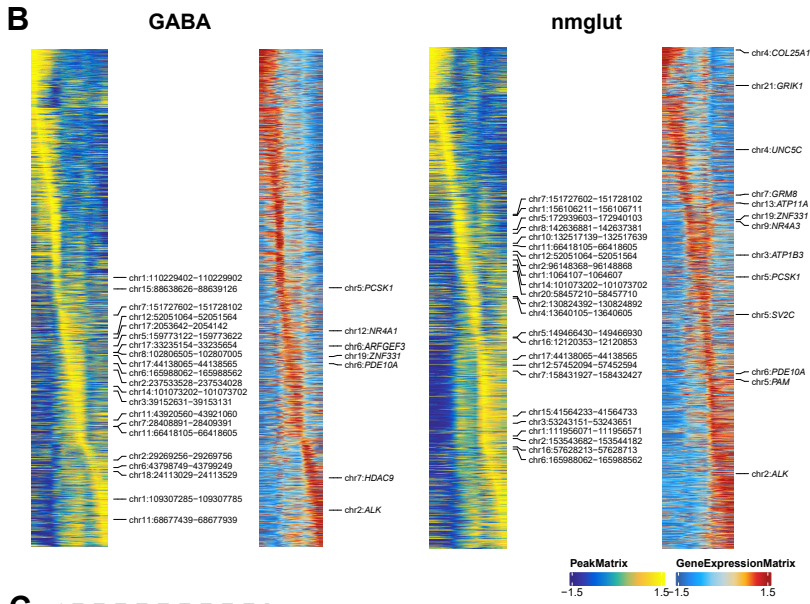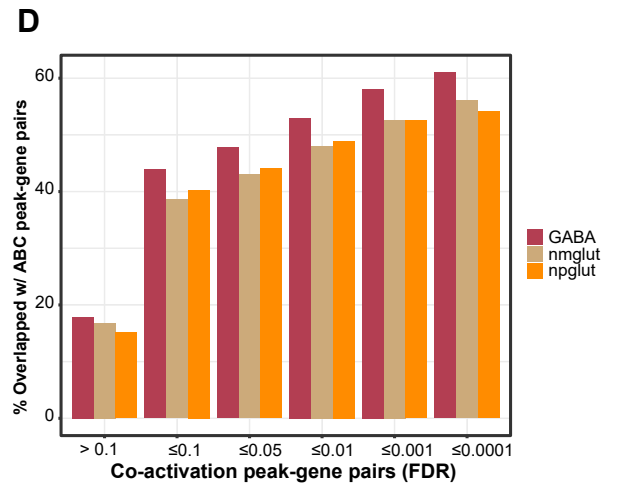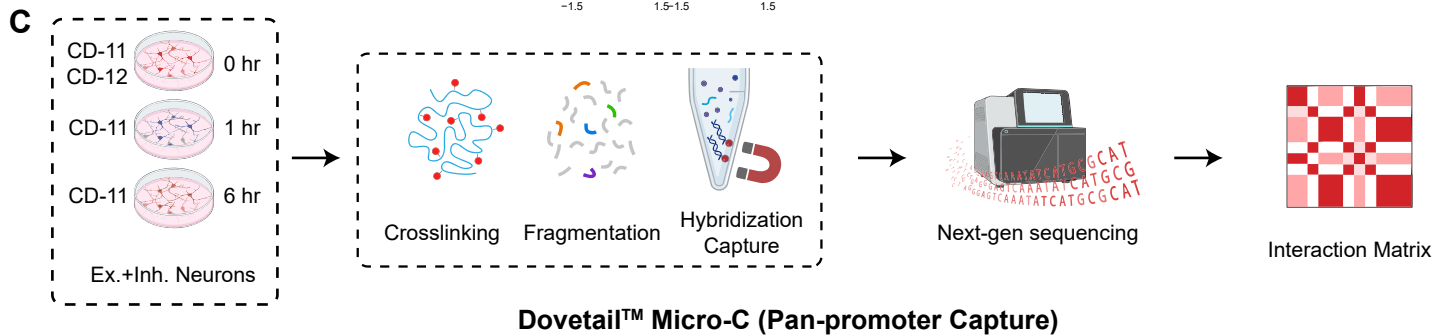

Supplement: Supplement 13 [file media-13.pdf]

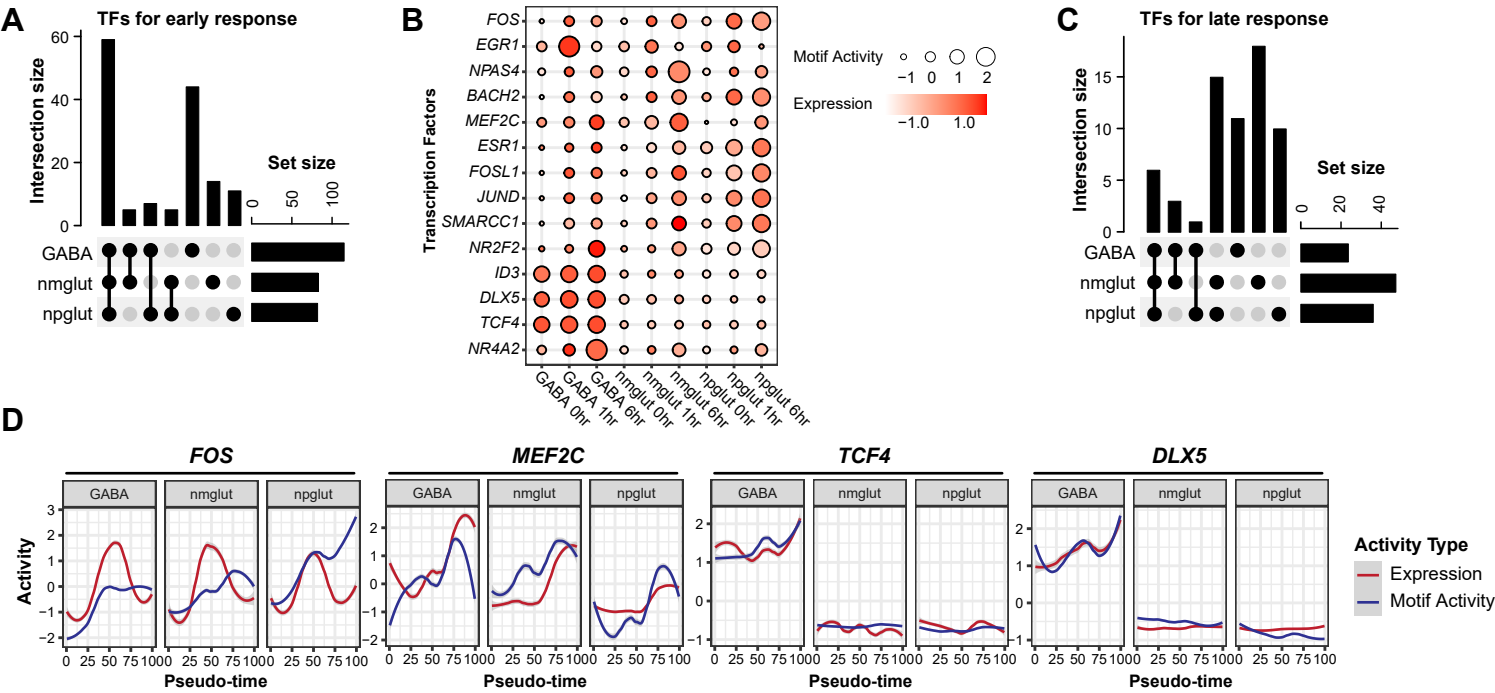

Supplement: Supplement 14 [file media-14.pdf]

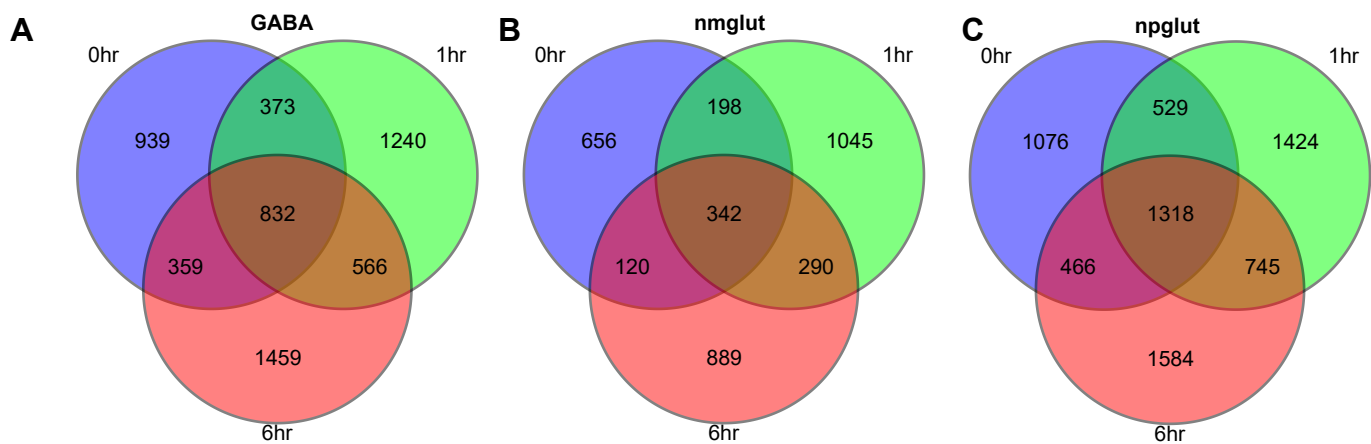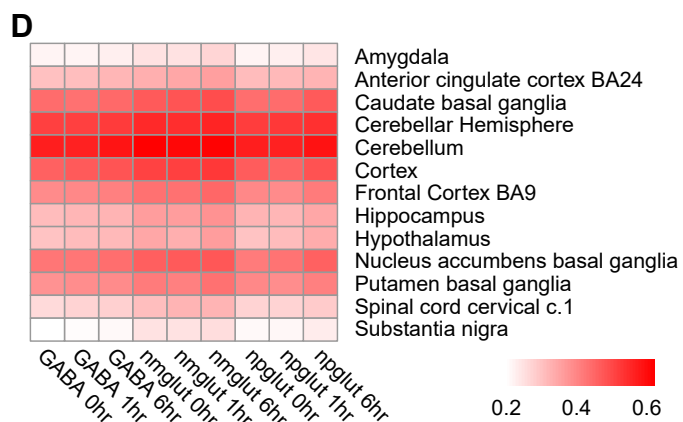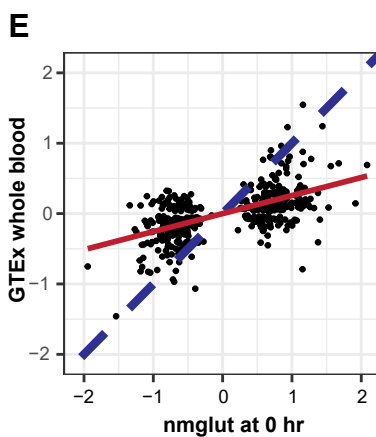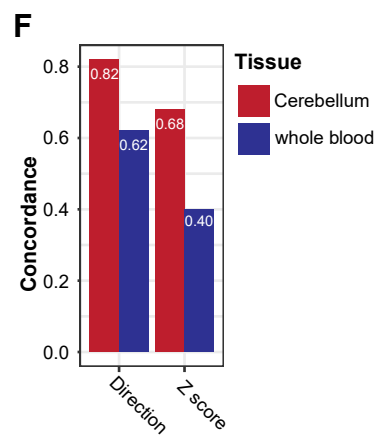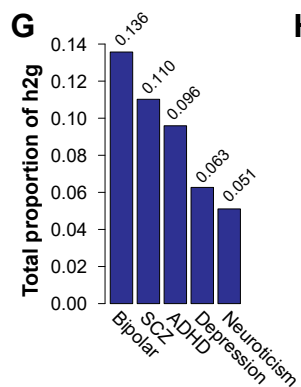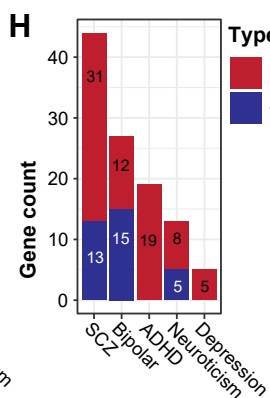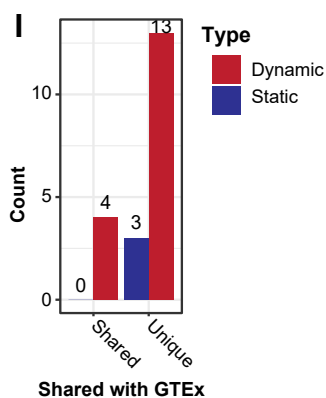

Supplement: Supplement 15 [file media-15.pdf]

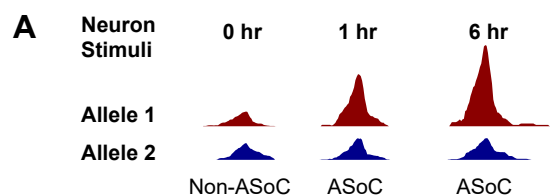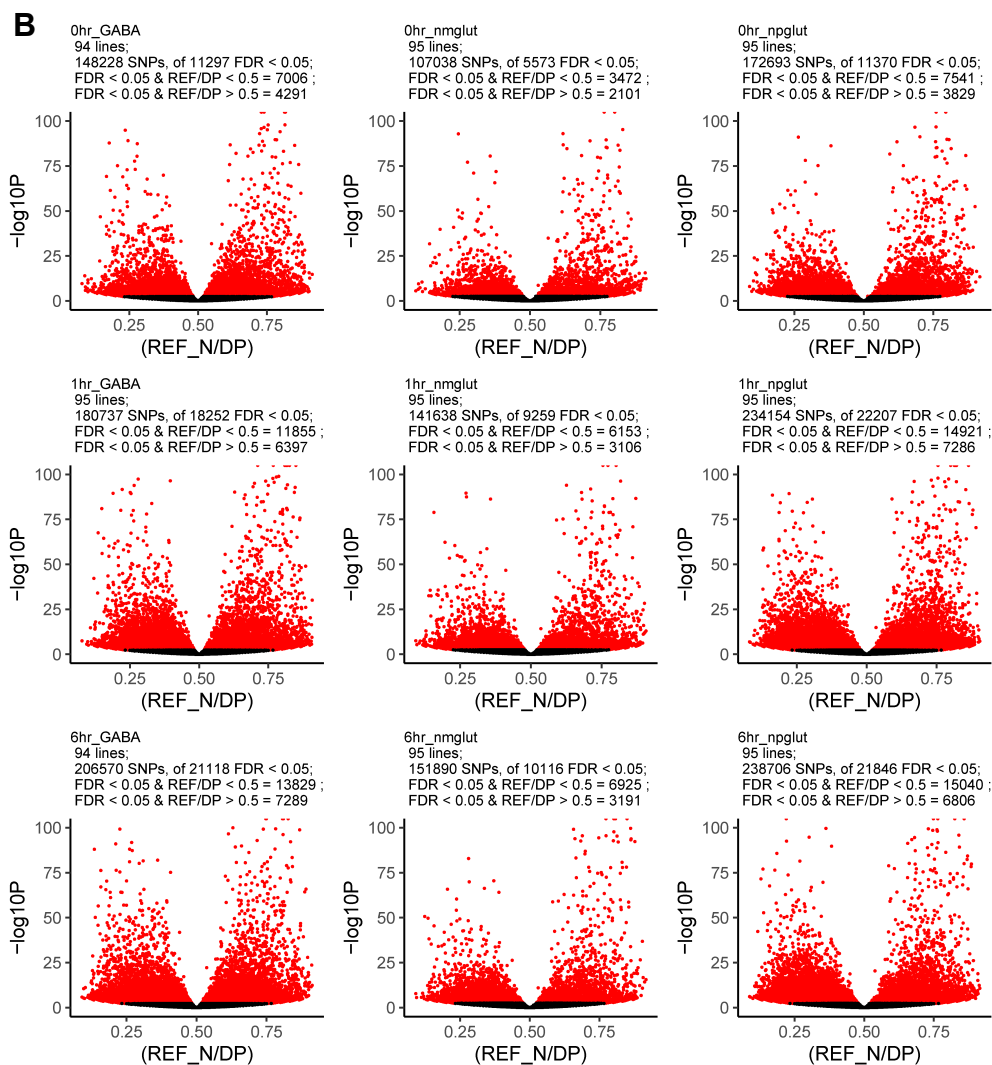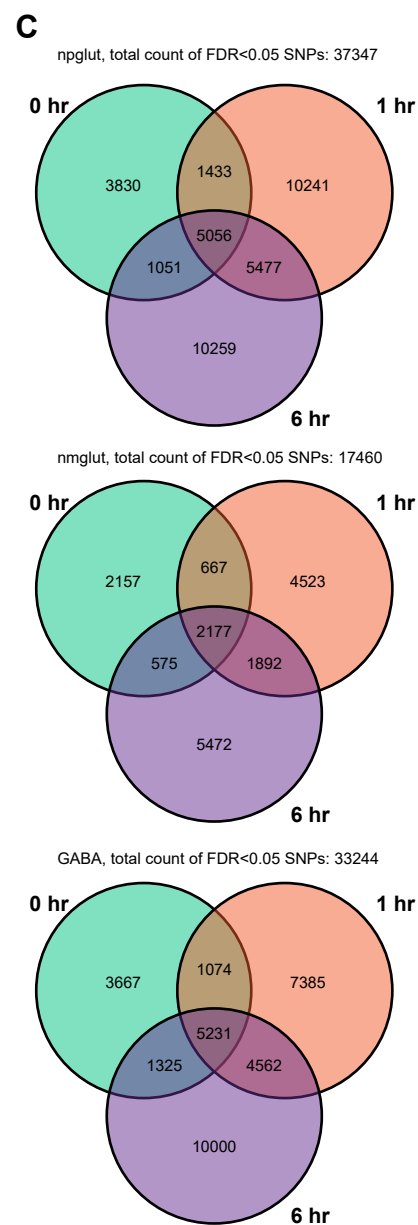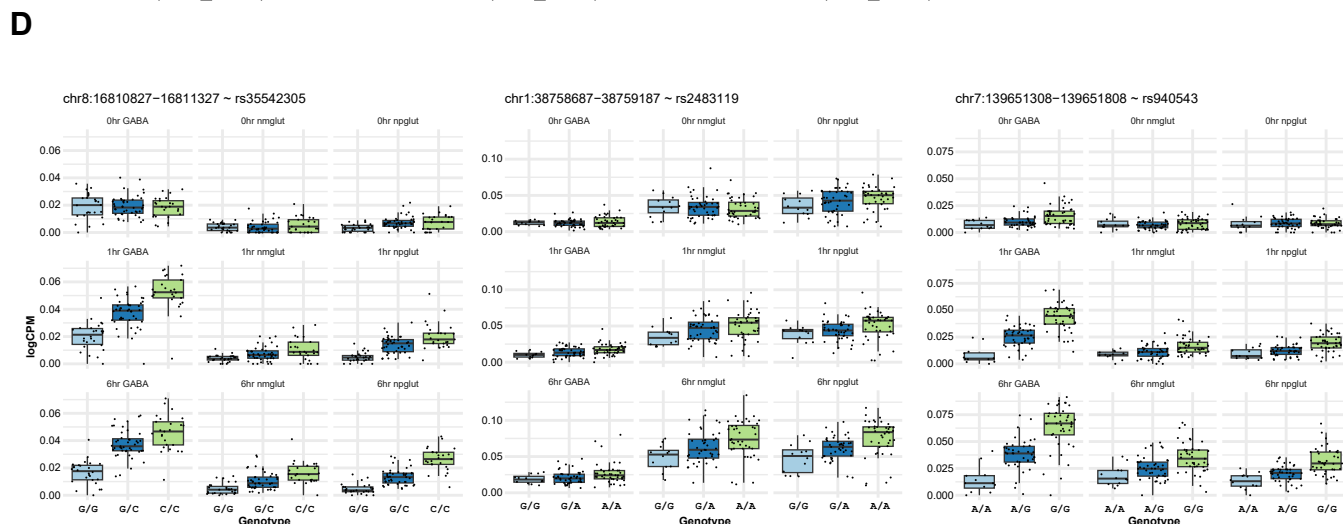

Supplement: Supplement 16 [file media-16.pdf]

**A**

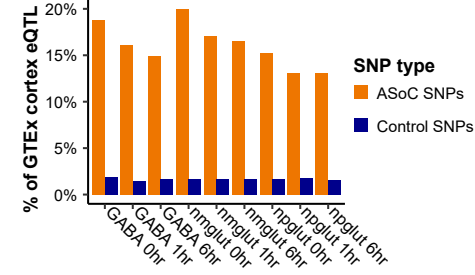

**B**

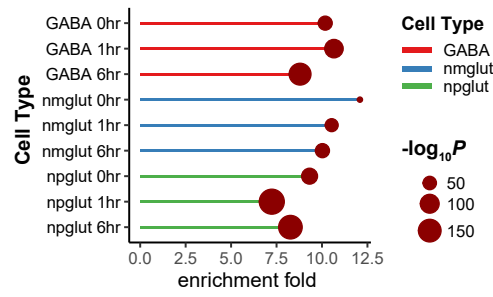

**C**

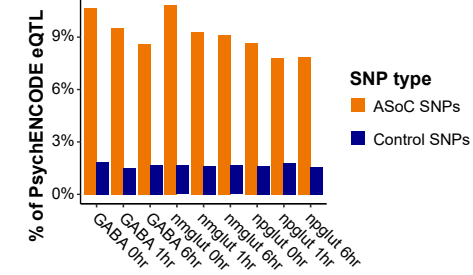

**D**

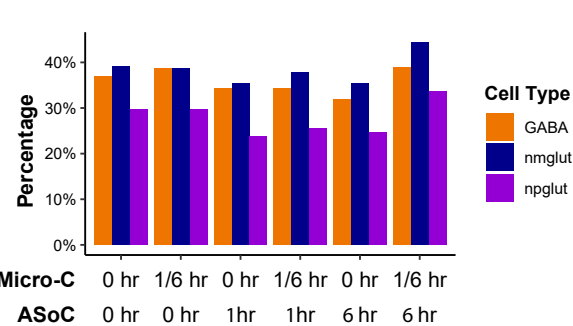

Supplement: Supplement 17 [file media-17.pdf]

**A**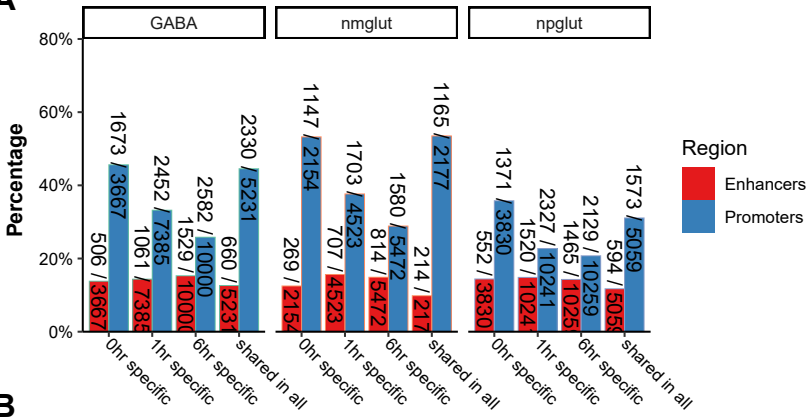**B**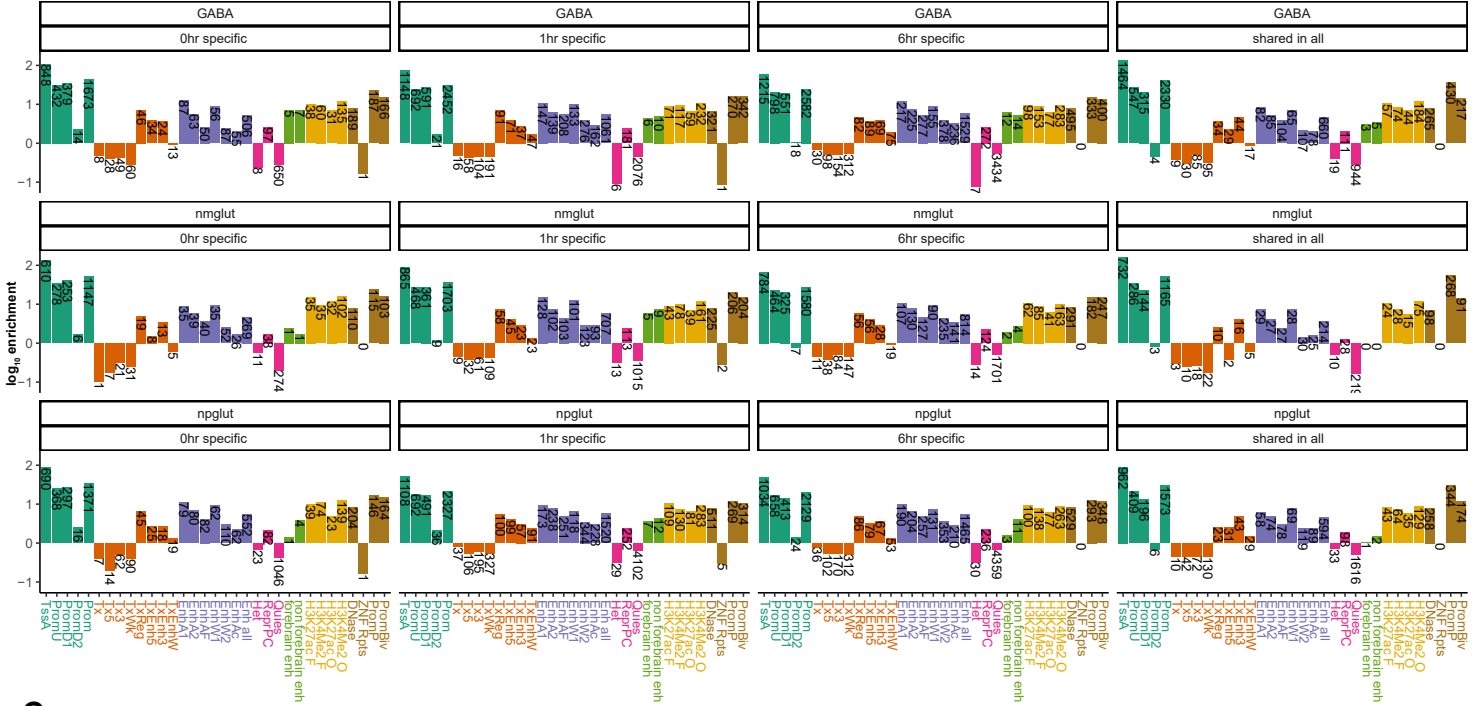**C**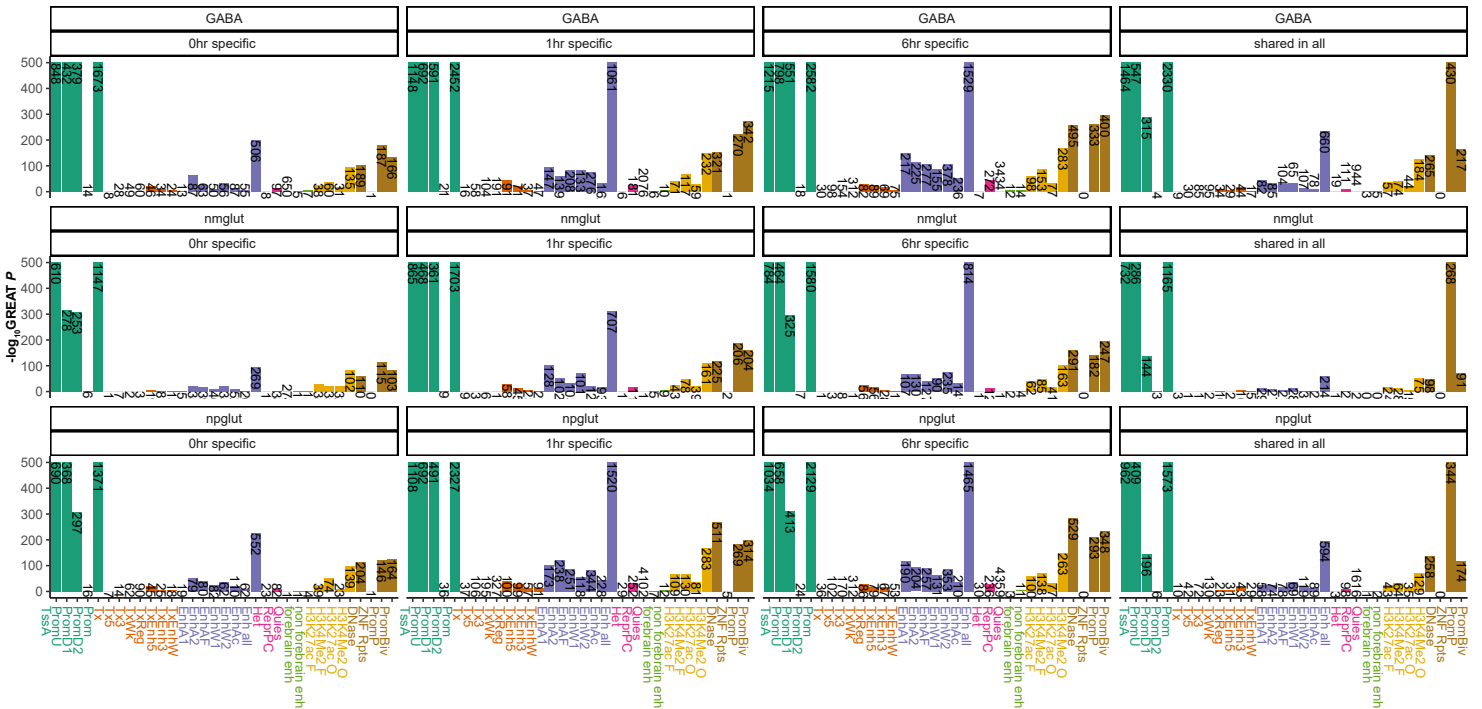

Supplement: Supplement 18 [file media-18.pdf]

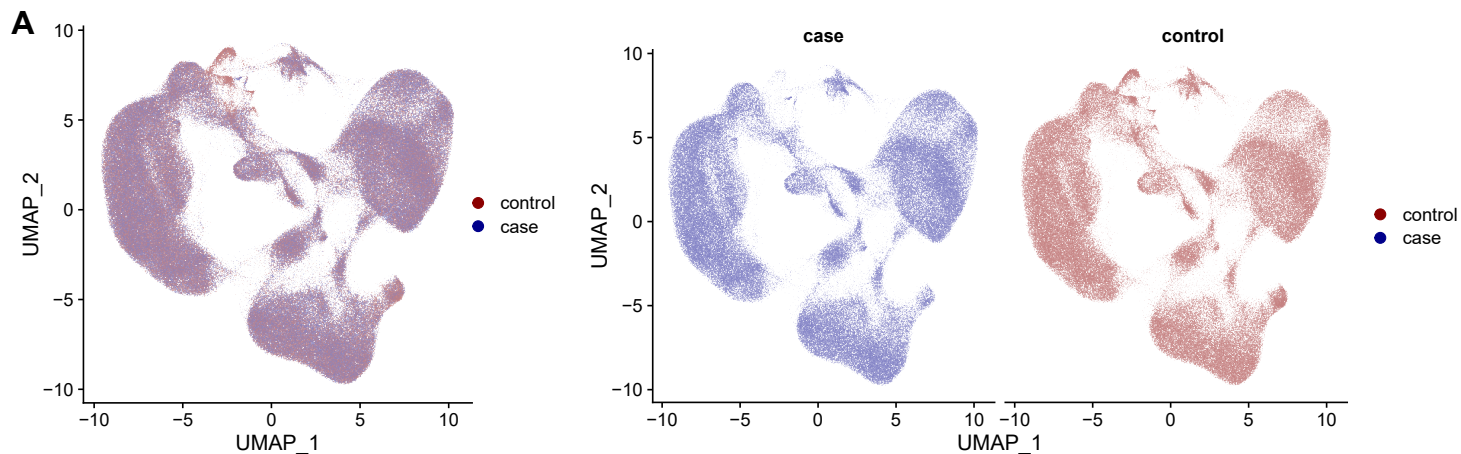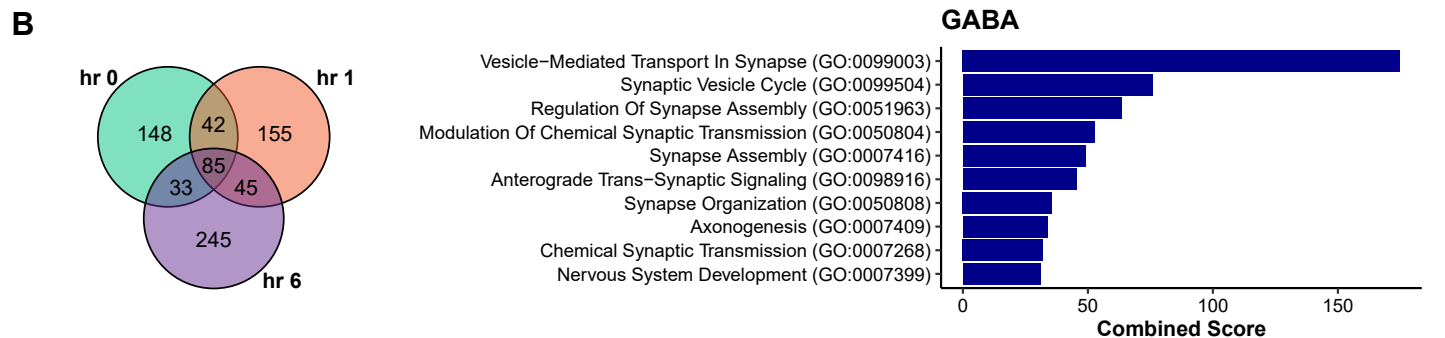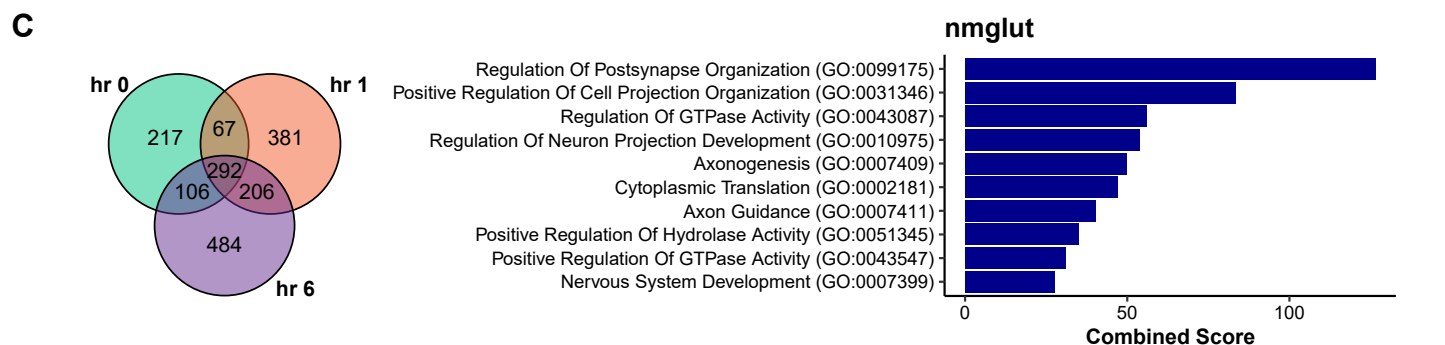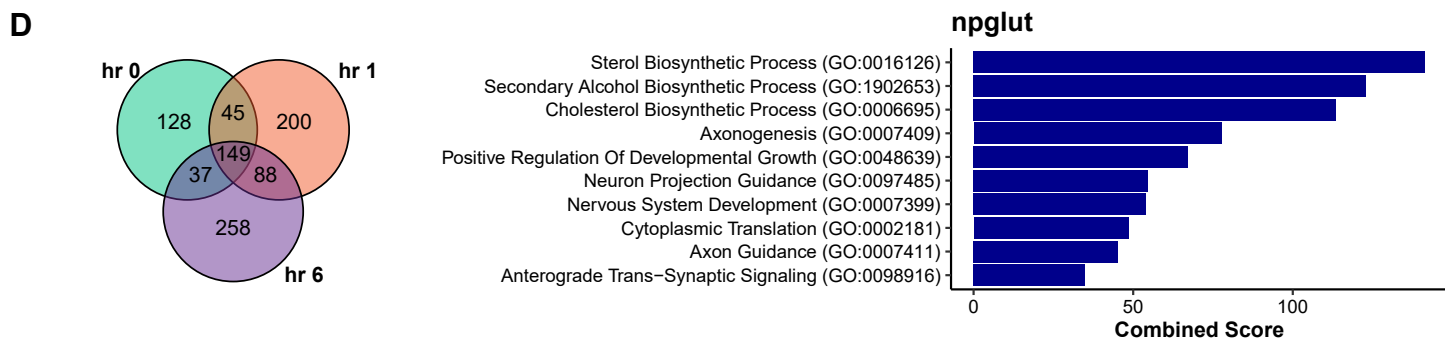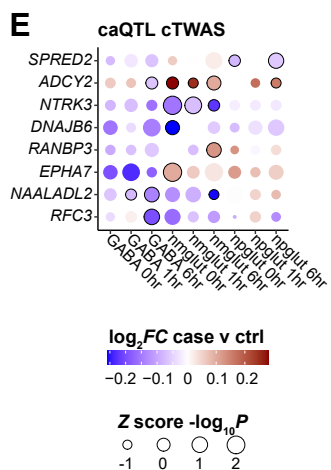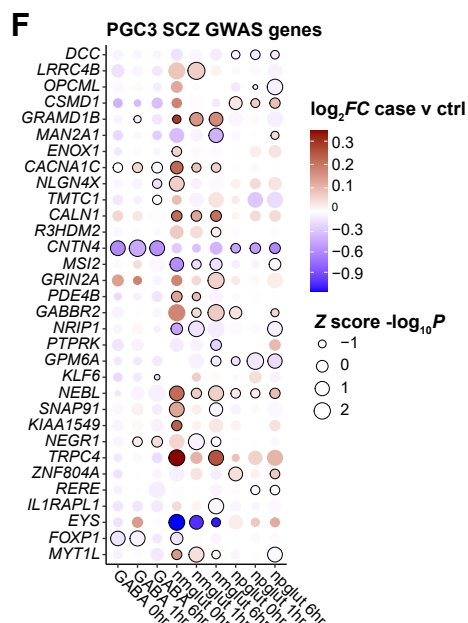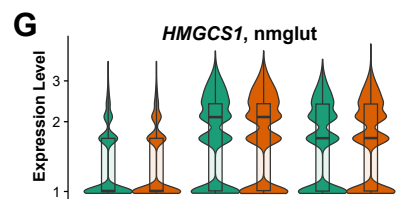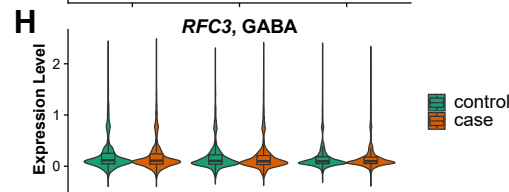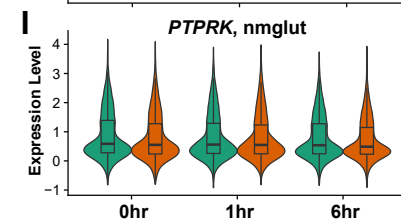

Supplement: Supplement 20 [file media-20.pdf]
