# Supplementary material for: Single-cell multiomics of neuronal activation reveals context-dependent genetic control of brain disorders
Source: bioRxiv. 2025 Feb 17:2025.02.17.638682. Preprint. [Version 1] doi: 10.1101/2025.02.17.638682 (PMC11870544; doi:10.1101/2025.02.17.638682)
Supplement: Supplement 11 [file media-11.pdf]

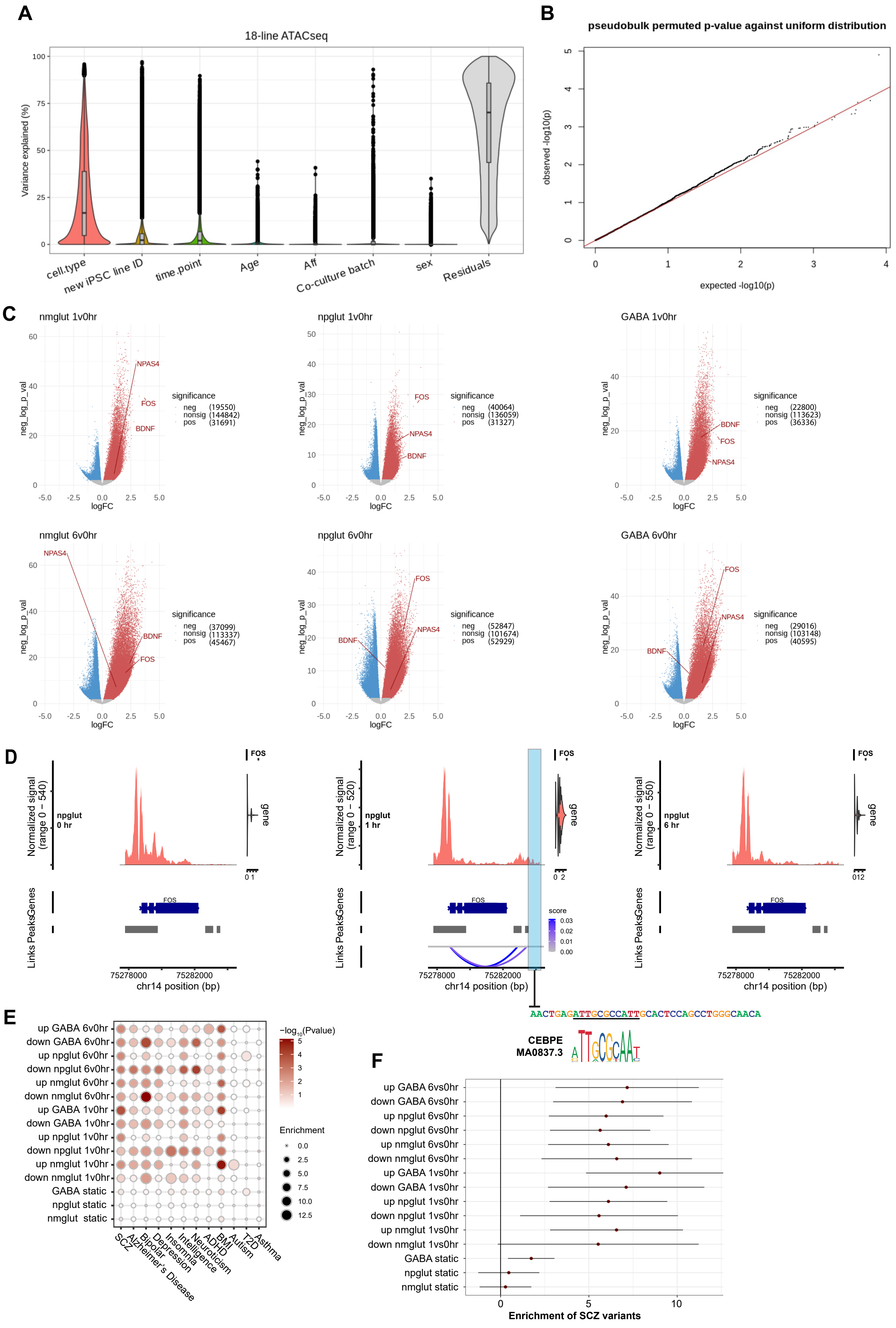

A
18-line ATACseq
Variance explained (%)
cell.type
new iPSC line ID
time.point
Age
Aff
Co-culture batch
sex
Residuals
B
pseudobulk permuted p-value against uniform distribution
observed -log10(p)
expected -log10(p)
C
nmglut 1v0hr
npglut 1v0hr
GABA 1v0hr
nmglut 6v0hr
npglut 6v0hr
GABA 6v0hr
neg_log_p_val
logFC
significance
neg (19550)
nonsig (144842)
pos (31691)
neg (40064)
nonsig (136059)
pos (31327)
neg (22800)
nonsig (113623)
pos (36336)
neg (37099)
nonsig (113337)
pos (45467)
neg (52847)
nonsig (101674)
pos (52929)
neg (29016)
nonsig (103148)
pos (40595)
NPAS4
FOS
BDNF
D
Normalized signal (range 0 – 540)
Normalized signal (range 0 – 520)
Normalized signal (range 0 – 550)
npglut 0 hr
npglut 1 hr
npglut 6 hr
gene
Links PeaksGenes
score
chr14 position (bp)
75278000
75282000
AACTGAGATTGCGCCATTGCACTCCAGCCTGGGCAACA
CEBPE MA0837.3
E
up GABA 6v0hr
down GABA 6v0hr
up npglut 6v0hr
down npglut 6v0hr
up nmglut 6v0hr
down nmglut 6v0hr
up GABA 1v0hr
down GABA 1v0hr
up npglut 1v0hr
down npglut 1v0hr
up nmglut 1v0hr
down nmglut 1v0hr
GABA static
npglut static
nmglut static
SCZ
Alzheimer's Disease
Bipolar
Depression
Insomnia
Intelligence
Neuroticism
ADHD
BMI
Autism
T2D
Asthma
−log10(Pvalue)
Enrichment
F
up GABA 6vs0hr
down GABA 6vs0hr
up npglut 6vs0hr
down npglut 6vs0hr
up nmglut 6vs0hr
down nmglut 6vs0hr
up GABA 1vs0hr
down GABA 1vs0hr
up npglut 1vs0hr
down npglut 1vs0hr
up nmglut 1vs0hr
down nmglut 1vs0hr
GABA static
npglut static
nmglut static
Enrichment of SCZ variants
