# Supplementary material for: Single-cell multiomics of neuronal activation reveals context-dependent genetic control of brain disorders
Source: bioRxiv. 2025 Feb 17:2025.02.17.638682. Preprint. [Version 1] doi: 10.1101/2025.02.17.638682 (PMC11870544; doi:10.1101/2025.02.17.638682)
Supplement: Supplement 12 [file media-12.pdf]

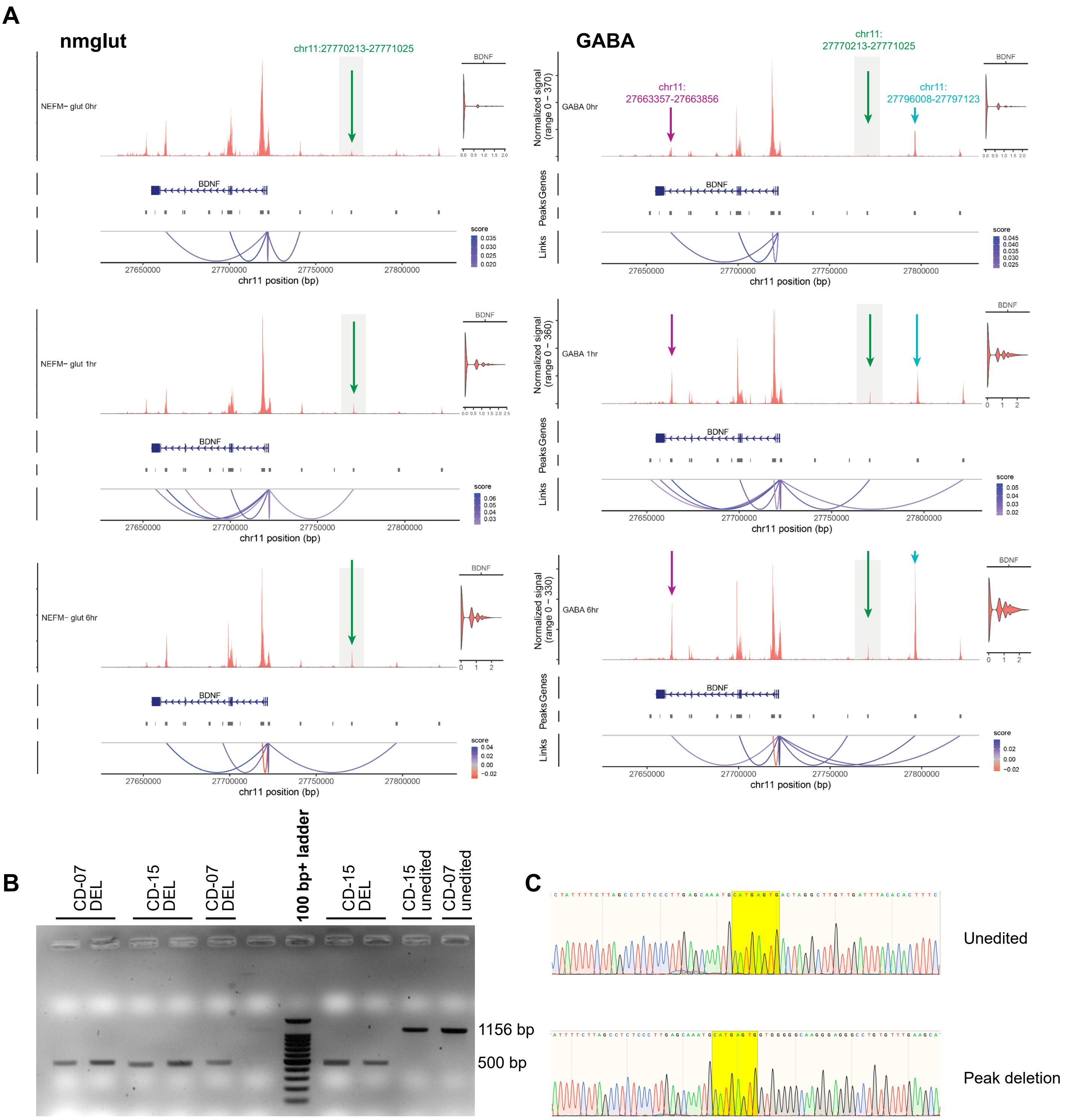

A
nmglut
GABA
chr11:27770213-27771025
chr11:
27663357-27663856
chr11:
27796008-27797123
NEFM− glut 0hr
NEFM− glut 1hr
NEFM− glut 6hr
GABA 0hr
GABA 1hr
GABA 6hr
Normalized signal (range 0 − 370)
Normalized signal (range 0 − 360)
Normalized signal (range 0 − 330)
BDNF
Genes
Peaks
Links
score
chr11 position (bp)
27650000
27700000
27750000
27800000
B
CD-07 DEL
CD-15 DEL
CD-07 DEL
100 bp+ ladder
CD-15 DEL
CD-15 unedited
CD-07 unedited
1156 bp
500 bp
C
Unedited
Peak deletion
