# Supplementary material for: Single-cell multiomics of neuronal activation reveals context-dependent genetic control of brain disorders
Source: bioRxiv. 2025 Feb 17:2025.02.17.638682. Preprint. [Version 1] doi: 10.1101/2025.02.17.638682 (PMC11870544; doi:10.1101/2025.02.17.638682)
Supplement: Supplement 19 [file media-19.pdf]

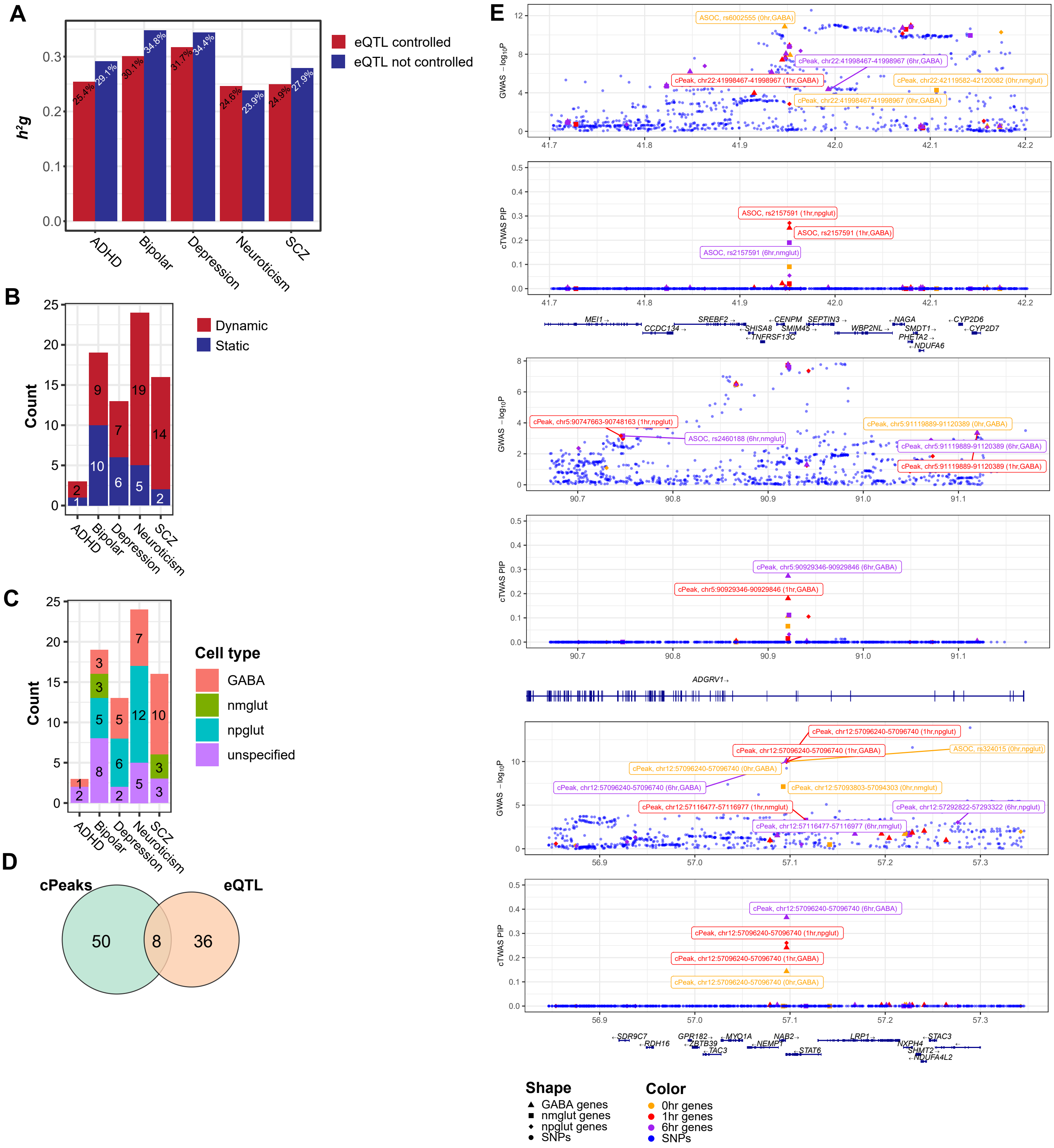
A
eQTL controlled
eQTL not controlled
h²g
0.0
0.1
0.2
0.3
25.4%
29.1%
30.1%
34.8%
31.7%
34.4%
24.6%
23.9%
24.9%
27.9%
ADHD
Bipolar
Depression
Neuroticism
SCZ
B
Dynamic
Static
Count
0
5
10
15
20
25
2
1
9
10
7
6
19
5
14
2
ADHD
Bipolar
Depression
Neuroticism
SCZ
C
Cell type
GABA
nmglut
npglut
unspecified
Count
1
2
3
3
5
8
5
6
2
7
12
5
10
3
3
ADHD
Bipolar
Depression
Neuroticism
SCZ
D
cPeaks
eQTL
50
8
36
E
GWAS −log10P
cTWAS PIP
ASOC, rs6002555 (0hr,GABA)
cPeak, chr22:41998467-41998967 (6hr,GABA)
cPeak, chr22:41998467-41998967 (1hr,GABA)
cPeak, chr22:42119582-42120082 (0hr,nmglut)
cPeak, chr22:41998467-41998967 (0hr,GABA)
ASOC, rs2157591 (1hr,npglut)
ASOC, rs2157591 (1hr,GABA)
ASOC, rs2157591 (6hr,nmglut)
41.7
41.8
41.9
42.0
42.1
42.2
MEI1
CCDC134
SREBF2
SHISA8
TNFRSF13C
CENPM
SMIM45
SEPTIN3
WBP2NL
NAGA
SMDT1
PHETA2
NDUFA6
CYP2D6
CYP2D7
cPeak, chr5:90747663-90748163 (1hr,npglut)
ASOC, rs2460188 (6hr,nmglut)
cPeak, chr5:91119889-91120389 (0hr,GABA)
cPeak, chr5:91119889-91120389 (6hr,GABA)
cPeak, chr5:91119889-91120389 (1hr,GABA)
cPeak, chr5:90929346-90929846 (6hr,GABA)
cPeak, chr5:90929346-90929846 (1hr,GABA)
90.7
90.8
90.9
91.0
91.1
ADGRV1
cPeak, chr12:57096240-57096740 (1hr,npglut)
cPeak, chr12:57096240-57096740 (1hr,GABA)
ASOC, rs324015 (0hr,npglut)
cPeak, chr12:57096240-57096740 (0hr,GABA)
cPeak, chr12:57096240-57096740 (6hr,GABA)
cPeak, chr12:57093803-57094303 (0hr,nmglut)
cPeak, chr12:57116477-57116977 (1hr,nmglut)
cPeak, chr12:57292822-57293322 (6hr,npglut)
cPeak, chr12:57116477-57116977 (6hr,nmglut)
cPeak, chr12:57096240-57096740 (6hr,GABA)
cPeak, chr12:57096240-57096740 (1hr,npglut)
cPeak, chr12:57096240-57096740 (1hr,GABA)
cPeak, chr12:57096240-57096740 (0hr,GABA)
56.9
57.0
57.1
57.2
57.3
SDR9C7
RDH16
GPR182
ZBTB39
TAC3
MYO1A
NEMP1
NAB2
STAT6
LRP1
NXPH4
SHMT2
NDUFA4L2
STAC3
Shape
GABA genes
nmglut genes
npglut genes
SNPs
Color
0hr genes
1hr genes
6hr genes
SNPs
